# Comparative plastome analysis of Musaceae and new insights into phylogenetic relationships

**DOI:** 10.1186/s12864-022-08454-3

**Published:** 2022-03-21

**Authors:** Ning Fu, Meiyuan Ji, Mathieu Rouard, Hai-Fei Yan, Xue-Jun Ge

**Affiliations:** 1 Key Laboratory of Plant Resources Conservation and Sustainable Utilization, South China Botanical Garden, Chinese Academy of Sciences, Guangzhou, China; 2 University of Chinese Academy of Sciences, Beijing, China; 3 Bioversity International, Parc Scientifique Agropolis II, 34397 Montpellier Cedex 5, France; 4 Center of Conservation Biology, Core Botanical Gardens, Chinese Academy of Sciences, Guangzhou, China

**Keywords:** Musaceae, Plastome, Phylogeny, Divergence time, cpDNA marker

## Abstract

**Background:**

Musaceae is an economically important family consisting of 70-80 species. Elucidation of the interspecific relationships of this family is essential for a more efficient conservation and utilization of genetic resources for banana improvement. However, the scarcity of herbarium specimens and quality molecular markers have limited our understanding of the phylogenetic relationships in wild species of Musaceae. Aiming at improving the phylogenetic resolution of Musaceae, we analyzed a comprehensive set of 49 plastomes for 48 species/subspecies representing all three genera of this family.

**Results:**

Musaceae plastomes have a relatively well-conserved genomic size and gene content, with a full length ranging from 166,782 bp to 172,514 bp. Variations in the IR borders were found to show phylogenetic signals to a certain extent in *Musa*. Codon usage bias analysis showed different preferences for the same codon between species and three genera and a common preference for A/T-ending codons. Among the two genes detected under positive selection (dN/dS > 1), *ycf2* was indicated under an intensive positive selection. The divergent hotspot analysis allowed the identification of four regions (*ndhF-trnL*, *ndhF*, *matK-rps16*, and *accD*) as specific DNA barcodes for Musaceae species.

Bayesian and maximum likelihood phylogenetic analyses using full plastome resulted in nearly identical tree topologies with highly supported relationships between species. The monospecies genus *Musella* is sister to *Ensete*, and the genus *Musa* was divided into two large clades, which corresponded well to the basic number of n = x = 11 and n = x =10/9/7, respectively. Four subclades were divided within the genus *Musa*. A dating analysis covering the whole Zingiberales indicated that the divergence of Musaceae family originated in the Palaeocene (59.19 Ma), and the genus *Musa* diverged into two clades in the Eocene (50.70 Ma) and then started to diversify from the late Oligocene (29.92 Ma) to the late Miocene. Two lineages (*Rhodochlamys* and *Australimusa*) radiated recently in the Pliocene /Pleistocene periods.

**Conclusions:**

The plastome sequences performed well in resolving the phylogenetic relationships of Musaceae and generated new insights into its evolution. Plastome sequences provided valuable resources for population genetics and phylogenetics at lower taxon.

**Supplementary Information:**

The online version contains supplementary material available at 10.1186/s12864-022-08454-3.

## Background

**Fig. 1:**
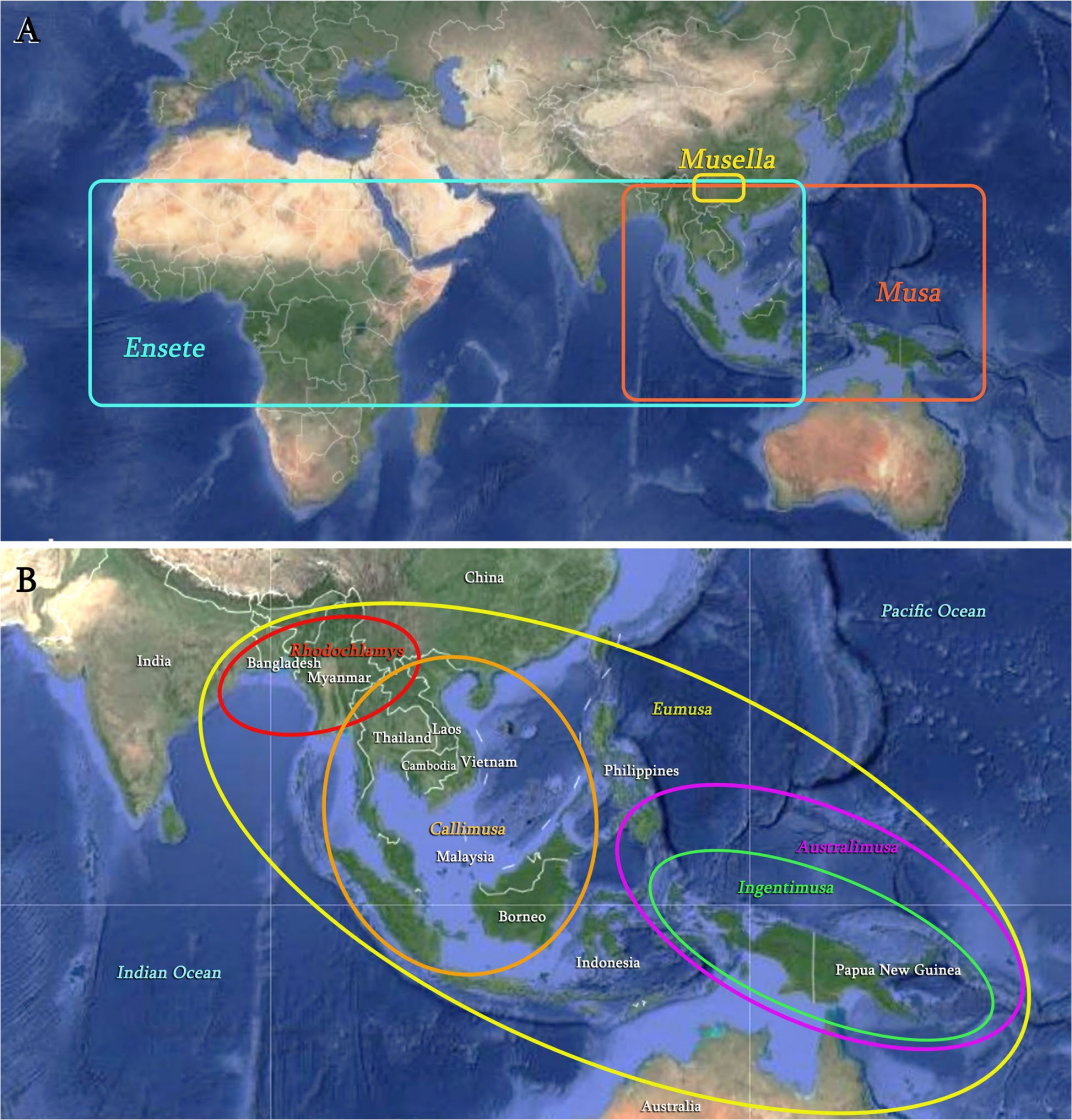
Distribution maps for (A) three genera of Musaceae and (B) five sections of genus *Musa*

Musaceae, known as the banana family, is disjunctly distributed in the tropical and subtropical regions of Asia, Africa, and Australia (Fig. 1). Three genera are commonly recognized within Musaceae, viz. *Musa* L., *Ensete* Horan., and *Musella* (Franch.) Li. The largest genus *Musa*, comprises about 70 species [1, 2] and is naturally distributed in Southeast Asia (Fig. 1, Table S1). *Ensete*, harboring 7-8 species, is sympatric with *Musa* in Asia but covers most tropical Africa [3] (Fig. 1, Table S1). The monotypic genus *Musella* is native to mountainous Southwest China [1], although its generic status was disputed [4–6] (Fig. 1, Table S1).

The genus *Musa* was established by Carolus Linnaeus in 1753 [7]. Cheesman [8] divided the genus into four sections: *Australimusa* and *Callimusa* with n = 10, *Eumusa* and *Rhodochlamys* with n = 11 chromosomes. Later, Argent [9] established the *Musa* sect. *Ingentimusa* based on a single species, *Musa ingens* N.W. Simmonds with n =7. Sections *Rhodochlamys* and *Eumusa* are closely related, having bracts that are generally sulcate, glaucous and that become revolute on fading [8]. This contrasts with species of sections *Australimusa* and *Callimusa*, which have bracts that are smooth, polished on the outside, and that do not become revolute on fading. In contrast with the pendent inflorescences with dull-colored bracts and large plants (3 m or taller) in *Eumusa*, species of sect. *Rhodochlamys* are generally smaller in stature (less than 3 m), have erect inflorescences with brightly colored bracts. Species of sect. *Callimusa* are separated from those of sect. *Australimusa* by their unique seeds, which are cylindrical or barrel-shaped and possess a large apical chamber. Seeds of species of sect. *Australimusa* are subglobose or dorsiventrally compressed and possess a small apical chamber. These five sections proved to be very useful and have been widely accepted [8–11]. Since the molecular markers were applied in plant systematics, there are many related studies on the *Musa* section assessment. For example, Wong et al. [12] used AFLP to validate this classification system. Several phylogenetic studies have been published for the Musaceae, however, none of these five sections was recovered as monophyletic [5, 6, 12–18]. Only two infrageneric clades corresponded well to the basic chromosome numbers (one clade with n = x = 11, the other with n = x = 10/9/7) [6, 17]. Häkkinen [2] reappraised the five-section system by integrating molecular phylogenetic studies and proposed two infrageneric clades classification: sect. *Musa* and sect. *Callimusa* (referring as sect. *Callimusa* Cheesman emend Häkkinen). Sect. *Rhodochlamys* was synonymized with sect. *Musa*, sect. *Australimusa* and sect. *Ingentimusa* were treated as synonyms of sect. *Callimusa* [2].

Most edible banana cultivars are from hybridization between *Musa acuminata* Colla different subspecies or with *M. balbisiana* Colla [3] and these two species are both from the sect. *Musa* [2]. A well-resolved phylogeny of Musaceae is critical for the germplasm conservation of cultivated banana ancestors and their wild relatives. However, a well-resolved phylogeny of Musaceae has been still missing. The lack of herbarium specimens and quality molecular markers limited our understanding of the phylogenetic relationships of Musaceae species. Studies with broad taxonomic coverage usually employed limited gene fragments and reconstructed phylogeny containing polytomy and low-support branches [5, 6, 17–19]. For instance, using plastid *atpB-rbcL*, *rps16*, *trnL-F* and nuclear ribosomal ITS, Li et al. [6] generated a phylogenetic tree with many polytomies though this study covered 36 species. Recently, Burgos-Hernandez et al. [18] used ITS, *trnL-trnF* and *atpB-rbcL* to conduct a biogeographic analysis of Musaceae and covered 37 species. Their resulting phylogeny also encompassed multiple low-support branches. In contrast, studies using multiple low copy nuclear genes or even whole-genome sequences on Musaceae phylogeny have in-depth gene coverage and strong internal support, but their taxonomic coverage was often sparse [20–23] since their sampled species did not even exceed 20. Thus, it is worthwile to investigate phylogenetic relationships of Musaceae in more detail with both expanded taxonomic coverage and gene sampling.

Genome skimming, an approach to sequence samples with shallow depth, is usually used to acquire the high-copy genomic fraction, such as plastome [24]. Many studies showed that the plastome significantly resolves phylogenetic relationships at lower taxonomic levels [25–29]. The plastome is maternally inherited without recombination in Musaceae [30]. They are generally comprised of four regions, namely the large single copy (LSC), the small single copy (SSC), and two inverted repeats (IRs, IRa, and IRb) [31]. Some highly variable regions in the plastome have been identified as “hotspots” and employed as useful molecular markers for phylogenetic studies [32, 33]. In recent years, although some plastome sequences ofMusaceae have been reported [23, 34–36], most species studied concentrated on a few wild bananas cultivated at botanical gardens and did not propose a comprehensive plastome analysis for the Musaceae family. In this study, we used the genome skimming approach for the assembly of the plastomes of a large panel of Musaceae species. We analyzed their plastome (1) to investigate the plastome structure variations; (2) to identify highly variable regions; and (3) to reconstruct the phylogeny of the Musaceae, and (4) to assess the divergence time of the main clades.

## Results

### Plastome features

**Fig. 2:**
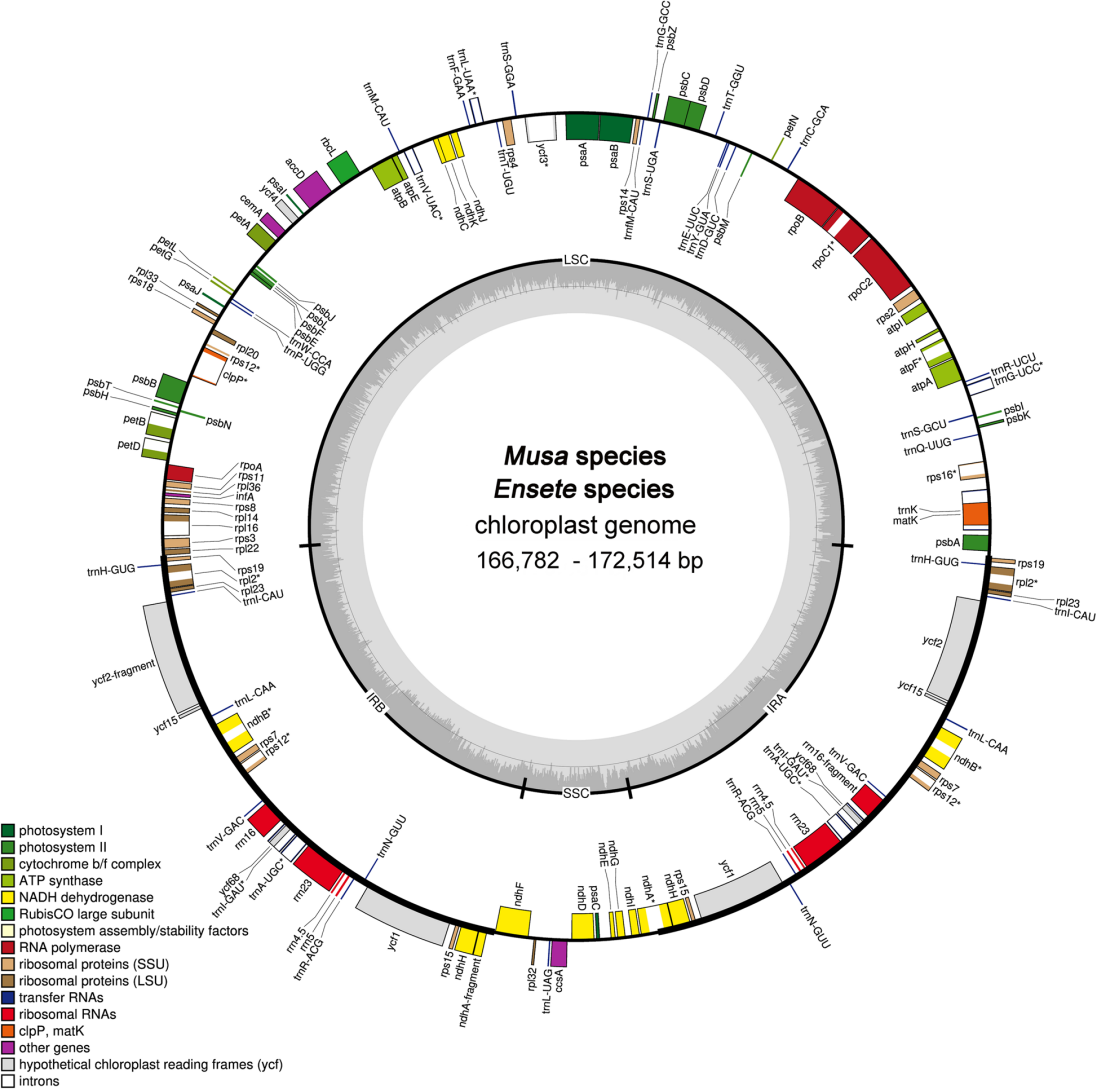
Plastome map of Musaceae species generated in this study. Genes inside the outer circle are transcribed clockwise while those outsides are transcribed counterclockwise. Genes are color-coded according to their function. Darker gray columns in the inner circle represent the GC content and the lighter gray columns accordingly correspond to the AT content. Genes marked with one asterisk (*) contain at least one intron

We analysed the structure of 49 full plastomes covering 48 species/subspecies in the Musaceae (including 45 new plastome assemblies generated for this study) (Table 1). The full-length variation of Musaceae and the genus *Musa* plastomes is approximately 5.7 kb (plastome length: 166,782-172,514 bp), with small variation in *Ensete* plastomes (163 bp, plastome length: 168,248-168,411 bp). All sequenced plastomes exhibited the typical quadripartite structure, composed of one LSC, one SSC, and two IRs (IRa and IRb) (Fig. 2). The overall GC content was nearly identical (36.5-37.1%) (Table 1). Individual plastome was annotated and followed by manual checking, resulting in a total of 113 genes, including 79 protein-coding genes, 30 transfer RNA (tRNA), and four ribosomal RNA (rRNA) genes (Fig. 2, Table S2). Among these 113 genes, 21 genes have two copies (within IR region), the remaining 92 have one single copy. Sixteen genes have one single intron, and two contain two, the left 95 genes have no intron (Table S2). The complete plastome alignment for the 48 Musaceae species illustrated that there was no genomic rearrangement (Fig. S1).

**Table 1:** Basic characteristics of the plastomes generated in this study

Species	Size (bp)	LSC (bp)	SSC (bp)	IR (bp)	GC content(%)						Number of genes	PCG	tRNA	rRNA
					**Total**	**Coding**	**Non-coding**	**LSC**	**SSC**	**IR**				
*E. glaucum*	168,248	87,832	11,144	34,636	37.1	37.4	36.7	35.5	31.3	40.0	135	89	38	8
*E. livingstonianum*	168,258	88,099	11,123	34,506	37.1	37.5	36.6	35.4	31.5	40.0	135	89	38	8
*E. superbum*	168,332	88,190	11,048	34,547	37.0	37.5	36.4	35.3	31.3	40.0	135	89	38	8
*E. ventricosum*	168,411	88,620	11,075	34,358	37.1	37.5	36.6	35.4	31.5	40.2	135	89	38	8
*M. acuminata* subsp. *banksii*	169,808	88,413	10,761	35,317	36.9	37.4	36.3	35.2	31.3	39.8	136	90	38	8
*M. acuminata* subsp. *burmannica*	169,795	88,293	10,750	35,376	36.9	37.3	36.5	35.4	31.4	39.7	136	90	38	8
*M. acuminata* subsp. *halabanensis*	169,658	88,617	11,059	34,991	36.9	37.4	36.3	35.2	31.3	39.9	136	90	38	8
*M. acuminata* subsp. *microcarpa*	170,081	88,853	10,772	35,228	36.8	37.4	36.2	35.1	31.2	39.8	136	90	38	8
*M. acuminata* subsp. *truncata*	170,137	88,747	10,772	35,309	36.9	37.4	36.3	35.2	31.3	39.8	136	90	38	8
*M. acuminata* subsp. *zebrina*	169,873	88,437	10,734	35,338	36.9	37.4	36.3	35.3	31.2	39.7	136	90	38	8
*M. aurantiaca*	170,058	88,429	10,815	35,407	36.9	37.4	36.3	35.3	31.2	39.7	136	90	38	8
*M. barioensis*	168,559	88,478	11,021	34,530	36.8	37.3	36.3	35.2	31.2	39.9	135	89	38	8
*M. basjoo*	171,853	89,746	11,739	35,184	36.5	37.3	35.6	34.8	30.2	39.7	136	90	38	8
*M. beccarii*	168,209	88,164	11,055	34,495	36.8	37.3	36.4	35.2	31.1	39.9	135	89	38	8
*M. borneensis*	168,703	88,459	11,044	34,600	36.8	37.3	36.3	35.1	31.2	39.8	135	89	38	8
*M. cheesmanii*	170,714	88,526	11,636	35,276	36.7	37.4	36.1	35.2	30.7	39.7	136	90	38	8
*M. chunii*	169,309	88,054	10,599	35,328	37.0	37.6	36.4	35.4	31.6	39.8	136	90	38	8
*M. coccinea*	166,826	87,932	10,439	34,129	37.1	37.4	36.9	35.5	31.6	40.1	134	89	38	8
*M. gracilis*	166,782	87,118	11,694	33,985	37.0	37.3	36.7	35.4	31.3	40.1	135	89	38	8
*M. ingens*	168,249	88,319	10,854	34,538	36.8	37.2	36.4	35.1	31.0	39.9	135	89	38	8
*M. jackeyi*	167,693	88,350	11,049	34,147	36.9	37.3	36.4	35.1	31.1	40.0	135	89	38	8
*M. johnsii*	167,331	87,549	11,008	34,387	37.0	37.4	36.6	35.4	31.2	40.0	135	89	38	8
*M. laterita*	170,143	88,746	10,773	35,312	36.8	37.4	36.3	35.2	31.2	39.8	136	90	38	8
*M. lokok*	166,902	86,881	11,087	34,467	37.0	37.3	36.7	35.4	31.2	39.9	135	89	38	8
*M. lolodensis*	168,542	88,330	11,060	34,576	36.8	37.3	36.3	35.2	31.1	39.9	135	89	38	8
*M. maclayi* subsp. *maclayi*	167,586	88,243	11,049	34,147	36.9	37.3	36.4	35.2	31.1	40.0	135	89	38	8
*M. mannii*	170,636	88,883	10,815	35,469	36.8	37.4	36.1	35.1	31.2	39.7	136	90	38	8
*M. nagensium*	169,758	88,418	11,082	35,129	36.7	37.3	36.0	35.0	30.8	39.8	136	90	38	8
*M. ornata*	169,896	88,673	10,851	35,186	36.8	37.4	36.2	35.1	31.1	39.9	136	90	38	8
*M. paracoccinea* J52	167,287	88,225	10,557	34,246	37.1	37.4	36.8	35.4	31.4	40.1	135	89	38	8
*M. paracoccinea* LSY001	167,601	88,304	10,589	34,354	37.0	37.3	36.6	35.2	31.2	40.1	135	89	38	8
*M. peekelii* subsp. *angustigemma*	167,660	88,282	11,084	34,147	36.9	37.3	36.4	35.1	31.1	40.0	135	89	38	8
*M. puspanjaliae*	171,298	89,386	11,476	35,218	36.6	37.3	35.9	35.0	30.4	39.7	136	90	38	8
*M. rosea*	168,495	87,364	10,541	35,295	37.1	37.4	36.7	35.5	31.7	39.8	136	90	38	8
*M. rubinea*	172,514	89,995	11,767	35,376	36.5	37.3	35.6	34.8	30.1	39.6	136	90	38	8
*M. rubra*	169,309	88,128	10,773	35,204	37.0	37.4	36.5	35.4	31.2	39.9	136	90	38	8
*M. ruiliensis*	167,806	86,945	10,413	35,224	37.1	37.4	36.9	35.6	31.9	39.9	136	90	38	8
*M. salaccensis*	167,018	87,262	11,112	34,322	37.0	37.4	36.6	35.4	31.1	40.0	135	89	38	8
*M. sanguinea*	170,502	89,201	10,943	35,179	36.8	37.4	36.1	35.1	30.9	39.8	136	90	38	8
*M. schizocarpa*	169,821	88,707	10,714	35,200	36.9	37.4	36.4	35.2	31.7	39.8	136	90	38	8
*M. siamensis*	170,101	88,718	10,793	35,295	36.9	37.4	36.3	35.2	31.2	39.8	136	90	38	8
*M. tonkinensis*	170,100	88,926	10,962	35,106	36.8	37.3	36.3	35.1	31.3	39.8	136	90	38	8
*M. troglodytarum*	167,929	88,532	11,049	34,174	36.8	37.3	36.4	35.1	31.1	40.0	135	89	38	8
*M. velutina*	169,791	89,392	11,067	34,666	36.8	37.4	36.2	35.1	31.1	39.9	135	89	38	8
*M. yunnanensis*	170,086	88,940	11,072	35,037	36.7	37.2	36.2	35.1	31.1	39.7	136	90	38	8

### IR boundary comparative analysis

The IR/LSC and IR/SSC junctions of the 49 Musaceae species were compared to explore the IR expansion/contraction (Fig. S2). No noticeable expansion or contraction was found within the four *Ensete* species. Compared to *Ensete* species, the JLA and JLB of *Musella lasiocarpa* extended into gene *rps19*. Apparent differences in IR boundaries were observed among *Musa* species. The JSB of *Musa gracilis* withdraws to the spacer of *ndhA1* and *ndhF* compared to other species from sect. *Callimusa* Cheesman emend Häkkinen, of which JSBs resided in *ndhF* (Fig. S2). On the contrary, the JSB of *Musa balbisiana* extended into the *ndhF* gene compared to other species in the sect. *Musa*. All those species from the sect. *Callimusa* Cheesman emend Häkkinen had only one copy of *rps19* gene. In contrast, those species from the sect. *Musa* had one more copy of *rps19*, except *Musa velutina.* The four junctions between LSC/IRs and SSC/IRs were confirmed with PCR-based sequencing. The assembly of the PCR product was mapped against the plastome that we generated previously and the mapping result was shown in Fig. S3. All of the IR borders could match the assemblies of PCR-based sequences.

### Codon usage preference

**Fig. 3:**
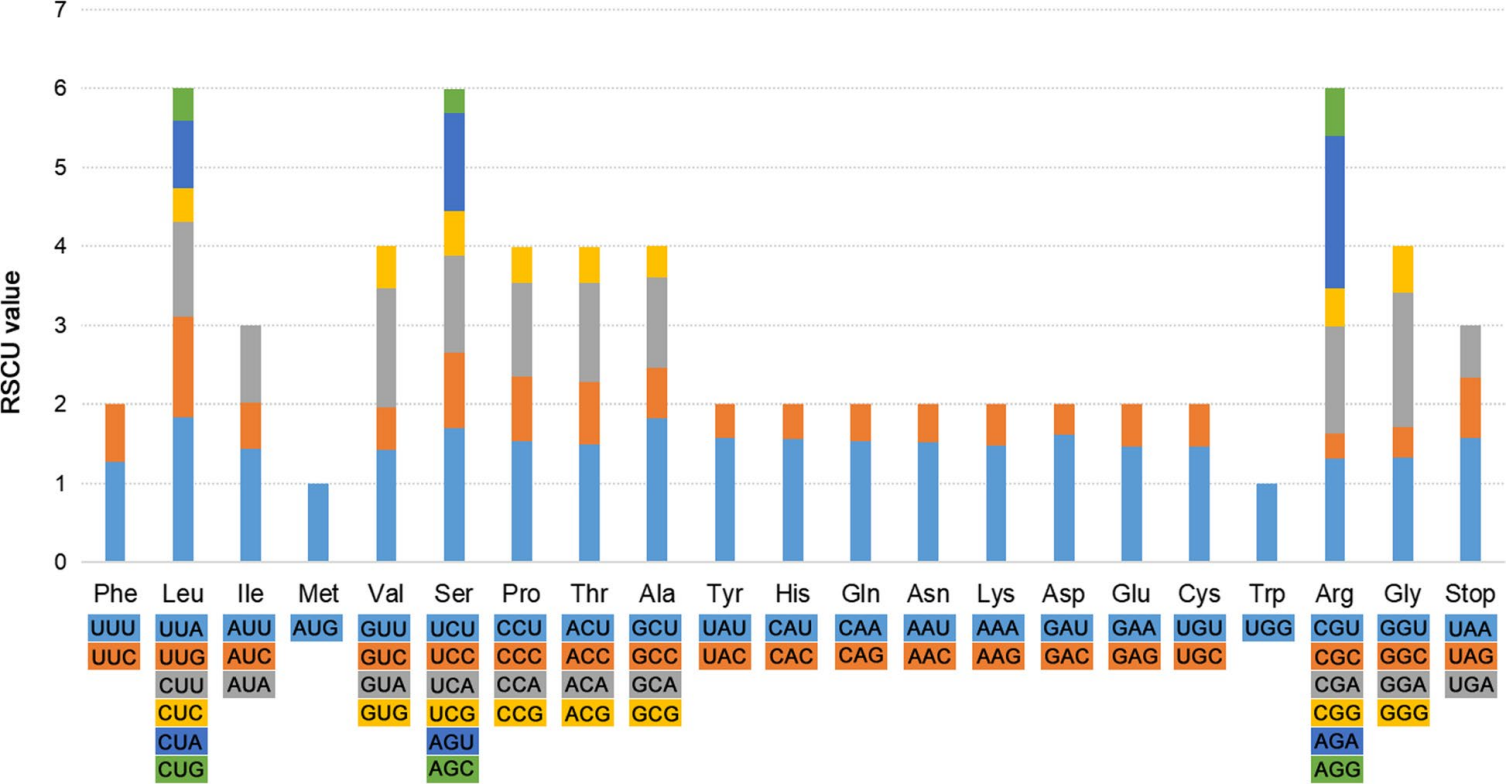
Codon content of 20 amino acids and stop codon in Musaceae plastomes. The relative synonymous codon usage (RSCU) values are shown on the y-axis

Among the 49 Musaceae plastomes, the total codons (including stop codons) ranged from 28,770 in *M. itinerans* to 29,521 in *M. yunnanensis* (Table S3). The codon frequency was relatively similar across Musaceae species (Table S4). Only methionine (Met) and tryptophan (Trp) were encoded by a single codon among all 20 amino acids encoded by 64 codons (Fig. 3). The three most frequent codons were GAA-Glu, AUU-Ile, and AAA-Lys (Table S4). The most and least abundant amino acids were leucine (Leu) and cysteine (Cys), encoded by about 10% and 1% of codons, respectively (Table S4). The relative synonymous codon usage (RSCU) values of the same codon were very similar between all plastomes of Musaceae (Table S4). The two codons with the highest RSCU values were AGA-Arg and UUA-Leu. Codons ending in T or A had RSCU > 1. In contrast, codons with C or G in the third position mostly had RSCU < 1, indicating a significant preference for codons ending with T and A, which is generally observed in the angiosperm plastomes [37, 38]. GC3 value is significantly higher than the GC2 in all Musaceae species, which supported this preference pattern (Table S3). *Musa* species exhibited higher usages in UUG, GUG, GAA, CGU, AGA, GGU, and GGA (Table S5).

### Repeat analysis

**Fig. 4:**
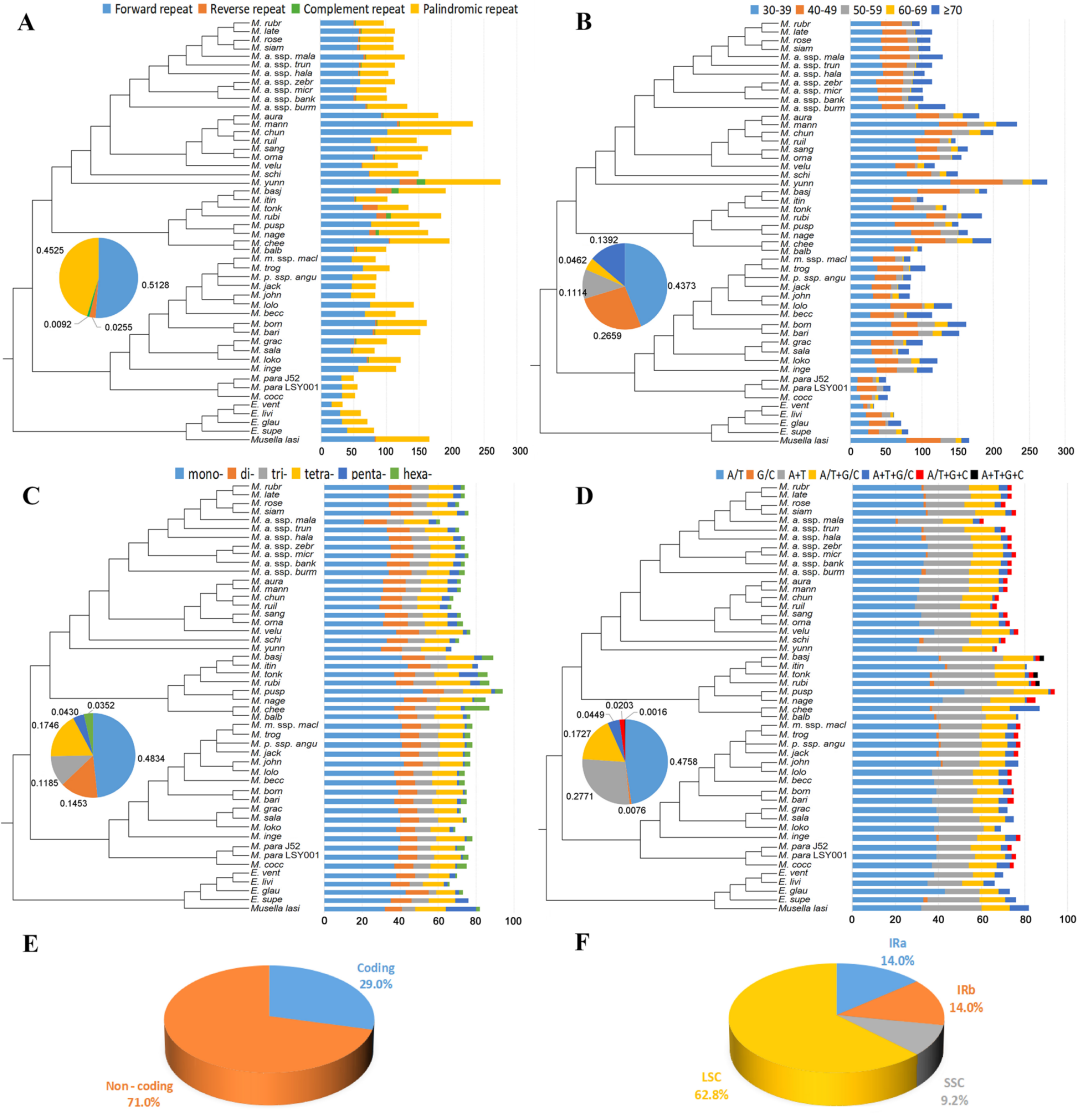
Analysis of repeat elements in Musaceae plastomes. **A** Frequency and average proportion of four types of short dispersed repeats (SDRs). Pie chart showing the average proportion of four SDRs types. **B** Frequency and average proportion of SDRs by length. **C** Frequency and average proportion of six simple sequence repeats (SSRs) types. **D** Frequency and average proportion of SSRs by base composition. **E** Average proportion of SSRs in coding and noncoding region. **F** Average proportion of SSRs in the LSC, SSC, and IR. The phylogenetic tree was infered from comlete plastome dataset

The total number of short dispersed repeats (SDRs) in the 49 Musaceae plastomes ranged from 33 (*E. ventricosum*) to 233 (*M. yunnanensis*) pairs (Fig. 4A, Table S6). There were more forward and palindromic repeats instead of reverse and complement repeats (Fig. 4A). The SDRs with 30–49 bp in length existed more widely than the repeats ≥50 bp (Fig. 4B, Table S6). The majority of the SSRs were mono-nucleotide repeats (ca. 48.34%), followed by tetra-nucleotide (ca. 17.46%), and the least was hexa-nucleotide (ca. 3.52%) (Fig. 4C, Table S7). When considering the base composition of SSRs, the most common repeats were mono-nucleotide repeats composed of A or T, accounting for about 47.58% (Fig. 4D, Table S7). Most SSRs (71%) were located in non-coding regions, while the remaining SSRs distributed in coding regions, including *rpoC2*, *rps14*, *ycf2*, *ycf1*, and *ndhH* (Fig. 4E, Table S8). Moreover, more than half of SSRs (62.8%) were found in the LSC region, only 9.2% and 28.0% were located in the SSC and the IR regions, respectively (Fig. 4F, Table S8). The total tandem repeats ranged from 36 in *M. paracoccinea* to 128 in *M. rubinea* (Fig. S4, Table S9). Most tandem repeats (61.6%) were located in non-coding regions, while the remaining tandem repeats were distributed in coding regions, including *rpoC2*, *accD*, *rpl20*, *rps11*, *ycf2*, *ycf1* (Fig. S4, Table S10).

### Selective pressure analysis

Synonymous (dS) and nonsynonymous (dN) substitution rates, as well as dN/dS, were determined for the 79 coding sequences to estimate the selective pressure acting on them (Fig. S5, Table S11). The dN and dS ranged from 0 to 0.16, and 0 to 0.59, respectively. Among the 79 CDSs, *ndhF* and *rpl32* showed relatively higher dS values (> 0.4), while *accD* and *matK* exhibited relatively higher dN values (> 0.1; Fig. S5, Table S11). For most genes (89.87%), dS was significantly greater than dN, resulting in a dN/dS value less than 0.5, suggesting a purifying selection. Two genes with relatively higher dN/dS value were identified (dN/dS > 1; *ycf1*, *ycf2* valued as 1.16 and 4.44, respectively). The null model (dN/dS = 1) was performed for *ycf1* and *ycf2*. The *P* value of Chi-square test for *ycf2* was less than 0.05, indicating an intensive positive selection. *P* value of *ycf1* was 0.4335, it suggested that *ycf1* may not be in positive selection (Table 2).

**Table 2:** Positive selection genes of Musaceae species

Gene	lnL H0	lnL HA	df	*P* value
*ycf1*	-8822.2598	-8821.6518	1	0.4355
*ycf2*	-11049.3922	-11015.8495	1	0.0000

### Sequence variability and divergent hotspots identification

Nucleotide diversity (Pi) of the 49 Musaceae plastomes ranges from 0 to 0.03282, with an average of 0.00698 (Fig. S6, Table S12). Among LSC, SSC, and IR regions, SSC and IR regions exhibit the highest and the lowest Pi value of 0.01671 and 0.00389, respectively (Table S12). Ten most variable regions with peak Pi values > 0.020 and alignment length over 600 bp were identified as divergent hotspots (Fig. S6, Table S12). The *ndhF-trnL* sequence had the highest Pi value (0.02470), followed by *ndhF*, *matK-rps16*, and *accD* (Table S12). These four hypervariable markers had more haplotypes (45 vs. 34) and higher resolution than the three universal DNA barcodes (*matK*, *rbcL*, and *trnH-psbA*) based on the ML tree (Fig. S7, Table S12). Moreover, based on the combination of the four most variable markers, many indels sites could be found within those pairwise species with the lowest K2P distance (Table S13). These indels increased the species identification rate for those closely related species.

### Phylogenetic relationships

**Fig. 5:**
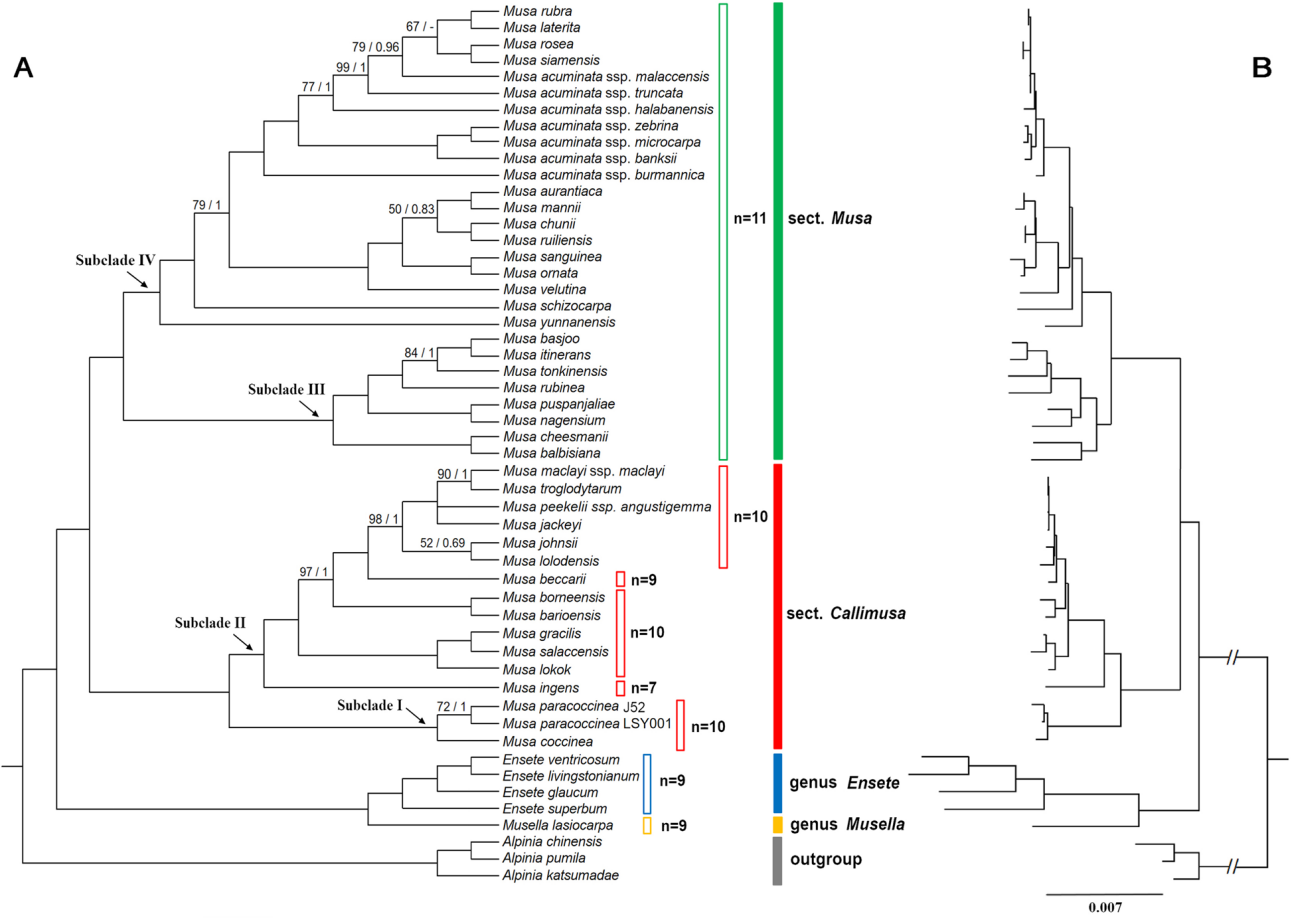
Maximum likelihood (ML) cladogram (**A**) and phylogram (**B**) of Musaceae inferred from complete plastomes using RAxML. ML bootstrap (BS) values and the posterior probabilities (PP) calculated from MrBayes are shown at nodes, except nodes with 100% BS and 1.0 PP, ‘-’indicates PP under 0.5. Clade is set to polytomy when BS<50% and PP<0.5

Our Maximum likelihood (ML) and Bayesian inference (BI) analyses generated a consistent phylogenetic tree supporting the same topological structure. The CDSs and the complete plastome dataset produced similar topology trees with only one discordance on the relationship between five species in sect. *Callimusa* (*M. borneensis*, *M. barioensis*, *M. gracilis*, *M. salaccensis*, *M. lokok*) (Fig. S8, Fig. S9). The full plastome dataset provided a better-supported phylogeny than CDSs dataset because it possessed fewer branches with bootstrap support values of less than 90%. The monospecies genus *Musella* is sister to the *Ensete* (Fig. 5). The genus *Musa* was subdivided into two large clades, which corresponded to the *Callimusa* and *Musa* Cheesman emend Häkkinen sections.

Within the sect. *Callimusa* Cheesman emend Häkkinen, the lineage of *Musa coccinea* and *M. paracoccinea* (subclade I, with support value: 100/1.0) is at the base of this section (Fig. 5). *Musa ingens* (2n = 14), the physically largest member of Musaceae, is basal to the other species of subclade II (with support value: 100/1.0). *M. beccari* (2n = 18) nested at the different species with 2n = 20, and in the basal position for the species from sect. *Australimusa*. For the sect. *Musa*, subclade III (with support value: 100/1.0) consists of *M. balbisiana*, *M. cheesmanii*, *M. basjoo*, *M. itinerans*, *M. tonkinensis*, *M. nagensium*, etc. Subcalde IV (with support value: 100/1.0) consists of two groups, one from sect. *Rhodochlamys*, and another including different *M. acuminata* subspecies. Among the subspecies of *M. acuminata*, *M. acuminata* ssp. *burmannica* is the earliest diversified subspecies. Four species, namely *M. siamensis*, *M. rosea*, *M. rubra*, and *M. laterita*, were embedded within the clade of *M. acuminata* subspecies.

### Divergence time estimation

**Fig. 6:**
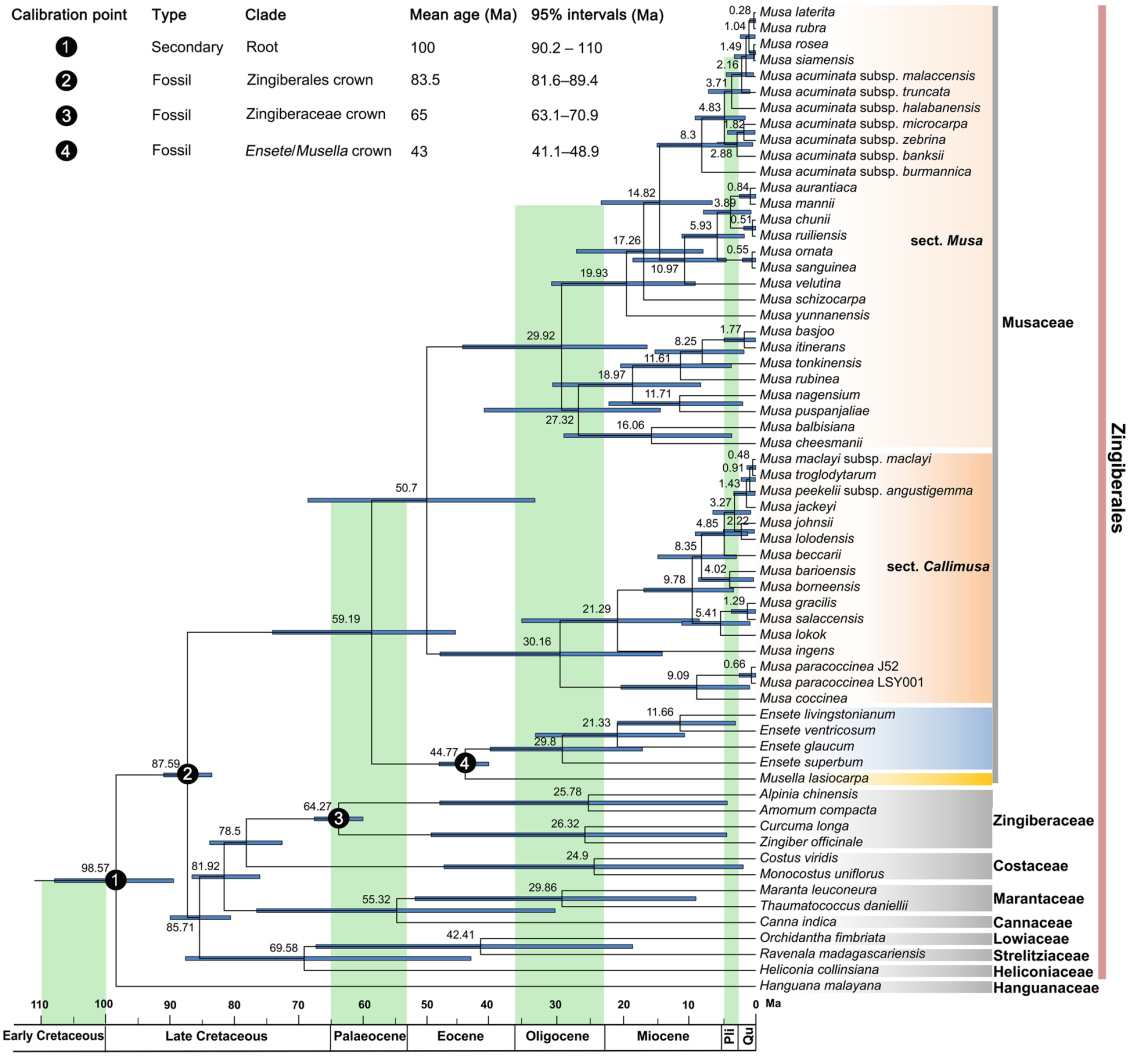
Divergence time of Musaceae obtained from BEAST 2 based on the five genes (*ccsA*, *matK*, *ndhF*, *rpoC1*, and *rpoC2*) selected by Sortadate. Mean divergence times of the nodes are shown at the nodes and the blue bars correspond to the 95% highest posterior density (HPD). The outgroup taxa are marked with gray strips

Divergence time estimates suggested that the crown node age of Musaceae was 59.19 Ma (95% HPD: 46.26-74.47 Ma) (Fig. 6). The crown node ages of *Musa* and *Ensete*-*Musella* clade were 50.70 Ma (95% HPD: 34.03-69.01 Ma) and 44.77 Ma (95% HPD: 41.14-48.80 Ma), respectively. Diversification of sect. *Musa* and sect. *Callimusa* Cheesman emend Häkkinen occurred at 29.92 Ma (95% HPD: 16.74-45.17 Ma) and 30.16 Ma (95% HPD: 14.40-48.65 Ma) during the Oligocene. Within sect. *Callimusa* Cheesman emend Häkkinen, the lineage in Malayan Peninsula/Sumatra, Borneo, and Papua Guinea and the lineage in Indochina, their diversification arose at about 9.78 Ma and 9.09 Ma, respectively. *M. acuminata* subspecies started to radiate at about 8.30 Ma. The species in sect. *Australimusa* rapidly radiated ca. 3.13 Ma.

## Discussion

### Phylogenetic relationships of Musaceae

Compared to previous phylogenetic studies on Musaceae [5, 6, 17], this study is the first one to analyze Musaceae phylogenetic relationships with density sampling using plastome-scale sequences. The resulting tree is fully resolved with substantially increased support value for several branches across the Musaceae tree (Fig. 5). The sister relationship between the genus *Musella* and *Ensete* is reassured. The genus *Musa* is well-supported into two clades, corresponding to Häkkinen’s two-section reappraisal as Sect. *Musa* and Sect. *Callimusa* Cheesman emend Häkkinen [2] that delineated the basic chromosome number of *n* = x = 11 and *n* = x =10/9/7, respectively. For the infrageneric classification in *Musa*, Cheesman [8] indicated that“the groups have deliberately been called sections rather than subgenera in an attempt to avoid the implication that they are of equal rank”. Although there are significant morphological characters and chromosome number difference between both clades, following the suggestion of Cheesman [8], Häkkinen [2] classified both clades as sect. *Musa* and sect. *Callimusa*, respectively (Fig. 5). x = 11 is most reasonable original basic number in Zingiberales [39], with x = 10, 9, 7 as a derived basic number in Musaceae. This phylogeny provided a frame to explore the chromosomal evolution in Musaceae diversification in future.

The *Callimusa* section (*senso* Häkkinen) comprises different morphological and chromosome characters (2n = 14, 18, 20) with sect. *Musa* (2n = 22), divided into 2 subclades. In congruence with Janssens et al. [17], *M. coccinea, M. paracoccinea* formed one subclade (subclade I), and is in the basal position of sect. *Callimusa* Cheesman emend Häkkinen (Fig. 5). Indeed, according to Liu et al. [40] and our cytological observation, *M. coccinea*, and *M. paracoccinea* have the same chromosome number of 2n = 20, and this lineage distributes in the Indochinese Region, especially in northern Vietnam and adjacent southwestern China. This lineage started to leave the northern Indo-Brumease region during the Oligocene and was followed by a localized diversification at the late Miocene. Several new species from sect. *Callimusa* Cheesman emend Häkkinen were reported recently in this region, i.e., *M. haekkinenii*, *M. viridis*, *M. splendida* and *M. lutea* [41], but concentrating only on their morphological description. For this study, we could not access the material but it would likely help refining species delimitation and phylogenetic relationship within the subclade and between the two subclades.

The subclade II (with support value: 100/1.0) distributes in the Malayan Peninsula/Sumatra, Borneo, and Papua Guinea, with the species diversity center in Borneo. Notably, it includes *M. beccarii* (2n = 18) and the physically largest wild banana, *M. ingens* (2n = 14), whose chromosome numbers differ from the other species in the sect. *Callimusa* (2n = 20) (Fig. 5). *M. ingens*, the only species in sect. *Ingentimusa*, was treated as one section by Argent at 1976 [9] due to its seven pairs of chromosmes. *M. ingens* distributes in the tropical montane forests of New Guinea, Indonesia. Our study sampled more *Australimusa* species than earlier phylogenetic studies [6, 17, 18, 23]. Six species (*M. jackeyi*, *M. johnsii*, *M. lolodensis*, *M. maclayi*, *M. peekelii*, *M. troglodytarum*) were sampled from the 12 *Australimusa* species [2] and formed one single cluster. Although *M. ingens* and *Australimusa* species have different chromosome numbers or seed shapes, they are sympatric with other species in subclade II, and phylogenetically nested within subclade II. Therefore, in agreement with previous studies [6, 17], we support the treatment of Häkkinen [2], that sect. *Ingentimusa* and sect. *Australimusa* should be reduced as the synonym of sect. *Callimusa*.

The *Musa* section is also subdivided into two subclades (subclades III and IV, both with support value: 100/1.0) with the species diversity center in Indo-Burma (Fig. 5). Subclade III includes banana wild relatives that share interesting features for crop improvement, such as *M. balbisiana* which is resistant to the harsh environment, *M. itinerans* immune to Foc. 4 [42], and *M. basjoo* the most cold-tolerant wild banana. *M. balbisiana* is one of the ancestors of the interspecific cultivated banana, no obvious close relatives were reported earlier [43]. Both Li et al. [6] and Janssens et al. [17] found that *M. balbisiana* is basal to the other species in the sect. *Musa*. However, its relationship with other species in this section was not resolved. Our result demonstrated that *M. balbisiana* clustered with the other *Musa* species (*M. basjoo*, *M. cheesmanii*, *M. itinerans, M. nagensium*, *M. puspanjaliae*, *M. rubinea*, *M. tonkinensis*) as one subclade (subclade III). These species distribute from the eastern Himalayas region to South China, and grow from seasonal tropical forest to temperate forest, with drought and cold tolerance. Natural crossing between them is a relatively common event [44]. Therefore, these species can represent valuable genetic resources for banana breeding. However, as banana wild relatives, they were often neglected while more conservation and characterization is needed.

*M. acuminata* species, the main wild ancestor of cultivated banana, is included in the sister subclade (subclade IV, Fig. 5). *M. acuminata* is an extremely variable species with a wide geographical distribution from Burma through Malaysia to New Guinea, Queensland, Samoa and the Philippines [44]. Among the *M. acuminata* subspecies, *M. a*. ssp. *burmannica* is the earliest diversified, consistent with the previous studies covering four *M. acuminata* subspecies based on whole genomes [22] and 72 *M. acuminata* accessions using restriction-site-associated DNA sequencing data [45]. Consistently with previous studies [5, 6, 17], we found that *M. acuminata* clustered closely with four species from sect. *Rhodochlamys*, namely *M. rubra, M. laterita, M. siamensis*, and *M. rosea*. However, contrary to Janssens et al. [17], *M. siamensis* is not nested within *M. acuminata* subspecies, and is clustered with *M. rubra*. This result reinforces recent studies that claimed *M. laterita* and *M. siamensis* as a synonym of *M. rubra* [46, 47]. Moreover, it is worth noting *M. rubra* and *M. rosea* were described based on the vouchers cultivated in the botanical garden, without evidence of their occurrence in the wild. The only wild population of *M. rubra* was reported in Manipur and Mizoram, NE India [46]. *M. rosea*, only collected in Angkor ruins in Cambodia, has long been a “lost species” [48]. The high plastome identity between these species and *M. acuminata* suggests that *M. acuminata* have provided their maternal material during hybridization. Various *Eumusa* × *Rhodochlamys* hybrids have been observed, which gave rise to considerable taxonomic confusion in poorly understood *Rhodochlamys* [44]. We, therefore, speculate that both species (*M. rubra* and *M. rosea*) are hybrids between *Musa acuminata* and species from sect. *Rhodochlamys*, but more studies are needed to verify their origin and species status.

Excluding *Musa rubra*, *M. laterita*, *M. rosea*, and *M. siamensis*, the other species from sect. *Rhodochlamys* formed one well-supported clade (support value: 100/1.0), with the common ancestor of *M. acuminata.* Although *Rhodochlamys* was morphologically characterized by the erect inflorescence and colorful bracts, this phylogenetic relationship suggests the separation of sect. *Rhodochlamys* from *Eumusa* was not clear-cut. Both Li et al. [6] and Janssens et al. [17] did not recover its monophyly due to the low resolution of few genes. This lineage experienced a recent (ca. 10.97 Ma) and rapid speciation (Figs. 5 and 6). Sect. *Rhodoclamys* species concentrate in the East Himalayas region, especially in the Assam-Burma mountain region. Reproductive isolation between *Rhodochlamys* species is slight [44]. Due to the difficult access for field investigation and rapid speciation, extending the sampling and employing more nuclear genes would provide further evidence for the evolutionary history of *Rhodoclyamys* species.

### Divergence time estimation

Correct phylogeny and divergence-time estimation are essential for evolutionary history study. With a complete chloroplast gene set, we can choose suitable genes to facilitate and optimize divergence-time estimation. The crown node age of Musaceae (59.19 Ma, Fig. 6) estimated was younger than the ages estimated by Christelová et al. [20] (69.1 Ma) and Kress et al. [49] (110 Ma), while older than in Janssens et al. [17] (51.9 Ma). The crown age of *Musa* (50.70 Ma) corresponds well with the results of Burgos-Hernandez et al. [18] (52 Ma), [20] (50.7 Ma), and Kress et al. [49] (51.4 Ma). The timing of initial diversification set at 30.16 Ma for sect. *Callimusa* Cheesman emend Häkkinen and 29.92 Ma for sect. *Musa* is similar to Christelová et al. [20] (28.7 and 27.9 Ma, respectively). The taxon sampling, calibration point setting, and DNA marker selection are important possible sources of error in divergence-time estimation [50]. Our study used more taxon sampling and DNA nucleotide to increase the divergence-time estimation accuracy. Among those studies for divergence-time estimation of Musaceae [17, 18, 20, 49], two fossils (*Spirematospermum chandlerae* and *Ensete oregonense*) were often used: *Ensete oregonense*, confirmed to be part of Musaceae [51] and *Spirematospermum chandlerae* Friis is the oldest known fossil of the Zingiberales. This study selected one more fossil (*Zingiberopsis attenuate*) and one secondary calibration point compared to other related studies [17, 18, 20, 49].

Our analyses suggest that main lineages within *Musa* diversified from the late Oligocene and accelerated at the late Miocene, and two lineages (*Australimusa* and most *Rhodochlamys* species) radiated very recently in the Pliocene /Pleistocene periods. As discussed in Burgos-Hernandez et al. [18], this time frame is consistent with the collision of India with Eurasia and the uplifts of the Qinghai-Tibetan Plateau (QTP). With the uplift of the QTP, the Asian monsoon was initiated in the late Oligocene, followed by several periods of strengthening in the Miocene (e.g., ~15 Ma & ~8 Ma) and a putative abrupt strengthening in the Pliocene/Pleistocene periods (~3 Ma) [52, 53]. The intensification of amount and seasonality of precipitation in South East Asia may have produced higher rates of diversification for various biotic lineages [54], which may have led to the evolutionary diversification of *Musa*, as demonstrated in other species from the lower altitudes of SE Asia, i.e., *Lepisorus* [54], *Pogostemon* [55], and *Primulina* [56]. The recent diversification of *Australimusa* species in the Pliocene and Pleistocene coincides with rapid orogenesis in New Guinea [57]. The orogenesis of the Central Range in New Guinea was initiated in the late Miocene, but most of the mountain uplift probably occurred since 5 Ma [54]. As found in the sect. *Petermannia* in the genus *Begonia* [58], the recent radiation in the *Australimusa* may be jointly triggered by orogenesis and associated microallopatry.

### Divergent IR borders and selective pressure analysis

Due to possessing many repetitive sequences, the size of IR regions could be variable, and their boundaries are in random dynamics in most plants [59, 60]. The contraction/expansion of IR region could bring about gene loss/addition [61, 62]. This study found that the contraction/expansion of IR region mainly existed in the boundaries of IR regions and LSC region, namely, JLA and JLB (Fig. S2). The IR borders variation showed phylogenetic signal in *Musa* to a certain extent. According to these two boundaries, the genus *Musa* can be roughly divided into two groups, i.e., sect. *Musa* and sect. *Callimusa* Cheesman emend Häkkinen. The divergences of IR borders also led to the variation of gene composition in the genus *Musa*. Specifically, within sect. *Musa*, except for *Musa velutina* with a single copy of gene *rps19*, the remaining species contain two copies of gene *rps19*. Whereas all species of sect. *Callimusa* Cheesman emend Häkkinen harbors only one copy of *rps19*, reducing the gene content to 135 (Table 1, Table S2). In addition, *M. coccinea* lost one copy of the *trnH* gene. This result is congruent with previous investigations [23]. The different copy numbers of *trnH* and *rps19* genes may hint at their gene substitution on nuclear and/or functional redundancy in the plastid [63].

Generally, variations in the synonymous mutation rate (dS) are likely to be affected by potential factors that could change the mutation rate, e.g., DNA repair. Nevertheless, the value of nonsynonymous mutation rate (dN) and dN/dS are impacted by the varied mutation rate and driven by selection regimes [64]. In our study, *ycf2* and *ycf1* were found with dN/dS value greater than 1 (Fig. S5, Table S11). The gene *ycf2* was indicated under intensive positive selection. Huang et al. [65] suggested that *ycf2* could be a useful DNA marker for estimating sequence variation and evolution in plants. *Ycf2* is one of the largest genes encoding putative membrane protein [66, 67] and was found to rapidly evolve in *Fagopyrum* [68], *Ipomoea* [69], *Ophrys* [70], *Chrysosplenium* [71], and Mimosoideae [72]. The extremely high dN/dS value (4.44) of *ycf2* indicated that this gene is a valuable marker for the adaptive evolution study of Musaceae.

### Divergent hotspots identification and molecular markers for Musaceae species

The mutations in the plastome are not universally randomly distributed along the sequence and are concentrated in certain regions referred to as the “hotspots” [73]. The highly variable hotspot regions could be used as markers to distinguish closely related species [74] and act as the taxon-specific DNA barcode. In this study, we identified ten highly variable regions (Fig. S6, Table S12). Among them, *ycf1* has been recommended as the most promising chloroplast DNA barcodes for land plants [75] and was found to harbor the greatest number of informative sites in this study. The compound region *ndhF-trnL*, which proved to have the highest Pi value here, has been considered to be the best marker for molecular studies at a low taxonomic level [76–78]. However, both *ycf1* and *ndhF-trnL* were less discriminatory when used alone since they could not provide enough haplotypes. The species identification analyses showed the better discriminatory power of the four most variable regions combined (*ndhF-trnL*, *ndhF*, *matK-rps16*, and *accD*) (Fig. S7). Therefore, we recommend these four regions to be the specific DNA barcodes for Musaceae species.

## Conclusions

This study employed the genome-skimming approach and assembled the complete plastomes of 44 Musaceae species/subspecies, providing valuable genomic resources for this family. Based on the complete plastome analysis, the relationship within Musaceae was resolved with high branch support. In addition, the comparative analysis of plastomes revealed variable regions, which could be used as Musaceae-specific DNA markers. All the obtained genomic resources will contribute to future studies in species identification, population genetics, and germplasm conservation of Musaceae.

## Materials and methods

### Taxon sampling, DNA extraction, and sequencing

The taxon sampling contains 49 accessions of Musaceae species/subspecies, representing four *Ensete* species (four accessions), 43 *Musa* species/subspecies (44 accessions), and one *Musella* species (one accession) (Table S14). Among these 49 Musaceae plastomes, 45 plastomes of 44 species/subspecies representing two genera (*Musa* and *Ensete*) were generated by the current study. Due to the sample collection challenges, 22 of 37 species from sect. *Callimusa* Cheesman emend Häkkinen could not be included in this study. Fifteen plastomes from other eight families were downloaded from NCBI for analysis. Sixty-four plastomes were used in the current study (Table S14). For data quality consistency, we dropped the plastome of *Musa textilis*, which presents a distinct short plastome compared to other *Musa* species (GenBank accession number: NC_022926.1, length 161,347 bp). Total genomic DNA was extracted from silica-dried materials using CTAB protocol [79]. The quality and concentrations of the DNA were assessed using agarose gel electrophoresis and a Qubit 3.0 Fluorometer (Life Technologies). We constructed sequencing libraries using the TruePrep DNA Library Prep Kit V2 for Illumina (Vazyme, TD501). Library lengths were evaluated with the High Sensitivity NGS Fragment Analysis Kit (Advanced Analytical Technologies, Ankeny, IA) on the Fragment Analyzer (Advanced Analytical Technologies). Lengths of all libraries ranged from 300 to 450 bp and were pooled together at equimolar ratios. Libraries were subjected to 150 bp paired-end sequencing on an Illumina X Ten platform (BGI, Wuhan, China). On average, approximately 3 Gb of clean NGS data were obtained for each sample. All raw reads data were submitted into the Sequence Read Archive (SRA) under BioProject PRJNA530661.

### Plastome assembly and annotation

Raw reads were trimmed, and adaptors were removed using Trimmomatric v. 0.36 [80]. The quality of filtered reads was assessed using FastQC (http://www.bioinformatics.babraham.ac.uk/projects/fastqc) to assure adaptors and bases below PHRED 30 were removed. We employed NOVOPlasty v. 4.2.1 [81] for the assembly of plastomes by providing *Musa balbisiana* as the reference (GenBank accession number NC_028439), and all parameters were kept as default settings (see https://github.com/ndierckx/NOVOPlasty). To confirm the result reliability of the assembling, we also used the toolkit GetOrganelle [82] to assemble the plastomes, and the parameter settings followed the online manual (see https://github.com/Kinggerm/GetOrganelle). In rare cases, when NOVOPlasty and GetOrganelle failed to obtain a complete plastome, reads were mapped against the non-overlapping contigs from NOVOPlasty to extend their ends to close the gap in Geneious, performing with medium-low sensitivity for 100 iterations.

Two independent approaches were applied to annotate these 45 plastomes. Firstly, the annotation of the plastome sequences was performed with GeSeq [83], choosing the plastome of *Musa acuminata* ssp. *malaccensis* (HF677508) as the reference genome. In the meantime, ARAGORN was selected as a third party to annotate tRNA. Secondly, we use MAFFT v. 7.388 [84, 85] to align and annotate these plastome sequences using the “Annotation Transfer” option with *Musa itinerans* (NC_035723) as a reference in Geneious. The annotation results from GeSeq and Geneious were subsequently compared and manually integrated. The plastome maps were drawn using OGDRAW [86]. Newly generated plastomes were submitted to GenBank (see Table S14 for accession numbers).

### Comparative plastome analyses for 49 Musaceae plastomes

The boundaries between the four plastome regions, i.e., LSC/IRb (JLB), SSC/IRb (JSB), SSC/IRa (JSA), and LSC/IRa (JLA), were inspected with the online program IRscope [87]. According to the phylogeny generated in this study (Fig. 5), we chose 17 representative species for confirming the IR region expansion/contraction. The four junctions between LSC/IRs and SSC/IRs of the 17 species were confirmed with PCR-based product sequencing. Target DNA regions were amplified in 25 µl reactions containing 10 ng (1 µl) template DNA, dNTP mixture 2 µl, 10 × LA PCR Buffer 2.5 µl, 0.5 µl of each primer, and 18.5 µl ddH_2_O. The primer pairs designed and used for PCR in this study were listed in Table S15. PCR products were bi-directionally sequenced by GENEWIZ Biotechnology Co., Ltd. (Suzhou, China). The sequences were submitted to the Science DB (available at https://www.10.11922/sciencedb.01436), and the accession number were listed in Table S16.

Codon usage analysis for protein-coding genes (PCGs) was conducted in DnaSP v. 6.12.03 [88]. PCGs were extracted and concatenated in Geneious before being imported to DnaSP for analysis. The relative synonymous codon usage (RSCU) values were calculated to measure the usage bias of synonymous codons. Other three indices, including the effective number of codons (ENC), codon bias index (CBI), GC content of the synonymous second (GC2) and third codons positions (GC3), were also computed to assess the extent of the codon usage bias.

The online program REPuter [89] was used to detect short dispersed repeats (SDRs), with the parameters setting as follows: (1) Hamming distance of 3; (2) maximum computed repeats of 500; (3) minimum repeat size of 30 bp. Besides, tandem repeats (≥ 10 bp) were calculated with the online program Tandem Repeats finder (http://tandem.bu.edu/trf/trf.html). Three alignment parameters, i.e., match, mismatch, and indel were kept as two, seven, and seven. The minimum alignment score was set to 80 and the maximum period size to 500. Simple sequence repeats (SSRs) were identified in MISA-web [90]. The minimum number of repetitions was set to 10, 5, 4, 3, 3, and 3 for mono, di-, tri-, tetra-, penta- and hexa-nucleotide repeats. The Maximum length of sequence between two SSRs to register as compound SSR was set 0. Mauve v1.1.1 [91], a plugin within Geneious, was applied to detect the genome rearrangements and inversions among 49 Musaceae plastomes.

### Nucleotide substitution rate analysis

Seventy-nine coding sequences (CDSs) were individually extracted from 49 Musaceae plastomes and separately aligned using “Translation Align” tool in Geneious. Nonsynonymous (dN) and synonymous (dS) substitution rates and the ratio of nonsynonymous to synonymous rates (dN/dS) were calculated using CODEML option in PAML v.4.9 [92]. The phylogeny generated from CDSs dataset was used as the constraint tree. The parameters in CODEML control file were set as follow: (1) F3 × 4 model for codon frequencies; (2) “model = 0” for allowing a single dN/dS value to vary among branches; (3) “cleandata = 1” to remove gaps; (4) default settings for other parameters (as alternative model, “fix_omega = 0” and “omega = 2”) [64]. For the potential positive selection gene, a null model (set “fix_omega = 1” and “omega = 1” in the control file) was additionally performed following Xiong et al. [93]. LRT were used to test model fit and a Chi-square test was conducted to calculate the *P* value.

### Sequence divergence analysis

A sliding window analysis was conducted in DnaSP v. 6.12.03 [88] to locate genomic regions with a high frequency of variation. The alignment of 49 Musaceae plastomes was generated in MAFFT (with default settings) and used as the input file. The window length and step size were set to 600 bp and 200 bp, respectively. Those regions with nucleotide diversity (Pi) values higher than 0.020 and alignment length longer than 600 bp were extracted from the alignment and analyzed individually to estimate their characteristics. The pairwise distance was calculated using Kimura 2-parameter (K2P) distance in MEGA 7 [94]. Indel polymorphism analysis was conducted in DnaSP v. 6.12.03.

### Phylogenetic analysis

For the phylogenetic analysis of Musaceae, two datasets (coding plastid sequences (CDSs) and the complete plastome sequence) were generated. A total of 49 Musaceae plastomes representing 48 species/subspecies were used, including 45 plastomes generated in this study and four downloaded from NCBI (Table S14). Three *Alpinia* species with plastome in GenBank were added as outgroup (Table S14). The 79 coding plastid sequences were combined, followed by multiple sequence alignment (MSA). For the complete plastome sequence dataset, the IRa was removed and served as inputs for MSA. All alignments were performed using MAFFT [95] and then manually checked in Geneious. We used Modeltest-NG 0.1.6 [96] to determine an optimal nucleotide substitution model under the corrected Akaike Information Criterion (AICc) for each dataset. All the ML analyses were performed in RAxML v8.2.12 [97] by assigning the GTRGAMMA model, and 1,000 rapid bootstrap replicates were run to evaluate the support values for each node. All the BI analyses were conducted in MrBayes v. 3.2.6 [98], and the best-fit models selected for CDSs dataset and the complete plastome sequence dataset were both GTR+I+G. Two MCMC runs were performed with five million generations and four chains, sampling every 5,000 generations and discarding the 25% as burn-in. For the CDSs dataset, best-fit partitioning scheme (Table S17) was determined by PartitionFinder 2 [99], and an additional ML analyse was performed using IQ-TREE [100] with 1000 ultrafast bootstraps [101].

### Molecular clock dating

The divergence time of Musaceae was estimated using BEAST v2.6.4 [102]. To incorporate multiple fossil calibration points and reduce the bias imported from a single calibration point, the divergence time was estimated by including the whole Zingiberales. SortaDate [50] was used to choose genes suitable for divergence-time estimation. This package determines which gene trees are clock-like, have the least topological conflict with the species tree, and have informative branch lengths. The ML tree generated from the complete plastome sequence dataset was used as an input species tree. As the result of SortaDate, the final screened genes were *ccsA*, *matK*, *ndhF*, *rpoC1*, and *rpoC2*. We selected optimal nucleotide substitution models for each of the five genes using Modeltest-NG 0.1.6 [96] under the AICc. These were identified as GTR+G4 for *ccsA*, *matK*, *rpoC1*, *rpoC2*, and GTR+I+G4 for *ndhF*.

In BEAST, the newick ML tree of Zingiberales inferred from complete plastome sequences was used as a starting tree due to its more robust phylogenetic resolution. Clock models were linked, while site models were unlinked for each gene. The uncorrelated log-normal distribution relaxed molecular clock model was selected with the Yule model as the tree prior. MCMC run was set to 100 million generations, sampling every 10,000 generations. BEAST 2 output was assessed in Tracer 1.7.2 [103] to evaluate convergence and ensure an effective sample size for all parameters surpassing 200. TreeAnnotator v2.6.4 was used to annotate the maximum clade credibility tree after removing the first 20% of samples as burn-in.

Three fossil records and one secondary calibration point were used in this divergence time estimation. *Spirematospermum chandlerae* [104] was used to calibrate the crown age of order Zingiberales with a mean age of 83.5 Ma. *Zingiberopsis attenuate* [105] was applied as a mean age of 65 Ma for the crown node of the Zingiberaceae family. Then *Ensete oregonense* [106] was used to calibrate the crown age of *Ensete* and *Musella* clade with a mean age 43 Ma. Each fossil calibration point was assumed to follow a normal distribution with a standard deviation of 2 and an offset of 2, resulting in 81.6–89.4, 63.1–70.9, and 41.1–48.9 Ma 95% intervals, respectively. The secondary calibration point was generated based on previous studies on Monocots [107, 108]. It was placed on the stem node of Zingiberales with a normal distribution as a mean age of 100 Ma and a broad standard deviation of 5 (95% intervals 90.2 – 110 Ma).

## Supplementary Information

**Additional file 1: Table S1. **Classification and species list of Musaceae.

**Additional file 2: Table S2. **List of genes present in the plastomes of Musaceae species generated in this study.

**Additional file 3: Table S3. **The indexes of the codon usage bias in Musaceae plastomes.

**Additional file 4: Table S4. **Codon usage in Musaceae plastomes.

**Additional file 5: Table S5. **Codons exhibited higher usages (RSCU) and lower usages (RSCU) in *Musa*compared to *Ensete* and *Musella.*

**Additional file 6: Table S6. **Frequency of short dispersed repeats (SDRs).

**Additional file 7: Table S7. **Number of simple sequence repeats (SSRs) in Musaceae plastomes.

**Additional file 8:** **Table S8. **Distribution of simple sequence repeats (SSRs) in Musaceae plastomes.

**Additional file 9:** **Table S9. **Frequency of tandem repeats by length.

**Additional file 10:** **Table S10. **Distribution of tandem repeats in Musaceae plastomes.

**Additional file 11:** **Table S11. **The estimation of substitution rate and dN/dS.

**Additional file 12:** **Table S12. **Variability of 23 regions in Musaceae.

**Additional file 13:** **Table S13. **The indel polymorphism of some pairwise species with minimal interspecific K2P distance based on the combination of four most variable markers.

**Additional file 14: Table S14.** List of taxa and sources of plant material analyzed, and GenBank accession numbers of plastome of taxa used in the present study.

**Additional file 15: Table S15.** The primers used for PCR in current study.

**Additional file 16: Table S16.** The sequences of PCR products generated in this study with their accession names in Science DB (available at https://www.doi.org/10.11922/sciencedb.01436).

**Additional file 17: Table S17. ** Partition scheme of 79 coding sequences (CDSs) used in this study.

**Additional file 18: Figure S1.** Genome rearrangement events of 49 Musaceae plastomes.

**Additional file 19: Figure S2.** Comparison of four IR borders among Musaceae plastomes.

**Additional file 20: Figure S3.** The verification of the four IR borders with PCR-based product sequencing.

**Additional file 21: Figure S4.** Analysis of tandem repeats in Musaceae plastomes.

**Additional file 22: Figure S5.** The synonymous (dS), nonsynonymous (dN) substitution rates and dN/dS of 79 plastid protein-coding genes (PCG) in Musaceae plastomes.

**Additional file 23: Figure S6.** Sliding window analysis of Musaceae plastomes (window length: 600bp; step size: 200bp).

**Additional file 24: Figure S7.** ML trees for Musaceae inferred from combination of four most variable regions and three universal plant DNA barcodes combination.

**Additional file 25: Figure S8.** Topological comparison between the phylogenies infered from (A) complete plastome dataset and (B) 79 CDS dataset.

**Additional file 26: Figure S9.** Maximum likelihood (ML) cladogram inferred from partitioned CDSs dataset using IQ-TREE.

## Data Availability

Annotated plastomes have been deposited in GenBank and raw sequence data in the NCBI SRA (see Table S14, for accession numbers). The plastome alignment, phylogeny and other data that support the findings of this study are openly available in Science Data Bank at https://www.10.11922/sciencedb.01225 and https://www.10.11922/sciencedb.01436.

## Abbreviations

AICc: corrected Akaike Information Criterion
CTAB: cetyl trimethyl ammonium bromide
bp: Base pairs
Gb: Gigabases
HPD: Highest posterior density
IR: Inverted repeat region
JLA: LSC/IRa junction
JLB: LSC/IRb junction
JSA: SSC/IRa junction
JSB: SSC/IRb junction
kb: kilobases
LSC: Large single-copy region
Ma: Million years ago
MCMC: Markov chain Monte Carlo
ML: Maximum likelihood
MSA: Multiple sequence alignment
NCBI: National Center for Biotechnology Information
NGS: Next generation sequencing
OGDRAW: OrganellarGenomeDRAW
PCG: Protein coding genes
rRNA: Ribosomal RNA
SDR: Short dispersed repeat
SSC: Small single-copy region
SSR: Simple sequence repeat
tRNA: Transfer RNA
