# Supplementary material for: Comparative plastome analysis of Musaceae and new insights into phylogenetic relationships
Source: BMC Genomics. 2022 Mar 21;23:223. doi: 10.1186/s12864-022-08454-3 (PMC8939231; doi:10.1186/s12864-022-08454-3)
Supplement: Supplementary file 1 — Additional file 1: Table S1. Classification and species list of Musaceae. [file 12864_2022_8454_MOESM1_ESM.docx]

| **Table S1** Classification and species list of Musaceae | | | | |
| --- | --- | --- | --- | --- |
| **Genus** | **Section**  (Cheesman, 1947; Argent, 1976） | **Section**  **(**Häkkinen, 2013) | **Species/subspecies** | **Chromosome number** |
| *Musa* L.  (80 sp./ssp.) | *Eumusa*  (30 sp.) | *Musa* | *Musa acuminata* Colla *ssp. banksia* | 2n = 22 |
|  |  | (36 sp.) | *Musa acuminata* Colla *ssp. burmannicoides* | 2n = 22 |
|  |  |  | *Musa acuminata* Colla *ssp. burmannica* | 2n = 22 |
|  |  |  | *Musa acuminata* Colla *ssp. malaccensis* | 2n = 22 |
|  |  |  | *Musa acuminata* Colla *ssp. microcarpa* | 2n = 22 |
|  |  |  | *Musa acuminata* Colla *ssp. truncate* | 2n = 22 |
|  |  |  | *Musa acuminata* Colla *ssp. siamea* | 2n = 22 |
|  |  |  | *Musa acuminata* Colla *ssp. zebrina* | 2n = 22 |
|  |  |  | *Musa acuminata* Colla ssp. *errans* | 2n = 22 |
|  |  |  | *Musa acuminata* Colla ssp. *halabanensis* (Meijer) M. Hotta | 2n = 22 |
|  |  |  | *Musa balbisiana* Colla | 2n = 22 |
|  |  |  | *Musa basjoo* Sieb. & Zucc. | 2n = 22 |
|  |  |  | *Musa celebica* K. Schum | Not counted |
|  |  |  | *Musa cheesmanii* N.W. Simmonds | 2n = 22 |
|  |  |  | *Musa griersonii* Noltie | Not counted |
|  |  |  | *Musa indandamanensis* L. J. Singh | Not counted |
|  |  |  | *Musa insularimontana* Hayata | Not counted |
|  |  |  | *Musa itinerans* Cheesman | 2n = 22 |
|  |  |  | *Musa lanceolata* K. Schum. | Not counted |
|  |  |  | *Musa nagensium* Prain | 2n = 22 |
|  |  |  | *Musa ochracea* K. Sheph. | 2n = 23 |
|  |  |  | *Musa schizocarpa* N. W. Simmonds | 2n = 22 |
|  |  |  | *Musa sikkimensis* Kurz | 2n = 22 |
|  |  |  | *Musa thomsonii* A. M. Cowan & Cowan | Not counted |
|  |  |  | *Musa tonkinensis* R. V. Valmayor, L. D. Danh & Häkkinen | 2n = 22 |
|  |  |  | *Musa yunnanensis* Häkkinen & H. Wang | Not counted |
|  |  |  | *Musa flaviflora* N. W. Simmonds | Not counted |
|  |  |  | *Musa puspanjaliae* R. Gogoi & Häkkinen | 2n = 22 |
|  |  |  | *Musa shankarii* Subba Rao & Kumari | Not counted |
|  |  |  | *Musa tomentosa* K. Schum. | Not counted |
|  |  |  | *Musa yamiensis* C. L. Yeh & J. H. Chen | Not counted |

**Table S1** Continued

| **Genus** | **Section**  (Cheesman, 1947; Argent, 1976） | | **Section**  **(**Häkkinen, 2013) | **Species/subspecies** | **Chromosome number** |
| --- | --- | --- | --- | --- | --- |
|  | *Rhodochlamys*  (14 sp.) |  | | *Musa aurantiaca* G. Mann ex Baker | 2n = 22 |
|  |  |  | | *Musa kattuvazhana* K. C. Jacob | Not counted |
|  |  |  | | *Musa laterita* Cheesman | 2n = 22 |
|  |  |  | | *Musa mannii* H. Wendl. ex Baker | 2n = 22 |
|  |  |  | | *Musa ornata* Roxb. | 2n = 22 |
|  |  |  | | *Musa rosea* Baker | 2n = 22 |
|  |  |  | | *Musa rubinea* Häkkinen & C.H. Teo | Not counted |
|  |  |  | | *Musa rubra* Kurz | 2n = 22 |
|  |  |  | | *Musa ruiliensis* W. N. Chen, X. J. Ge & Häkkinen | 2n = 22 |
|  |  |  | | *Musa siamensis* Häkkinen & Rich. H. Wallace | 2n = 22 |
|  |  |  | | *Musa velutina* H. Wendl. & Drude | 2n = 22 |
|  |  |  | | *Musa chunii* Häkkinen | Not counted |
|  |  |  | | *Musa zaifui* Häkkinen & H. Wang | Not counted |
|  |  |  | | *Musa sanguinea* Hook. f. | 2n = 22 |
|  | *Callimusa*  (25 sp.) | *Callimusa* (37 sp.) | | *Musa arfakiana* Argent | Not counted |
|  |  |  | | *Musa azizii* Häkkinen | Not counted |
|  |  |  | | *Musa barioensis* Häkkinen | Not counted |
|  |  |  | | *Musa bauensis* Häkkinen | Not counted |
|  |  |  | | *Musa beccarii* N.W. Simmonds | 2n = 18 |
|  |  |  | | *Musa borneensis* Becc. | 2n = 20 |
|  |  |  | | *Musa coccinea* Andrews | 2n = 20 |
|  |  |  | | *Musa exotica* R.V. Valmayor | 2n = 20 |
|  |  |  | | *Musa gracilis* Holttum | 2n = 20 |
|  |  |  | | *Musa hirta* Becc. | 2n = 20 |
|  |  |  | | *Musa haekkinenii* N.S.Lý & Haev. | Not counted |
|  |  |  | | *Musa lawitiensis* Nasution & Supard. | 2n = 20 |
|  |  |  | | *Musa lokok* Geri & Ng | Not counted |
|  |  |  | | *Musa lutea* R.V. Valmayor, L.D. Danh & Häkkinen | 2n = 20 |
|  |  |  | | *Musa monticola* Argent | 2n = 20 |
|  |  |  | | *Musa muluensis* M. Hotta | 2n = 20 |
|  |  |  | | *Musa paracoccinea* A.Z. Liu & D.Z. Li | 2n = 20 |
|  |  |  | | *Musa sakaiana* Meekiong, Ipor & Tawan | Not counted |

**Table S1** Continued

| **Genus** | **Section**  (Cheesman, 1947; Argent, 1976） | **Section**  **(**Häkkinen, 2013) | **Species/subspecies** | **Chromosome number** |
| --- | --- | --- | --- | --- |
|  |  |  | *Musa salaccensis* Backer | 2n = 20 |
|  |  |  | *Musa splendida* A. Chev. | 2n = 20 |
|  |  |  | *Musa tuberculata* M. Hotta | 2n = 20 |
|  |  |  | *Musa violascens* Ridl. | 2n = 20 |
|  |  |  | *Musa viridis* R.V. Valmayor, L. D. Danh & Häkkinen | Not counted |
|  |  |  | *Musa voonii* Häkkinen | 2n = 20 |
|  | *Australimusa* (11 sp.) |  | *Musa boman* Argent | Not counted |
|  |  |  | *Musa bukensis* Argent | 2n = 20 |
|  |  |  | *Musa fitzalanii* F. Muell. | Not counted |
|  |  |  | *Musa jackeyi* W. Hill | 2n = 20 |
|  |  |  | *Musa johnsii* Argent | 2n = 20 |
|  |  |  | *Musa lolodensis* Cheesman | 2n = 20 |
|  |  |  | *Musa maclayi* Mikl.-Maclay | 2n = 20 |
|  |  |  | *Musa peekelii* Lauterb. | 2n = 20 |
|  |  |  | *Musa textilis* Née | 2n = 20 |
|  |  |  | *Musa troglodytarum* L. | 2n = 20 |
|  |  |  | *Musa juwiniana* Meekiong, Ipor & Tawan | Not counted |
|  | *Ingentimusa*  (1 sp.) |  | *Musa ingens* N. W. Simmonds | 2n = 14 |
| *Ensete* Horan.  (8 sp.) |  |  | *Ensete glaucum* (Roxb.) Cheesman | 2n = 18 |
|  |  |  | *Ensete homblei* (Bequaert ex De Wild.) Cheesman | 2n = 18 |
|  |  |  | *Ensete livingstonianum* (J.Kirk) Cheesman | 2n = 18 |
|  |  |  | *Ensete perrieri* (Claverie) Cheesman | 2n = 18 |
|  |  |  | *Ensete superbum* (Roxb.) Cheesman | 2n = 18 |
|  |  |  | *Ensete ventricosum* (Welw.) Cheesman | 2n = 18 |
|  |  |  | *Ensete wilsonii* (Tutcher) Cheesman | 2n = 18 |
|  |  |  | *Ensete gilletii* (De Wild.) Cheesman | 2n = 18 |
| *Musella* (Franch.) Li  (1 sp.) |  |  | *Musella lasiocarpa* (Franch.) H. W. Li | 2n = 18 |
