# Supplementary material for: Comparative plastome analysis of Musaceae and new insights into phylogenetic relationships
Source: BMC Genomics. 2022 Mar 21;23:223. doi: 10.1186/s12864-022-08454-3 (PMC8939231; doi:10.1186/s12864-022-08454-3)
Supplement: Supplementary file 2 — Additional file 2: Table S2. List of genes present in the plastomes of Musaceae species generated in this study. [file 12864_2022_8454_MOESM2_ESM.docx]

| **Table S2** List of genes present in the plastomes of Musaceae species generated in this study | | |
| --- | --- | --- |
| **Group of gene** | **Genes name** | **Amount** |
| Photosystem I | *psaA, psaB, psaC, psaI, psaJ* | 5 |
| Photosystem II | *psbA, psbB, psbC, psbD, psbE, psbF, psbH, psbI, psbJ, psbK, psbL, psbM, psbN, psbT, psbZ* | 15 |
| Photosystem assembly factors | *ycf3**, ycf4* | 2 |
| Cytochrome b/f complex | *petA, petB*, petD*, petG, petL, petN* | 6 |
| ATP synthase complex | *atpA, atpB, atpE, atpF*, atpH, atpI* | 6 |
| NADH dehydrogenase complex | *ndhA* (×2)*, ndhB** (×2)*, ndhC, ndhD, ndhE, ndhF, ndhG, ndhH* (×2)*, ndhI, ndhJ, ndhK* | 14 |
| Large subunit of RuBisCO | *rbcL* | 1 |
| Maturase | *matK* | 1 |
| RNA polymerase subunits | *rpoA, rpoB, rpoC1*, rpoC2* | 4 |
| Small subunit ribosomal proteins | *rps2, rps3, rps4, rps7* (×2)*, rps8, rps11, rps12** (×2)*, rps14, rps15* (×2)*, rps16*, rps18, rps19* (×1/×2) | 15/16 |
| Large subunit ribosomal proteins | *rpl2** (×2)*, rpl14, rpl16*, rpl20, rpl22, rpl23* (×2)*, rpl32, rpl33, rpl36* | 11 |
| Subunit of acetyl-CoA-carboxylase | *accD* | 1 |
| Subunit of Clp-protease | *clpP*** | 1 |
| Translation initiation factor | *infA* | 1 |
| Inner envelope membrane protein | *cemA* | 1 |
| Cytochrome c biogenesis protein | *ccsA* | 1 |
| Genes of unknown function | *ycf1* (×2)*, ycf2* (×2) | 8 |
| Ribosomal RNAs | *rrn4.5* (×2)*, rrn5* (×2)*, rrn16* (×2)*, rrn23* (×2) | 8 |
| Transfer RNAs | *trnA-UGC** (×2)*, trnC-GCA, trnD-GUC, trnE-UUC, trnF-GAA, trnfM-CAU, trnG-GCC, trnG-UCC*, trnH-GUG* (×2, ×1 in *M. coccinea* )*, trnI-CAU* (×2)*, trnI-GAU** (×2)*, trnK-UUU*, trnL-CAA* (×2)*, trnL-UAA*, trnL-UAG, trnM-CAU, trnN-GUU* (×2)*, trnP-UGG, trnQ-UUG, trnR-ACG* (×2)*, trnR-UCU, trnS-GCU, trnS-GGA, trnS-UGA, trnT-GGU, trnT-UGU, trnV-GAC* (×2)*, trnV-UAC*,  trnW-CCA,  trnY-GUA* | 37/38 |
| Total |  | 134-136 |

Note: Genes marked with one asterisk (*) contain one intron; two asterisks (**) represent two introns.
