# Supplementary material for: Comparative plastome analysis of Musaceae and new insights into phylogenetic relationships
Source: BMC Genomics. 2022 Mar 21;23:223. doi: 10.1186/s12864-022-08454-3 (PMC8939231; doi:10.1186/s12864-022-08454-3)
Supplement: Supplementary file 3 — Additional file 3: Table S3. The indexes of the codon usage bias in Musaceae plastomes. [file 12864_2022_8454_MOESM3_ESM.docx]

| **Table S3** The indexes of the codon usage bias in Musaceae plastomes | | | | | | | |
| --- | --- | --- | --- | --- | --- | --- | --- |
| **Genus** | **Species** | **CDS length** | **Codon No.** | **ENC** | **CBI** | **GC2** | **GC3** |
| *Ensete* | *E. glaucum* | 87,735 | 29,245 | 49.67 | 0.355 | 0.373 | 0.268 |
|  | *E. livingstonianum* | 87,312 | 29,104 | 49.80 | 0.353 | 0.374 | 0.270 |
|  | *E. superbum* | 87,168 | 29,056 | 49.80 | 0.354 | 0.374 | 0.270 |
|  | *E. ventricosum* | 87,204 | 29,068 | 49.86 | 0.352 | 0.375 | 0.271 |
| *Musella* | *Musella lasiocarpa* | 87,426 | 29,142 | 49.56 | 0.357 | 0.372 | 0.268 |
| *Musa* | *M. acuminata* subsp. *banksii* | 88,089 | 29,363 | 49.53 | 0.358 | 0.374 | 0.268 |
|  | *M. acuminata* subsp. *burmannica* | 88,551 | 29,517 | 49.47 | 0.361 | 0.373 | 0.267 |
|  | *M. acuminata* subsp. *halabanensis* | 88,329 | 29,443 | 49.51 | 0.359 | 0.373 | 0.268 |
|  | *M. acuminata* subsp. *malaccensis* | 88,527 | 29,509 | 49.48 | 0.361 | 0.373 | 0.267 |
|  | *M. acuminata* subsp. *microcarpa* | 88,239 | 29,413 | 49.52 | 0.359 | 0.373 | 0.268 |
|  | *M. acuminata* subsp. *truncata* | 88,335 | 29,445 | 49.51 | 0.360 | 0.373 | 0.268 |
|  | *M. acuminata* subsp. *zebrina* | 88,347 | 29,449 | 49.51 | 0.360 | 0.373 | 0.268 |
|  | *M. aurantiaca* | 88,374 | 29,458 | 49.52 | 0.359 | 0.373 | 0.268 |
|  | *M. balbisiana* | 87,825 | 29,275 | 49.53 | 0.357 | 0.375 | 0.268 |
|  | *M. barioensis* | 87,411 | 29,137 | 49.48 | 0.358 | 0.373 | 0.267 |
|  | *M. basjoo* | 88,269 | 29,423 | 49.40 | 0.360 | 0.374 | 0.266 |
|  | *M. beccarii* | 87,648 | 29,216 | 49.48 | 0.359 | 0.373 | 0.266 |
|  | *M. borneensis* | 87,522 | 29,174 | 49.48 | 0.359 | 0.373 | 0.267 |
|  | *M. cheesmanii* | 88,152 | 29,384 | 49.50 | 0.358 | 0.374 | 0.268 |
|  | *M. chunii* | 86,400 | 28,800 | 49.57 | 0.357 | 0.376 | 0.269 |
|  | *M. coccinea* | 87,402 | 29,134 | 49.54 | 0.355 | 0.373 | 0.268 |
|  | *M. gracilis* | 87,438 | 29,146 | 49.49 | 0.358 | 0.373 | 0.267 |
|  | *M. ingens* | 87,594 | 29,198 | 49.46 | 0.360 | 0.372 | 0.266 |
|  | *M. itinerans* | 86,310 | 28,770 | 49.46 | 0.358 | 0.376 | 0.268 |
|  | *M. jackeyi* | 87,105 | 29,035 | 49.38 | 0.360 | 0.373 | 0.266 |
|  | *M. johnsii* | 87,366 | 29,122 | 49.51 | 0.357 | 0.374 | 0.267 |
|  | *M. laterita* | 88,362 | 29,454 | 49.50 | 0.360 | 0.373 | 0.268 |
|  | *M. lokok* | 87,624 | 29,208 | 49.49 | 0.359 | 0.373 | 0.267 |
|  | *M. lolodensis* | 87,732 | 29,244 | 49.46 | 0.359 | 0.373 | 0.267 |
|  | *M. maclayi* subsp. *maclayi* | 87,033 | 29,011 | 49.40 | 0.360 | 0.374 | 0.266 |
|  | *M. mannii* | 88,248 | 29,416 | 49.52 | 0.359 | 0.373 | 0.268 |
|  | *M. nagensium* | 88,305 | 29,435 | 49.39 | 0.362 | 0.374 | 0.266 |
|  | *M. ornata* | 88,212 | 29,404 | 49.55 | 0.358 | 0.374 | 0.268 |
|  | *M. paracoccinea* LSY001 | 87,489 | 29,163 | 49.54 | 0.356 | 0.372 | 0.267 |
|  | *M. paracoccinea* J52 | 87,348 | 29,116 | 49.56 | 0.355 | 0.373 | 0.268 |
|  | *M. peekelii* subsp. *angustigemma* | 87,012 | 29,004 | 49.40 | 0.360 | 0.374 | 0.266 |
|  | *M. puspanjaliae* | 88,248 | 29,416 | 49.36 | 0.362 | 0.373 | 0.265 |
|  | *M. rosea* | 88,221 | 29,407 | 49.53 | 0.359 | 0.374 | 0.268 |
|  | *M. rubinea* | 88,404 | 29,468 | 49.39 | 0.362 | 0.374 | 0.266 |
|  | *M. rubra* | 88,149 | 29,383 | 49.53 | 0.358 | 0.374 | 0.268 |
|  | *M. ruiliensis* | 88,062 | 29,354 | 49.51 | 0.359 | 0.374 | 0.268 |
|  | *M. salaccensis* | 87,333 | 29,111 | 49.51 | 0.357 | 0.374 | 0.267 |
|  | *M. sanguinea* | 88,032 | 29,344 | 49.55 | 0.358 | 0.374 | 0.268 |
|  | *M. schizocarpa* | 88,131 | 29,377 | 49.52 | 0.359 | 0.374 | 0.268 |
|  | *M. siamensis* | 88,173 | 29,391 | 49.52 | 0.359 | 0.374 | 0.268 |
|  | *M. tonkinensis* | 88,083 | 29,361 | 49.42 | 0.360 | 0.374 | 0.267 |
|  | *M. troglodytarum* | 87,258 | 29,086 | 49.36 | 0.361 | 0.373 | 0.266 |
|  | *M. velutina* | 87,777 | 29,259 | 49.53 | 0.359 | 0.374 | 0.268 |
|  | *M. yunnanensis* | 88,563 | 29,521 | 49.45 | 0.363 | 0.372 | 0.266 |
