# Supplementary material for: Comparative plastome analysis of Musaceae and new insights into phylogenetic relationships
Source: BMC Genomics. 2022 Mar 21;23:223. doi: 10.1186/s12864-022-08454-3 (PMC8939231; doi:10.1186/s12864-022-08454-3)
Supplement: Supplementary file 4 — Additional file 4: Table S4. Codon usage in Musaceae plastomes. [file 12864_2022_8454_MOESM4_ESM.docx]

| **Table S4** Codon usage in Musaceae plastomes | | | | | | | | | | | | | | | |
| --- | --- | --- | --- | --- | --- | --- | --- | --- | --- | --- | --- | --- | --- | --- | --- |
| **Codon** | **Aa** | **No.** | **RSCU** | **Codon** | **Aa** | **No.** | **RSCU** | **Codon** | **Aa** | **No.** | **RSCU** | **Codon** | **Aa** | **No.** | **RSCU** |
| UUU | Phe | 1019-1065 | 1.25-1.27 | UCU | Ser | 641-665 | 1.67-1.72 | UAU | Tyr | 847-877 | 1.57-1.59 | UGU | Cys | 241-258 | 1.44-1.51 |
| UUC |  | 590-616 | 0.73-0.75 | UCC |  | 361-385 | 0.94-1.00 | UAC |  | 225-242 | 0.41-0.43 | UGC |  | 83-93 | 0.49-0.56 |
| UUA | Leu | 889-911 | 1.81-1.85 | UCA |  | 445-481 | 1.18-1.25 | UAA* | Stop | 43-49 | 1.45-1.62 | UGA* | Stop | 19-23 | 0.63-0.78 |
| UUG |  | 610-635 | 1.25-1.29 | UCG |  | 204-220 | 0.53-0.57 | UAG* |  | 20-25 | 0.67-0.83 | UGG | Trp | 486-500 | 1.00 |
| CUU |  | 575-600 | 1.18-1.21 | CCU | Pro | 455-469 | 1.53-1.57 | CAU | His | 526-549 | 1.53-1.56 | CGU | Arg | 381-404 | 1.28-1.33 |
| CUC |  | 204-217 | 0.42-0.44 | CCC |  | 232-250 | 0.78-0.83 | CAC |  | 150-164 | 0.44-0.47 | CGC |  | 89-104 | 0.30-0.35 |
| CUA |  | 409-444 | 0.85-0.89 | CCA |  | 345-360 | 1.17-1.20 | CAA | Gln | 768-839 | 1.53-1.56 | CGA |  | 401-412 | 1.33-1.38 |
| CUG |  | 197-209 | 0.40-0.42 | CCG |  | 131-144 | 0.44-0.48 | CAG |  | 232-241 | 0.44-0.47 | CGG |  | 132-145 | 0.44-0.48 |
| AUU | Ile | 1191-1248 | 1.43-1.46 | ACU | Thr | 543-556 | 1.48-1.51 | AAU | Asn | 1052-1183 | 1.52-1.56 | AGU | Ser | 468-507 | 1.23-1.31 |
| AUC |  | 478-497 | 0.57-0.59 | ACC |  | 280-295 | 0.77-0.80 | AAC |  | 327-355 | 0.44-0.48 | AGC |  | 110-123 | 0.29-0.32 |
| AUA |  | 796-829 | 0.96-0.99 | ACA |  | 444-473 | 1.22-1.28 | AAA | Lys | 1181-1265 | 1.47-1.50 | AGA | Arg | 565-594 | 1.90-1.96 |
| AUG | Met | 675-695 | 1.00 | ACG |  | 165-181 | 0.45-0.50 | AAG |  | 414-438 | 0.50-0.53 | AGG |  | 175-194 | 0.59-0.65 |
| GUU | Val | 550-570 | 1.40-1.44 | GCU | Ala | 637-656 | 1.77-1.83 | GAU | Asp | 983-1033 | 1.62-1.63 | GGU | Gly | 607-622 | 1.31-1.34 |
| GUC |  | 205-214 | 0.52-0.54 | GCC |  | 225-241 | 0.63-0.67 | GAC |  | 228-239 | 0.37-0.38 | GGC |  | 169-183 | 0.37-0.39 |
| GUA |  | 586-608 | 1.49-1.54 | GCA |  | 400-427 | 1.13-1.18 | GAA | Glu | 1199-1294 | 1.46-1.50 | GGA |  | 778-798 | 1.67-1.71 |
| GUG |  | 197-220 | 0.51-0.55 | GCG |  | 137-150 | 0.39-0.42 | GAG |  | 416-448 | 0.50-0.54 | GGG |  | 272-296 | 0.58-0.63 |
