# Supplementary material for: Comparative plastome analysis of Musaceae and new insights into phylogenetic relationships
Source: BMC Genomics. 2022 Mar 21;23:223. doi: 10.1186/s12864-022-08454-3 (PMC8939231; doi:10.1186/s12864-022-08454-3)
Supplement: Supplementary file 5 — Additional file 5: Table S5. Codons exhibited higher usages (RSCU) and lower usages (RSCU) in Musacompared to Ensete and Musella. [file 12864_2022_8454_MOESM5_ESM.docx]

| **Table S5** Codons exhibited higher usages (RSCU) and lower usages (RSCU) in *Musa* compared to *Ensete* and *Musella* | | | | | | | | | | | | | |
| --- | --- | --- | --- | --- | --- | --- | --- | --- | --- | --- | --- | --- | --- |
| ***Genus*** | **Species** | **Higher usage in *Musa*** | | | | | | | **Lower usage in *Musa*** | | | | |
|  |  | **UUG** | **GUG** | **GAA** | **CGU** | **AGA** | **GGU** | **GGA** | **ACG** | **GCC** | **GAG** | **CGC** | **GGG** |
| *Ensete* | *E. glaucum* | 1.25 | 0.51 | 1.47 | 1.29 | 1.90 | 1.32 | 1.68 | 0.48 | 0.67 | 0.53 | 0.33 | 0.62 |
|  | *E. livingstonianum* | 1.26 | 0.51 | 1.46 | 1.29 | 1.90 | 1.32 | 1.68 | 0.50 | 0.66 | 0.54 | 0.32 | 0.61 |
|  | *E. superbum* | 1.25 | 0.52 | 1.47 | 1.30 | 1.90 | 1.32 | 1.68 | 0.49 | 0.66 | 0.53 | 0.34 | 0.62 |
|  | *E. ventricosum* | 1.26 | 0.51 | 1.47 | 1.28 | 1.90 | 1.31 | 1.67 | 0.49 | 0.66 | 0.53 | 0.35 | 0.63 |
| *Musella* | *Musella lasiocarpa* | 1.26 | 0.52 | 1.47 | 1.30 | 1.92 | 1.32 | 1.70 | 0.48 | 0.66 | 0.53 | 0.32 | 0.61 |
| *Musa* | *M. acuminata* subsp. *banksii* | 1.28 | 0.55 | 1.48 | 1.32 | 1.94 | 1.33 | 1.70 | 0.45 | 0.63 | 0.52 | 0.31 | 0.59 |
|  | *M. acuminata* subsp. *burmannica* | 1.28 | 0.54 | 1.48 | 1.31 | 1.94 | 1.33 | 1.70 | 0.45 | 0.63 | 0.52 | 0.31 | 0.59 |
|  | *M. acuminata* subsp. *halabanensis* | 1.28 | 0.55 | 1.48 | 1.31 | 1.95 | 1.33 | 1.70 | 0.45 | 0.63 | 0.52 | 0.31 | 0.60 |
|  | *M. acuminata* subsp. *malaccensis* | 1.28 | 0.55 | 1.48 | 1.31 | 1.95 | 1.33 | 1.70 | 0.45 | 0.63 | 0.52 | 0.31 | 0.59 |
|  | *M. acuminata* subsp. *microcarpa* | 1.28 | 0.55 | 1.48 | 1.32 | 1.94 | 1.33 | 1.70 | 0.45 | 0.63 | 0.52 | 0.31 | 0.59 |
|  | *M. acuminata* subsp. *truncata* | 1.28 | 0.55 | 1.48 | 1.31 | 1.95 | 1.32 | 1.70 | 0.45 | 0.63 | 0.52 | 0.31 | 0.59 |
|  | *M. acuminata* subsp. *zebrina* | 1.28 | 0.55 | 1.48 | 1.32 | 1.94 | 1.33 | 1.70 | 0.45 | 0.63 | 0.52 | 0.31 | 0.59 |
|  | *M. aurantiaca* | 1.28 | 0.55 | 1.49 | 1.31 | 1.95 | 1.33 | 1.71 | 0.46 | 0.63 | 0.51 | 0.32 | 0.59 |
|  | *M. balbisiana* | 1.27 | 0.54 | 1.47 | 1.32 | 1.94 | 1.33 | 1.70 | 0.45 | 0.63 | 0.53 | 0.31 | 0.59 |
|  | *M. barioensis* | 1.28 | 0.54 | 1.49 | 1.32 | 1.96 | 1.33 | 1.68 | 0.47 | 0.65 | 0.51 | 0.31 | 0.60 |
|  | *M. basjoo* | 1.27 | 0.53 | 1.48 | 1.33 | 1.94 | 1.33 | 1.70 | 0.45 | 0.63 | 0.52 | 0.30 | 0.59 |
|  | *M. beccarii* | 1.28 | 0.53 | 1.49 | 1.32 | 1.95 | 1.33 | 1.69 | 0.47 | 0.65 | 0.51 | 0.32 | 0.60 |
|  | *M. borneensis* | 1.27 | 0.53 | 1.49 | 1.32 | 1.96 | 1.34 | 1.68 | 0.48 | 0.65 | 0.51 | 0.31 | 0.60 |
|  | *M. cheesmanii* | 1.28 | 0.53 | 1.47 | 1.32 | 1.94 | 1.32 | 1.70 | 0.46 | 0.64 | 0.53 | 0.30 | 0.60 |
|  | *M. chunii* | 1.28 | 0.54 | 1.49 | 1.31 | 1.92 | 1.33 | 1.71 | 0.45 | 0.63 | 0.51 | 0.32 | 0.59 |
|  | *M. coccinea* | 1.27 | 0.54 | 1.49 | 1.33 | 1.94 | 1.34 | 1.68 | 0.47 | 0.65 | 0.51 | 0.31 | 0.61 |
|  | *M. gracilis* | 1.28 | 0.54 | 1.49 | 1.32 | 1.95 | 1.33 | 1.69 | 0.48 | 0.65 | 0.51 | 0.32 | 0.60 |
|  | *M. ingens* | 1.27 | 0.54 | 1.48 | 1.33 | 1.94 | 1.33 | 1.68 | 0.47 | 0.64 | 0.52 | 0.32 | 0.61 |
|  | *M. itinerans* | 1.26 | 0.53 | 1.47 | 1.33 | 1.92 | 1.34 | 1.69 | 0.45 | 0.64 | 0.53 | 0.30 | 0.59 |
|  | *M. jackeyi* | 1.28 | 0.53 | 1.49 | 1.33 | 1.96 | 1.33 | 1.69 | 0.47 | 0.65 | 0.51 | 0.31 | 0.60 |
|  | *M. johnsii* | 1.28 | 0.54 | 1.49 | 1.32 | 1.96 | 1.33 | 1.69 | 0.47 | 0.66 | 0.51 | 0.32 | 0.61 |
|  | *M. laterita* | 1.28 | 0.55 | 1.48 | 1.31 | 1.95 | 1.33 | 1.70 | 0.45 | 0.63 | 0.52 | 0.31 | 0.59 |
|  | *M. lokok* | 1.28 | 0.54 | 1.49 | 1.33 | 1.95 | 1.33 | 1.69 | 0.48 | 0.65 | 0.51 | 0.32 | 0.60 |
|  | *M. lolodensis* | 1.28 | 0.54 | 1.49 | 1.32 | 1.96 | 1.33 | 1.70 | 0.47 | 0.65 | 0.51 | 0.32 | 0.60 |
|  | *M. maclayi* subsp. *maclayi* | 1.28 | 0.53 | 1.49 | 1.33 | 1.96 | 1.33 | 1.69 | 0.47 | 0.65 | 0.51 | 0.31 | 0.60 |
|  | *M. mannii* | 1.28 | 0.54 | 1.48 | 1.31 | 1.95 | 1.33 | 1.70 | 0.46 | 0.63 | 0.52 | 0.32 | 0.59 |
|  | *M. nagensium* | 1.27 | 0.54 | 1.49 | 1.33 | 1.94 | 1.34 | 1.71 | 0.46 | 0.63 | 0.51 | 0.30 | 0.58 |
|  | *M. ornata* | 1.28 | 0.55 | 1.48 | 1.31 | 1.95 | 1.32 | 1.71 | 0.46 | 0.63 | 0.52 | 0.32 | 0.59 |
|  | *M. paracoccinea* LSY001 | 1.27 | 0.54 | 1.49 | 1.33 | 1.94 | 1.34 | 1.68 | 0.47 | 0.65 | 0.51 | 0.31 | 0.61 |
|  | *M. paracoccinea* J52 | 1.27 | 0.54 | 1.49 | 1.33 | 1.94 | 1.34 | 1.68 | 0.47 | 0.65 | 0.51 | 0.31 | 0.61 |
|  | *M. peekelii* subsp. *angustigemma* | 1.28 | 0.53 | 1.49 | 1.33 | 1.96 | 1.33 | 1.69 | 0.47 | 0.65 | 0.51 | 0.31 | 0.60 |
|  | *M. puspanjaliae* | 1.26 | 0.53 | 1.48 | 1.33 | 1.95 | 1.33 | 1.70 | 0.46 | 0.63 | 0.52 | 0.30 | 0.59 |
|  | *M. rosea* | 1.28 | 0.55 | 1.48 | 1.31 | 1.95 | 1.33 | 1.70 | 0.45 | 0.63 | 0.52 | 0.31 | 0.60 |
|  | *M. rubinea* | 1.27 | 0.54 | 1.49 | 1.33 | 1.94 | 1.33 | 1.71 | 0.46 | 0.63 | 0.51 | 0.30 | 0.59 |
|  | *M. rubra* | 1.28 | 0.55 | 1.48 | 1.31 | 1.95 | 1.33 | 1.70 | 0.45 | 0.63 | 0.52 | 0.31 | 0.59 |
|  | *M. ruiliensis* | 1.28 | 0.54 | 1.49 | 1.31 | 1.94 | 1.32 | 1.71 | 0.46 | 0.63 | 0.51 | 0.32 | 0.59 |
|  | *M. salaccensis* | 1.28 | 0.54 | 1.48 | 1.32 | 1.95 | 1.33 | 1.69 | 0.48 | 0.65 | 0.52 | 0.32 | 0.60 |
|  | *M. sanguinea* | 1.28 | 0.54 | 1.49 | 1.31 | 1.95 | 1.32 | 1.71 | 0.46 | 0.63 | 0.51 | 0.32 | 0.59 |
|  | *M. schizocarpa* | 1.28 | 0.53 | 1.48 | 1.31 | 1.94 | 1.33 | 1.70 | 0.46 | 0.64 | 0.52 | 0.31 | 0.59 |
|  | *M. siamensis* | 1.28 | 0.55 | 1.48 | 1.31 | 1.95 | 1.33 | 1.70 | 0.45 | 0.63 | 0.52 | 0.31 | 0.60 |
|  | *M. tonkinensis* | 1.27 | 0.54 | 1.48 | 1.33 | 1.95 | 1.34 | 1.70 | 0.46 | 0.63 | 0.52 | 0.30 | 0.59 |
|  | *M. troglodytarum* | 1.28 | 0.53 | 1.50 | 1.33 | 1.96 | 1.33 | 1.69 | 0.47 | 0.65 | 0.50 | 0.31 | 0.60 |
|  | *M. velutina* | 1.29 | 0.55 | 1.48 | 1.31 | 1.95 | 1.33 | 1.71 | 0.46 | 0.64 | 0.52 | 0.31 | 0.59 |
|  | *M. yunnanensis* | 1.27 | 0.54 | 1.49 | 1.32 | 1.95 | 1.32 | 1.71 | 0.46 | 0.64 | 0.51 | 0.31 | 0.60 |
