# Supplementary material for: Comparative plastome analysis of Musaceae and new insights into phylogenetic relationships
Source: BMC Genomics. 2022 Mar 21;23:223. doi: 10.1186/s12864-022-08454-3 (PMC8939231; doi:10.1186/s12864-022-08454-3)
Supplement: Supplementary file 6 — Additional file 6: Table S6. Frequency of short dispersed repeats (SDRs). [file 12864_2022_8454_MOESM6_ESM.docx]

| **Table S6** Frequency of short dispersed repeats (SDRs) | | | | | | | | | | |
| --- | --- | --- | --- | --- | --- | --- | --- | --- | --- | --- |
| **Species** | **Four SDR types** | | | | **Total** | **By length (bp)** | | | | |
|  | **Forward** | **Reverse** | **Complement** | **Palindromic** |  | **30-39** | **40-49** | **50-59** | **60-69** | **≥70** |
| *E. glaucum* | 32 | 0 | 0 | 39 | 71 | 26 | 23 | 4 | 0 | 18 |
| *E. livingstonianum* | 29 | 0 | 0 | 32 | 61 | 22 | 22 | 12 | 4 | 1 |
| *E. superbum* | 40 | 0 | 0 | 41 | 81 | 25 | 15 | 24 | 8 | 9 |
| *E. ventricosum* | 16 | 0 | 0 | 17 | 33 | 18 | 6 | 4 | 4 | 1 |
| *M. acuminata* subsp. *banksii* | 51 | 3 | 1 | 47 | 102 | 39 | 33 | 8 | 1 | 21 |
| *M. acuminata* subsp. *burmannica* | 69 | 2 | 1 | 61 | 133 | 44 | 31 | 16 | 4 | 38 |
| *M. acuminata* subsp. *halabanensis* | 58 | 2 | 1 | 43 | 104 | 46 | 28 | 14 | 1 | 15 |
| *M*. *acuminata* subsp. *malaccensis* | 66 | 3 | 1 | 59 | 129 | 41 | 42 | 12 | 1 | 33 |
| *M*. *acuminata* subsp. *microcarpa* | 55 | 1 | 0 | 45 | 101 | 38 | 34 | 12 | 1 | 16 |
| *M*. *acuminata* subsp. *truncata* | 59 | 3 | 1 | 51 | 114 | 45 | 34 | 12 | 1 | 22 |
| *M*. *acuminata* subsp. *zebrina* | 61 | 0 | 0 | 53 | 114 | 36 | 38 | 12 | 1 | 27 |
| *M. aurantiaca* | 93 | 2 | 1 | 84 | 180 | 92 | 32 | 20 | 13 | 23 |
| *M. balbisiana* | 51 | 3 | 1 | 45 | 100 | 61 | 24 | 4 | 5 | 6 |
| *M. barioensis* | 79 | 3 | 1 | 69 | 152 | 59 | 36 | 20 | 13 | 24 |
| *M. basjoo* | 84 | 24 | 11 | 72 | 191 | 94 | 59 | 21 | 6 | 11 |
| *M. beccarii* | 67 | 0 | 0 | 47 | 114 | 28 | 33 | 14 | 4 | 35 |
| *M. borneensis* | 83 | 2 | 1 | 76 | 162 | 57 | 37 | 24 | 18 | 26 |
| *M. cheesmanii* | 104 | 2 | 0 | 91 | 197 | 90 | 43 | 16 | 22 | 26 |
| *M. chunii* | 102 | 0 | 0 | 98 | 200 | 104 | 38 | 24 | 16 | 18 |
| *M. coccinea* | 32 | 0 | 0 | 20 | 52 | 14 | 16 | 5 | 4 | 13 |
| *M. gracilis* | 51 | 2 | 1 | 47 | 101 | 29 | 32 | 13 | 4 | 23 |
| *M. ingens* | 57 | 0 | 0 | 58 | 115 | 37 | 28 | 24 | 4 | 22 |
| *M. itinerans* | 51 | 2 | 1 | 48 | 102 | 60 | 24 | 9 | 0 | 9 |
| *M. jackeyi* | 47 | 0 | 0 | 37 | 84 | 30 | 27 | 9 | 1 | 17 |
| *M. johnsii* | 46 | 0 | 0 | 37 | 83 | 32 | 24 | 3 | 9 | 15 |
| *M. laterita* | 59 | 3 | 1 | 51 | 114 | 45 | 33 | 12 | 1 | 23 |
| *M. lokok* | 70 | 2 | 1 | 49 | 122 | 34 | 33 | 18 | 12 | 25 |
| *M. lolodensis* | 75 | 0 | 0 | 67 | 142 | 56 | 44 | 4 | 13 | 25 |
| *M. maclayi* subsp. *maclayi* | 47 | 0 | 0 | 37 | 84 | 32 | 31 | 10 | 2 | 9 |
| *M. mannii* | 117 | 3 | 1 | 112 | 233 | 124 | 39 | 24 | 17 | 29 |
| *M. nagensium* | 74 | 10 | 5 | 75 | 164 | 85 | 41 | 24 | 1 | 13 |
| *M. ornata* | 80 | 2 | 1 | 72 | 155 | 95 | 30 | 16 | 1 | 13 |
| *M. paracoccinea* LSY001 | 32 | 0 | 0 | 24 | 56 | 9 | 28 | 9 | 0 | 10 |
| *M. paracoccinea* J52 | 31 | 0 | 0 | 19 | 50 | 10 | 21 | 5 | 4 | 10 |
| *M. peekelii* subsp. *angustigemma* | 48 | 0 | 0 | 37 | 85 | 34 | 30 | 9 | 1 | 11 |
| *M. puspanjaliae* | 77 | 0 | 0 | 74 | 151 | 62 | 55 | 16 | 9 | 9 |
| *M. rosea* | 58 | 2 | 1 | 51 | 112 | 43 | 37 | 12 | 1 | 19 |
| *M. rubinea* | 85 | 15 | 7 | 77 | 184 | 106 | 27 | 17 | 5 | 29 |
| *M. rubra* | 51 | 2 | 1 | 43 | 97 | 43 | 29 | 13 | 1 | 11 |
| *M. ruiliensis* | 77 | 0 | 0 | 70 | 147 | 90 | 35 | 12 | 4 | 6 |
| *M. salaccensis* | 46 | 2 | 1 | 33 | 82 | 30 | 29 | 5 | 3 | 15 |
| *M. sanguinea* | 83 | 3 | 1 | 77 | 164 | 92 | 29 | 20 | 9 | 14 |
| *M. schizocarpa* | 74 | 1 | 0 | 75 | 150 | 79 | 34 | 12 | 9 | 16 |
| *M. siamensis* | 56 | 4 | 1 | 51 | 112 | 45 | 37 | 12 | 1 | 17 |
| *M. tonkinensis* | 64 | 23 | 0 | 47 | 134 | 58 | 31 | 30 | 10 | 5 |
| *M. troglodytarum* | 64 | 0 | 0 | 41 | 105 | 38 | 36 | 8 | 2 | 21 |
| *M. velutina* | 63 | 1 | 0 | 54 | 118 | 63 | 27 | 4 | 9 | 15 |
| *M. yunnanensis* | 121 | 26 | 13 | 115 | 275 | 140 | 73 | 28 | 13 | 21 |
| *Musella lasiocarpa* | 82 | 2 | 0 | 82 | 166 | 78 | 48 | 21 | 8 | 11 |
| Average | 63.61 | 3.16 | 1.14 | 56.12 | 124.04 | 54.24 | 32.98 | 13.82 | 5.73 | 17.27 |
