# Supplementary material for: Comparative plastome analysis of Musaceae and new insights into phylogenetic relationships
Source: BMC Genomics. 2022 Mar 21;23:223. doi: 10.1186/s12864-022-08454-3 (PMC8939231; doi:10.1186/s12864-022-08454-3)
Supplement: Supplementary file 7 — Additional file 7: Table S7. Number of simple sequence repeats (SSRs) in Musaceae plastomes. [file 12864_2022_8454_MOESM7_ESM.docx]

| **Table S7** Number of simple sequence repeats (SSRs) in Musaceae plastomes | | | | | | | | | | | | | | |
| --- | --- | --- | --- | --- | --- | --- | --- | --- | --- | --- | --- | --- | --- | --- |
| **Species** | **Six SSR types** | | | | | | **Total** | **By base composition** | | | | | | |
|  | **Mono-** | **Di-** | **Tri-** | **Tetra-** | **Penta-** | **Hexa-** |  | **A/T** | **G/C** | **A+T** | **A/T+**  **G/C** | **A+T+**  **G/C** | **A/T+**  **G+C** | **A+T+**  **G+C** |
| *E. glaucum* | 43 | 12 | 4 | 10 | 2 | 2 | 73 | 43 | 0 | 16 | 9 | 5 | 0 | 0 |
| *E. livingstonianum* | 35 | 10 | 7 | 11 | 2 | 1 | 66 | 35 | 0 | 16 | 10 | 5 | 0 | 0 |
| *E. superbum* | 35 | 11 | 9 | 14 | 7 | 0 | 76 | 33 | 2 | 24 | 12 | 5 | 0 | 0 |
| *E. ventricosum* | 38 | 10 | 7 | 11 | 3 | 1 | 70 | 38 | 0 | 18 | 10 | 4 | 0 | 0 |
| *M. acuminata* subsp. *banksii* | 33 | 12 | 10 | 13 | 4 | 2 | 74 | 33 | 0 | 22 | 14 | 3 | 2 | 0 |
| *M. acuminata* subsp. *burmannica* | 34 | 12 | 8 | 12 | 5 | 3 | 74 | 32 | 2 | 20 | 14 | 4 | 2 | 0 |
| *M. acuminata* subsp. *halabanensis* | 34 | 12 | 9 | 13 | 4 | 2 | 74 | 32 | 2 | 21 | 14 | 3 | 2 | 0 |
| *M. acuminata* subsp. *malaccensis* | 21 | 12 | 9 | 13 | 4 | 2 | 61 | 20 | 1 | 21 | 14 | 3 | 2 | 0 |
| *M. acuminata* subsp. *microcarpa* | 35 | 12 | 9 | 13 | 5 | 2 | 76 | 34 | 1 | 21 | 14 | 4 | 2 | 0 |
| *M. acuminata* subsp. *truncata* | 33 | 12 | 8 | 12 | 4 | 2 | 71 | 32 | 1 | 19 | 14 | 3 | 2 | 0 |
| *M. acuminata* subsp. *zebrina* | 35 | 12 | 9 | 13 | 3 | 2 | 74 | 35 | 0 | 21 | 14 | 2 | 2 | 0 |
| *M. aurantiaca* | 31 | 12 | 8 | 14 | 5 | 2 | 72 | 31 | 0 | 23 | 14 | 2 | 2 | 0 |
| *M. balbisiana* | 39 | 10 | 9 | 15 | 2 | 2 | 77 | 38 | 1 | 23 | 14 | 1 | 0 | 0 |
| *M. barioensis* | 37 | 10 | 10 | 13 | 2 | 3 | 75 | 37 | 0 | 19 | 12 | 4 | 3 | 0 |
| *M. basjoo* | 41 | 12 | 11 | 15 | 4 | 6 | 89 | 40 | 1 | 29 | 14 | 1 | 2 | 2 |
| *M. beccarii* | 38 | 10 | 9 | 13 | 1 | 3 | 74 | 38 | 0 | 18 | 12 | 4 | 2 | 0 |
| *M. borneensis* | 39 | 10 | 10 | 14 | 1 | 1 | 75 | 39 | 0 | 19 | 12 | 4 | 1 | 0 |
| *M. cheesmanii* | 37 | 11 | 11 | 13 | 2 | 13 | 87 | 36 | 1 | 23 | 13 | 14 | 0 | 0 |
| *M. chunii* | 30 | 11 | 8 | 13 | 4 | 2 | 68 | 30 | 0 | 21 | 14 | 1 | 2 | 0 |
| *M. coccinea* | 37 | 10 | 9 | 13 | 1 | 5 | 75 | 37 | 0 | 17 | 13 | 6 | 2 | 0 |
| *M. gracilis* | 39 | 10 | 9 | 12 | 1 | 1 | 72 | 39 | 0 | 17 | 12 | 4 | 0 | 0 |
| *M. ingens* | 40 | 9 | 10 | 15 | 1 | 3 | 78 | 39 | 1 | 18 | 13 | 5 | 2 | 0 |
| *M. itinerans* | 44 | 12 | 9 | 13 | 3 | 0 | 81 | 43 | 1 | 22 | 14 | 1 | 0 | 0 |
| *M. jackeyi* | 40 | 10 | 10 | 13 | 1 | 3 | 77 | 39 | 1 | 19 | 12 | 4 | 2 | 0 |
| *M. johnsii* | 42 | 10 | 9 | 12 | 1 | 3 | 77 | 41 | 1 | 17 | 12 | 6 | 0 | 0 |
| *M. laterita* | 34 | 12 | 9 | 13 | 4 | 2 | 74 | 33 | 1 | 21 | 14 | 3 | 2 | 0 |
| *M. lokok* | 38 | 10 | 8 | 10 | 2 | 1 | 69 | 38 | 0 | 23 | 5 | 3 | 0 | 0 |
| *M. lolodensis* | 37 | 10 | 10 | 13 | 1 | 3 | 74 | 37 | 0 | 19 | 12 | 4 | 2 | 0 |
| *M. maclayi* subsp. *maclayi* | 41 | 10 | 10 | 13 | 1 | 3 | 78 | 40 | 1 | 19 | 12 | 4 | 2 | 0 |
| *M. mannii* | 31 | 12 | 8 | 14 | 5 | 2 | 72 | 31 | 0 | 23 | 14 | 2 | 2 | 0 |
| *M. nagensium* | 42 | 12 | 7 | 17 | 1 | 6 | 85 | 42 | 0 | 22 | 16 | 1 | 4 | 0 |
| *M. ornata* | 31 | 13 | 9 | 12 | 5 | 3 | 73 | 31 | 0 | 24 | 13 | 3 | 2 | 0 |
| *M. paracoccinea* J52 | 39 | 10 | 7 | 13 | 1 | 4 | 74 | 39 | 0 | 16 | 14 | 3 | 2 | 0 |
| *M. paracoccinea* LSY001 | 39 | 10 | 9 | 14 | 1 | 3 | 76 | 39 | 0 | 18 | 14 | 3 | 2 | 0 |
| *M. peekelii* subsp. *angustigemma* | 41 | 10 | 10 | 13 | 1 | 3 | 78 | 40 | 1 | 19 | 12 | 4 | 2 | 0 |
| *M. puspanjaliae* | 52 | 11 | 10 | 15 | 2 | 4 | 94 | 52 | 0 | 23 | 16 | 1 | 2 | 0 |
| *M. rosea* | 34 | 12 | 8 | 11 | 4 | 2 | 71 | 33 | 1 | 18 | 14 | 3 | 2 | 0 |
| *M. rubinea* | 38 | 9 | 12 | 18 | 5 | 5 | 87 | 36 | 2 | 29 | 15 | 1 | 2 | 2 |
| *M. rubra* | 33 | 12 | 9 | 13 | 5 | 2 | 74 | 32 | 1 | 21 | 14 | 4 | 2 | 0 |
| *M. ruiliensis* | 29 | 12 | 8 | 12 | 4 | 2 | 67 | 29 | 0 | 21 | 14 | 1 | 2 | 0 |
| *M. salaccensis* | 40 | 10 | 10 | 13 | 1 | 1 | 75 | 40 | 0 | 19 | 12 | 4 | 0 | 0 |
| *M. sanguinea* | 32 | 12 | 8 | 13 | 5 | 2 | 72 | 32 | 0 | 23 | 13 | 2 | 2 | 0 |
| *M. schizocarpa* | 33 | 11 | 9 | 13 | 3 | 2 | 71 | 31 | 2 | 21 | 14 | 1 | 2 | 0 |
| *M. siamensis* | 35 | 12 | 10 | 13 | 4 | 2 | 76 | 34 | 1 | 22 | 14 | 3 | 2 | 0 |
| *M. tonkinensis* | 37 | 11 | 10 | 13 | 10 | 5 | 86 | 36 | 1 | 29 | 14 | 2 | 2 | 2 |
| *M. troglodytarum* | 40 | 10 | 10 | 13 | 1 | 3 | 77 | 39 | 1 | 19 | 12 | 4 | 2 | 0 |
| *M. velutina* | 38 | 12 | 9 | 14 | 2 | 2 | 77 | 38 | 0 | 22 | 13 | 2 | 2 | 0 |
| *M. yunnanensis* | 30 | 11 | 10 | 13 | 3 | 0 | 67 | 30 | 0 | 21 | 14 | 1 | 1 | 0 |
| *Musella lasiocarpa* | 32 | 9 | 7 | 16 | 16 | 2 | 82 | 32 | 0 | 28 | 13 | 9 | 0 | 0 |
| Total | 1786 | 537 | 438 | 645 | 159 | 130 | 3695 | 1758 | 28 | 1024 | 638 | 166 | 75 | 6 |
