# Supplementary material for: Comparative plastome analysis of Musaceae and new insights into phylogenetic relationships
Source: BMC Genomics. 2022 Mar 21;23:223. doi: 10.1186/s12864-022-08454-3 (PMC8939231; doi:10.1186/s12864-022-08454-3)
Supplement: Supplementary file 8 — Additional file 8: Table S8. Distribution of simple sequence repeats (SSRs) in Musaceae plastomes. [file 12864_2022_8454_MOESM8_ESM.docx]

| **Table S8** Distribution of simple sequence repeats (SSRs) in Musaceae plastomes | | | | | | | | | | | | |
| --- | --- | --- | --- | --- | --- | --- | --- | --- | --- | --- | --- | --- |
| **Species** | **Total** | **Coding** | **Non-**  **coding** | **IRa** | **IRb** | **SSC** | **LSC** | **SSRs in coding region** | | | | |
|  |  |  |  |  |  |  |  | ***rpoC2*** | ***rps14*** | ***ycf2*** | ***ycf1*** | ***ndhH*** |
| *E. glaucum* | 73 | 18 | 55 | 9 | 9 | 4 | 51 | 3 | 1 | 1 | 5 | 1 |
| *E. livingstonianum* | 66 | 18 | 48 | 9 | 9 | 4 | 44 | 3 | 1 | 1 | 5 | 1 |
| *E. superbum* | 76 | 16 | 60 | 6 | 6 | 5 | 59 | 3 | 1 | 1 | 4 | 1 |
| *E. ventricosum* | 70 | 18 | 52 | 7 | 7 | 4 | 52 | 3 | 1 | 1 | 5 | 1 |
| *M. acuminata* subsp. *banksii* | 74 | 21 | 53 | 10 | 10 | 8 | 46 | 2 | 1 | 1 | 7 | 1 |
| *M. acuminata* subsp. *burmannica* | 74 | 21 | 53 | 10 | 10 | 8 | 46 | 2 | 1 | 1 | 7 | 1 |
| *M. acuminata* subsp. *halabanensis* | 74 | 21 | 53 | 10 | 10 | 7 | 47 | 2 | 1 | 1 | 7 | 1 |
| *M. acuminata* subsp. *malaccensis* | 61 | 21 | 40 | 9 | 9 | 6 | 37 | 2 | 1 | 1 | 7 | 1 |
| *M. acuminata* subsp. *microcarpa* | 76 | 21 | 55 | 10 | 10 | 8 | 48 | 2 | 1 | 1 | 7 | 1 |
| *M. acuminata* subsp. *truncata* | 71 | 21 | 50 | 10 | 10 | 6 | 45 | 2 | 1 | 1 | 7 | 1 |
| *M. acuminata* subsp. *zebrina* | 74 | 21 | 53 | 11 | 11 | 7 | 45 | 2 | 1 | 1 | 7 | 1 |
| *M. aurantiaca* | 72 | 20 | 52 | 10 | 10 | 9 | 43 | 2 | 0 | 1 | 7 | 1 |
| *M. balbisiana* | 77 | 19 | 58 | 12 | 12 | 7 | 46 | 2 | 1 | 1 | 6 | 1 |
| *M. barioensis* | 75 | 24 | 51 | 11 | 11 | 6 | 47 | 3 | 1 | 1 | 8 | 1 |
| *M. basjoo* | 89 | 23 | 66 | 12 | 12 | 9 | 56 | 2 | 1 | 1 | 8 | 1 |
| *M. beccarii* | 74 | 24 | 50 | 11 | 11 | 7 | 45 | 3 | 1 | 1 | 8 | 1 |
| *M. borneensis* | 75 | 16 | 59 | 9 | 9 | 5 | 52 | 3 | 1 | 1 | 4 | 1 |
| *M. cheesmanii* | 87 | 32 | 55 | 17 | 17 | 9 | 44 | 2 | 0 | 1 | 13 | 1 |
| *M. chunii* | 68 | 21 | 47 | 10 | 10 | 7 | 41 | 2 | 1 | 1 | 7 | 1 |
| *M. coccinea* | 75 | 26 | 49 | 13 | 13 | 5 | 44 | 3 | 1 | 1 | 9 | 1 |
| *M. gracilis* | 72 | 24 | 48 | 11 | 11 | 7 | 43 | 3 | 1 | 1 | 8 | 1 |
| *M. ingens* | 78 | 20 | 58 | 9 | 9 | 5 | 55 | 3 | 1 | 1 | 6 | 1 |
| *M. itinerans* | 81 | 21 | 60 | 10 | 10 | 9 | 52 | 2 | 1 | 1 | 7 | 1 |
| *M. jackeyi* | 77 | 24 | 53 | 11 | 11 | 6 | 49 | 3 | 1 | 1 | 8 | 1 |
| *M. johnsii* | 77 | 24 | 53 | 11 | 11 | 5 | 50 | 3 | 1 | 2 | 7 | 1 |
| *M. laterita* | 74 | 21 | 53 | 10 | 10 | 7 | 47 | 2 | 1 | 1 | 7 | 1 |
| *M. lokok* | 69 | 24 | 45 | 11 | 11 | 6 | 41 | 3 | 1 | 1 | 8 | 1 |
| *M. lolodensis* | 74 | 24 | 50 | 11 | 11 | 5 | 47 | 3 | 1 | 1 | 8 | 1 |
| *M. maclayi* subsp. *maclayi* | 78 | 24 | 54 | 11 | 11 | 6 | 50 | 3 | 1 | 1 | 8 | 1 |
| *M. mannii* | 72 | 20 | 52 | 10 | 10 | 9 | 43 | 2 | 0 | 1 | 7 | 1 |
| *M. nagensium* | 85 | 27 | 58 | 13 | 13 | 9 | 50 | 2 | 1 | 1 | 10 | 1 |
| *M. ornata* | 73 | 21 | 52 | 9 | 9 | 9 | 46 | 2 | 1 | 1 | 7 | 1 |
| *M. paracoccine* J52 | 74 | 24 | 50 | 12 | 12 | 5 | 45 | 3 | 1 | 1 | 8 | 1 |
| *M. paracoccinea* LSY001 | 76 | 24 | 52 | 12 | 12 | 5 | 47 | 3 | 1 | 1 | 8 | 1 |
| *M. peekelii* subsp. *angustigemma* | 78 | 24 | 54 | 11 | 11 | 6 | 50 | 3 | 1 | 1 | 8 | 1 |
| *M. puspanjaliae* | 94 | 25 | 69 | 12 | 12 | 11 | 59 | 2 | 1 | 1 | 9 | 1 |
| *M. rosea* | 71 | 21 | 50 | 10 | 10 | 8 | 43 | 2 | 1 | 1 | 7 | 1 |
| *M. rubinea* | 87 | 23 | 64 | 13 | 13 | 8 | 53 | 2 | 1 | 1 | 8 | 1 |
| *M. rubra* | 74 | 21 | 53 | 10 | 10 | 7 | 47 | 2 | 1 | 1 | 7 | 1 |
| *M. ruiliensis* | 67 | 21 | 46 | 9 | 9 | 9 | 40 | 2 | 1 | 1 | 7 | 1 |
| *M. salaccensis* | 75 | 24 | 51 | 11 | 11 | 6 | 47 | 3 | 1 | 1 | 8 | 1 |
| *M. sanguinea* | 72 | 21 | 51 | 9 | 9 | 10 | 44 | 2 | 1 | 1 | 7 | 1 |
| *M. schizocarpa* | 71 | 21 | 50 | 10 | 10 | 6 | 45 | 2 | 1 | 1 | 7 | 1 |
| *M. siamensis* | 76 | 21 | 55 | 10 | 10 | 8 | 48 | 2 | 1 | 1 | 7 | 1 |
| *M. tonkinensis* | 86 | 21 | 65 | 11 | 11 | 10 | 54 | 2 | 1 | 1 | 7 | 1 |
| *M. troglodytarum* | 77 | 24 | 53 | 11 | 11 | 6 | 49 | 3 | 1 | 1 | 8 | 1 |
| *M. velutina* | 77 | 21 | 56 | 10 | 10 | 10 | 47 | 2 | 1 | 1 | 7 | 1 |
| *M. yunnanensis* | 67 | 20 | 47 | 9 | 9 | 8 | 41 | 3 | 1 | 1 | 6 | 1 |
| *Musella lasiocarpa* | 82 | 20 | 62 | 14 | 14 | 5 | 49 | 3 | 1 | 1 | 6 | 1 |
| Total | 3695 | 1072 | 2623 | 517 | 517 | 342 | 2319 | 120 | 46 | 50 | 354 | 49 |
