# Supplementary material for: Comparative plastome analysis of Musaceae and new insights into phylogenetic relationships
Source: BMC Genomics. 2022 Mar 21;23:223. doi: 10.1186/s12864-022-08454-3 (PMC8939231; doi:10.1186/s12864-022-08454-3)
Supplement: Supplementary file 9 — Additional file 9: Table S9. Frequency of tandem repeats by length. [file 12864_2022_8454_MOESM9_ESM.docx]

| **Table S9** Frequency of tandem repeats by length | | | | | | |
| --- | --- | --- | --- | --- | --- | --- |
| **Species** | **10-19** | **20-29** | **30-39** | **40-49** | **≥50** | **Total** |
| *E. glaucum* | 9 | 36 | 1 | 0 | 4 | 50 |
| *E. livingstonianum* | 6 | 33 | 2 | 0 | 0 | 41 |
| *E. superbum* | 6 | 24 | 8 | 1 | 3 | 42 |
| *E. ventricosum* | 7 | 34 | 4 | 1 | 2 | 48 |
| *M. acuminata* subsp. *banksii* | 12 | 58 | 7 | 3 | 5 | 85 |
| *M. acuminata* subsp. *burmannica* | 16 | 58 | 8 | 2 | 5 | 89 |
| *M. acuminata* subsp. *halabanensis* | 16 | 60 | 8 | 5 | 3 | 92 |
| *M. acuminata* subsp. *malaccensis* | 12 | 58 | 7 | 5 | 5 | 87 |
| *M. acuminata* subsp. *microcarpa* | 13 | 55 | 9 | 3 | 5 | 85 |
| *M. acuminata* subsp. *truncata* | 11 | 57 | 7 | 5 | 6 | 86 |
| *M. acuminata* subsp. *zebrina* | 12 | 56 | 7 | 3 | 3 | 81 |
| *M. aurantiaca* | 12 | 56 | 4 | 6 | 2 | 80 |
| *M. balbisiana* | 18 | 43 | 13 | 1 | 4 | 79 |
| *M. barioensis* | 10 | 32 | 8 | 4 | 3 | 57 |
| *M. basjoo* | 23 | 67 | 18 | 7 | 8 | 123 |
| *M. beccarii* | 14 | 31 | 7 | 3 | 0 | 55 |
| *M. borneensis* | 14 | 35 | 8 | 3 | 2 | 62 |
| *M. cheesmanii* | 10 | 74 | 12 | 5 | 4 | 105 |
| *M. chunii* | 8 | 59 | 7 | 3 | 5 | 82 |
| *M. coccinea* | 3 | 29 | 5 | 1 | 5 | 43 |
| *M. gracilis* | 14 | 32 | 9 | 3 | 1 | 59 |
| *M. ingens* | 14 | 31 | 7 | 2 | 0 | 54 |
| *M. itinerans* | 12 | 42 | 11 | 2 | 6 | 73 |
| *M. jackeyi* | 13 | 31 | 8 | 4 | 2 | 58 |
| *M. johnsii* | 11 | 30 | 7 | 4 | 1 | 53 |
| *M. laterita* | 12 | 58 | 7 | 4 | 5 | 86 |
| *M. lokok* | 13 | 29 | 7 | 5 | 1 | 55 |
| *M. lolodensis* | 13 | 33 | 8 | 3 | 3 | 60 |
| *M. maclayi* subsp. *maclayi* | 12 | 33 | 8 | 3 | 3 | 59 |
| *M. mannii* | 12 | 58 | 4 | 6 | 7 | 87 |
| *M. nagensium* | 13 | 59 | 13 | 2 | 6 | 93 |
| *M. ornata* | 15 | 64 | 9 | 6 | 4 | 98 |
| *M. paracoccinea* J52 | 3 | 28 | 3 | 1 | 1 | 36 |
| *M. paracoccinea* LSY001 | 6 | 29 | 6 | 0 | 2 | 43 |
| *M. peekelii* subsp*. angustigemma* | 15 | 32 | 9 | 4 | 2 | 62 |
| *M. puspanjaliae* | 13 | 61 | 15 | 5 | 2 | 96 |
| *M. rosea* | 10 | 51 | 7 | 3 | 3 | 74 |
| *M. rubinea* | 21 | 76 | 14 | 10 | 7 | 128 |
| *M. rubra* | 11 | 56 | 8 | 4 | 3 | 82 |
| *M. ruiliensis* | 9 | 56 | 10 | 4 | 4 | 83 |
| *M. salaccensis* | 15 | 33 | 10 | 3 | 6 | 67 |
| *M. sanguinea* | 10 | 58 | 7 | 4 | 5 | 84 |
| *M. schizocarpa* | 7 | 43 | 2 | 4 | 1 | 57 |
| *M. siamensis* | 13 | 59 | 8 | 4 | 3 | 87 |
| *M. tonkinensis* | 14 | 58 | 7 | 6 | 3 | 88 |
| *M. troglodytarum* | 13 | 32 | 8 | 4 | 2 | 59 |
| *M. velutina* | 15 | 58 | 9 | 3 | 2 | 87 |
| *M. yunnanensis* | 16 | 53 | 5 | 3 | 1 | 78 |
| *Musella lasiocarpa* | 20 | 40 | 8 | 0 | 1 | 69 |
| Average | 12.18 | 46.49 | 7.84 | 3.41 | 3.29 | 73.20 |
