# Supplementary material for: Comparative plastome analysis of Musaceae and new insights into phylogenetic relationships
Source: BMC Genomics. 2022 Mar 21;23:223. doi: 10.1186/s12864-022-08454-3 (PMC8939231; doi:10.1186/s12864-022-08454-3)
Supplement: Supplementary file 10 — Additional file 10: Table S10. Distribution of tandem repeats in Musaceae plastomes. [file 12864_2022_8454_MOESM10_ESM.docx]

| **Table S10** Distribution of tandem repeats in Musaceae plastomes | | | | | | | | | | | | | |
| --- | --- | --- | --- | --- | --- | --- | --- | --- | --- | --- | --- | --- | --- |
| **Species** | **Total** | **Coding** | **Non-**  **coding** | **IRa** | **IRb** | **SSC** | **LSC** | **Tandem repeats in coding region** | | | | | |
|  |  |  |  |  |  |  |  | ***rpoC2*** | ***accD*** | ***rpl20*** | ***rps11*** | ***ycf2*** | ***ycf1*** |
| *E. glaucum* | 50 | 30 | 20 | 15 | 15 | 0 | 20 | 0 | 3 | 0 | 1 | 5 | 8 |
| *E. livingstonianum* | 41 | 17 | 24 | 10 | 10 | 2 | 19 | 0 | 2 | 0 | 1 | 2 | 5 |
| *E. superbum* | 42 | 15 | 27 | 11 | 11 | 1 | 19 | 0 | 2 | 0 | 1 | 2 | 4 |
| *E. ventricosum* | 48 | 18 | 30 | 10 | 10 | 1 | 27 | 0 | 3 | 0 | 1 | 2 | 5 |
| *M. acuminata* subsp. *banksii* | 85 | 34 | 51 | 23 | 23 | 3 | 36 | 0 | 4 | 0 | 0 | 7 | 8 |
| *M. acuminata* subsp. *burmannica* | 89 | 34 | 55 | 24 | 24 | 2 | 39 | 1 | 3 | 0 | 0 | 7 | 8 |
| *M. acuminata* subsp. *halabanensis* | 92 | 35 | 57 | 23 | 23 | 3 | 43 | 0 | 4 | 0 | 1 | 7 | 8 |
| *M*. *acuminata* subsp. *malaccensis* | 87 | 34 | 53 | 23 | 23 | 3 | 38 | 0 | 4 | 0 | 0 | 7 | 8 |
| *M*. *acuminata* subsp. *microcarpa* | 85 | 34 | 51 | 21 | 21 | 3 | 40 | 0 | 4 | 0 | 0 | 7 | 8 |
| *M*. *acuminata* subsp. *truncata* | 86 | 35 | 51 | 22 | 22 | 3 | 39 | 0 | 5 | 0 | 0 | 7 | 8 |
| *M*. *acuminata* subsp. *zebrina* | 81 | 34 | 47 | 22 | 22 | 2 | 35 | 0 | 4 | 0 | 0 | 7 | 8 |
| *M. aurantiaca* | 80 | 29 | 51 | 20 | 20 | 4 | 36 | 1 | 2 | 0 | 0 | 6 | 7 |
| *M. balbisiana* | 79 | 27 | 52 | 20 | 20 | 8 | 31 | 0 | 3 | 0 | 0 | 5 | 7 |
| *M. barioensis* | 57 | 24 | 33 | 16 | 16 | 2 | 23 | 0 | 1 | 0 | 1 | 3 | 8 |
| *M. basjoo* | 123 | 29 | 94 | 19 | 19 | 18 | 67 | 0 | 1 | 0 | 0 | 4 | 10 |
| *M. beccarii* | 55 | 22 | 33 | 15 | 15 | 2 | 23 | 0 | 1 | 0 | 1 | 3 | 7 |
| *M. borneensis* | 62 | 28 | 34 | 17 | 17 | 3 | 25 | 0 | 1 | 0 | 1 | 4 | 9 |
| *M. cheesmanii* | 105 | 31 | 74 | 24 | 24 | 13 | 44 | 0 | 1 | 0 | 0 | 4 | 11 |
| *M. chunii* | 82 | 26 | 56 | 22 | 22 | 3 | 35 | 0 | 2 | 0 | 0 | 4 | 8 |
| *M. coccinea* | 43 | 24 | 19 | 11 | 11 | 0 | 21 | 0 | 1 | 0 | 1 | 4 | 7 |
| *M. gracilis* | 59 | 24 | 35 | 16 | 16 | 6 | 21 | 0 | 1 | 0 | 1 | 3 | 8 |
| *M. ingens* | 54 | 22 | 32 | 15 | 15 | 4 | 20 | 0 | 1 | 0 | 1 | 4 | 6 |
| *M. itinerans* | 73 | 28 | 45 | 19 | 19 | 6 | 29 | 0 | 4 | 0 | 0 | 5 | 7 |
| *M. jackeyi* | 58 | 26 | 32 | 15 | 15 | 2 | 26 | 0 | 1 | 0 | 1 | 4 | 8 |
| *M. johnsii* | 53 | 22 | 31 | 14 | 14 | 2 | 23 | 0 | 1 | 0 | 1 | 2 | 8 |
| *M. laterita* | 86 | 34 | 52 | 22 | 22 | 3 | 39 | 0 | 4 | 0 | 0 | 7 | 8 |
| *M. lokok* | 55 | 25 | 30 | 16 | 16 | 3 | 20 | 0 | 1 | 0 | 0 | 4 | 8 |
| *M. lolodensis* | 60 | 27 | 33 | 16 | 16 | 1 | 27 | 0 | 1 | 1 | 1 | 3 | 9 |
| *M. maclayi* subsp. *maclayi* | 59 | 26 | 33 | 15 | 15 | 2 | 27 | 0 | 1 | 0 | 1 | 4 | 8 |
| *M. mannii* | 87 | 32 | 55 | 21 | 21 | 4 | 41 | 2 | 4 | 0 | 0 | 6 | 7 |
| *M. nagensium* | 93 | 31 | 62 | 19 | 19 | 10 | 45 | 0 | 3 | 0 | 0 | 4 | 10 |
| *M. ornata* | 98 | 36 | 62 | 22 | 22 | 6 | 48 | 0 | 6 | 0 | 0 | 7 | 8 |
| *M. paracoccinea* J52 | 36 | 22 | 14 | 10 | 10 | 1 | 15 | 0 | 1 | 0 | 1 | 3 | 7 |
| *M. paracoccinea* LSY001 | 43 | 22 | 21 | 10 | 10 | 1 | 22 | 0 | 1 | 0 | 1 | 3 | 7 |
| *M. peekelii* subsp. *angustigemma* | 62 | 26 | 36 | 15 | 15 | 5 | 27 | 0 | 1 | 0 | 1 | 4 | 8 |
| *M. puspanjaliae* | 96 | 33 | 63 | 23 | 23 | 7 | 43 | 0 | 3 | 0 | 0 | 4 | 11 |
| *M. rosea* | 74 | 35 | 39 | 23 | 23 | 2 | 26 | 0 | 4 | 0 | 1 | 7 | 8 |
| *M. rubinea* | 128 | 29 | 99 | 20 | 20 | 15 | 73 | 0 | 3 | 0 | 0 | 4 | 9 |
| *M. rubra* | 82 | 34 | 48 | 22 | 22 | 3 | 35 | 0 | 4 | 0 | 0 | 7 | 8 |
| *M. ruiliensis* | 83 | 28 | 55 | 19 | 19 | 4 | 41 | 0 | 2 | 0 | 0 | 4 | 9 |
| *M. salaccensis* | 67 | 24 | 43 | 17 | 17 | 6 | 27 | 0 | 1 | 0 | 1 | 3 | 8 |
| *M. sanguinea* | 84 | 29 | 55 | 18 | 18 | 7 | 41 | 0 | 5 | 0 | 0 | 5 | 7 |
| *M. schizocarpa* | 57 | 29 | 28 | 17 | 17 | 2 | 21 | 0 | 3 | 0 | 0 | 5 | 8 |
| *M. siamensis* | 87 | 34 | 53 | 23 | 23 | 5 | 36 | 0 | 4 | 0 | 0 | 7 | 8 |
| *M. tonkinensis* | 88 | 31 | 57 | 17 | 17 | 7 | 47 | 0 | 3 | 0 | 0 | 6 | 8 |
| *M. troglodytarum* | 59 | 26 | 33 | 15 | 15 | 2 | 27 | 0 | 1 | 0 | 1 | 4 | 8 |
| *M. velutina* | 87 | 29 | 58 | 20 | 20 | 10 | 37 | 0 | 3 | 0 | 0 | 6 | 7 |
| *M. yunnanensis* | 78 | 28 | 50 | 19 | 19 | 4 | 36 | 0 | 2 | 0 | 0 | 5 | 8 |
| *Musella lasiocarpa* | 69 | 26 | 43 | 18 | 18 | 3 | 30 | 0 | 3 | 0 | 1 | 5 | 6 |
| Total | 3587 | 1378 | 2209 | 884 | 884 | 209 | 1610 | 4 | 123 | 1 | 22 | 232 | 382 |
