# Supplementary material for: Comparative plastome analysis of Musaceae and new insights into phylogenetic relationships
Source: BMC Genomics. 2022 Mar 21;23:223. doi: 10.1186/s12864-022-08454-3 (PMC8939231; doi:10.1186/s12864-022-08454-3)
Supplement: Supplementary file 11 — Additional file 11: Table S11. The estimation of substitution rate and dN/dS. [file 12864_2022_8454_MOESM11_ESM.docx]

| **Table S11** The estimation of substitution rate and dN/dS | | | | | | | |
| --- | --- | --- | --- | --- | --- | --- | --- |
| **Genes** | **dN** | **dS** | **dN/dS** | **Genes** | **dN** | **dS** | **dN/dS** |
| *ycf2* | 0.0579 | 0.0131 | 4.43827 | *rpoC1* | 0.0189 | 0.0994 | 0.18969 |
| *ycf1* | 0.0991 | 0.0851 | 1.16398 | *rps3* | 0.0340 | 0.1863 | 0.18219 |
| *rps7* | 0.0109 | 0.0113 | 0.96644 | *atpH* | 0.0113 | 0.0674 | 0.16812 |
| *psaI* | 0.0275 | 0.0315 | 0.87319 | *psbM* | 0.0133 | 0.0822 | 0.16193 |
| *rpl2* | 0.0035 | 0.0046 | 0.75957 | *rps12* | 0.0072 | 0.0467 | 0.15436 |
| *rpl33* | 0.0536 | 0.0919 | 0.58342 | *ycf3* | 0.0109 | 0.0779 | 0.13945 |
| *accD* | 0.1617 | 0.2881 | 0.56140 | *rpl23* | 0.0092 | 0.0712 | 0.12942 |
| *petN* | 0.0530 | 0.0966 | 0.54853 | *rpoB* | 0.0165 | 0.1292 | 0.12760 |
| *ndhB* | 0.0037 | 0.0076 | 0.48916 | *atpA* | 0.0167 | 0.1313 | 0.12692 |
| *atpE* | 0.0237 | 0.0495 | 0.47909 | *atpI* | 0.0113 | 0.0902 | 0.12555 |
| *ccsA* | 0.0794 | 0.1737 | 0.45738 | *rpl14* | 0.0175 | 0.1580 | 0.11090 |
| *clpP* | 0.0442 | 0.0975 | 0.45331 | *rpl16* | 0.0159 | 0.1459 | 0.10911 |
| *matK* | 0.1004 | 0.2490 | 0.40320 | *atpB* | 0.0103 | 0.0964 | 0.10717 |
| *rps16* | 0.0471 | 0.1172 | 0.40223 | *rps14* | 0.0085 | 0.0796 | 0.10646 |
| *psbH* | 0.0365 | 0.0917 | 0.39767 | *ndhE* | 0.0256 | 0.2735 | 0.09365 |
| *psbK* | 0.0313 | 0.0830 | 0.37717 | *ndhJ* | 0.0110 | 0.1192 | 0.09223 |
| *ycf4* | 0.0367 | 0.0982 | 0.37395 | *petG* | 0.0145 | 0.1606 | 0.09023 |
| *rps18* | 0.0215 | 0.0630 | 0.34204 | *infA* | 0.0166 | 0.1949 | 0.08508 |
| *rbcL* | 0.0332 | 0.1033 | 0.32153 | *psbA* | 0.0094 | 0.1195 | 0.07834 |
| *rps8* | 0.0402 | 0.1268 | 0.31707 | *psbE* | 0.0060 | 0.0815 | 0.07402 |
| *rpoA* | 0.0482 | 0.1541 | 0.31261 | *psbC* | 0.0069 | 0.0941 | 0.07286 |
| *ndhH* | 0.0056 | 0.0198 | 0.28395 | *psaB* | 0.0058 | 0.0866 | 0.06675 |
| *rpl20* | 0.0375 | 0.1327 | 0.28250 | *rps11* | 0.0244 | 0.3807 | 0.06419 |
| *rpl36* | 0.0252 | 0.0960 | 0.26269 | *petD* | 0.0057 | 0.0966 | 0.05862 |
| *rps2* | 0.0249 | 0.0958 | 0.2597 | *psaA* | 0.0055 | 0.1021 | 0.05385 |
| *rpl22* | 0.0907 | 0.3491 | 0.25969 | *psbB* | 0.0054 | 0.1204 | 0.04519 |
| *rpoC2* | 0.0347 | 0.1338 | 0.25931 | *petB* | 0.0063 | 0.1541 | 0.04062 |
| *ndhK* | 0.0258 | 0.1002 | 0.25702 | *psaC* | 0.0056 | 0.2106 | 0.02677 |
| *atpF* | 0.0276 | 0.1091 | 0.25304 | *rpl32* | 0.0001 | 0.5919 | 0.0001 |
| *rps19* | 0.032 | 0.1307 | 0.24451 | *psbL* | 0 | 0.3373 | 0.0001 |
| *ndhC* | 0.0236 | 0.0985 | 0.24004 | *psbI* | 0 | 0.221 | 0.0001 |
| *psbD* | 0.0173 | 0.0724 | 0.23938 | *psbJ* | 0 | 0.1637 | 0.0001 |
| *ndhF* | 0.0991 | 0.4199 | 0.23594 | *psbN* | 0 | 0.1632 | 0.0001 |
| *petA* | 0.0238 | 0.1048 | 0.22749 | *psbT* | 0 | 0.1559 | 0.0001 |
| *cemA* | 0.025 | 0.1101 | 0.22744 | *psbZ* | 0 | 0.1488 | 0.0001 |
| *ndhD* | 0.0617 | 0.2813 | 0.21924 | *rps15* | 0 | 0.1362 | 0.0001 |
| *ndhA* | 0.0261 | 0.12 | 0.2175 | *petL* | 0 | 0.0952 | 0.0001 |
| *rps4* | 0.0228 | 0.1126 | 0.20256 | *psaJ* | 0 | 0.0864 | 0.0001 |
| *ndhI* | 0.0389 | 0.193 | 0.20158 | *psbF* | 0 | 0.035 | 0.0001 |
| *ndhG* | 0.0337 | 0.1748 | 0.19297 |  |  |  |  |
