# Supplementary material for: Comparative plastome analysis of Musaceae and new insights into phylogenetic relationships
Source: BMC Genomics. 2022 Mar 21;23:223. doi: 10.1186/s12864-022-08454-3 (PMC8939231; doi:10.1186/s12864-022-08454-3)
Supplement: Supplementary file 12 — Additional file 12: Table S12. Variability of 23 regions in Musaceae. [file 12864_2022_8454_MOESM12_ESM.docx]

| **Table S12** Variability of 23 regions in Musaceae | | | | | | | |  |
| --- | --- | --- | --- | --- | --- | --- | --- | --- |
| **Regions** | **Aligned length (bp)** | **Length (bp)** | **Variable sites** | | **Information sites** | | **Nucleotide diversity** | **Number of haplotype** |
|  |  |  | **number** | **%** | **number** | **%** |  |  |
| *accD* | 2,057 | 1140-1908 | 154 | 7.49 | 53 | 2.58 | 0.01904 | 31 |
| *ccsA+ccsA-ndhD* | 1,412 | 1157-1302 | 119 | 8.43 | 64 | 4.53 | 0.01481 | 36 |
| *matK-rps16* | 2055 | 1525-1681 | 171 | 8.32 | 90 | 4.38 | 0.01957 | 35 |
| *ndhA* | 2624 | 2149-2363 | 191 | 7.28 | 116 | 4.42 | 0.01439 | 34 |
| *ndhD+ndhD-psaC+psaC* | 1882 | 1873-1882 | 150 | 7.97 | 81 | 4.3 | 0.01274 | 32 |
| *ndhF* | 2343 | 2208-2325 | 277 | 11.82 | 140 | 5.96 | 0.02018 | 38 |
| *ndhF-trnL* | 2587 | 1281-1921 | 150 | 5.80 | 77 | 2.98 | 0.02470 | 38 |
| *psbE-petL* | 1817 | 1183-1556 | 120 | 6.60 | 67 | 3.69 | 0.01836 | 31 |
| *trnE-psbD* | 2370 | 1528-2022 | 129 | 5.44 | 72 | 3.04 | 0.01802 | 35 |
| *ycf1* | 6795 | 4992-6153 | 205 | 3.02 | 143 | 2.10 | 0.01004 | 35 |
| Combination four most  variable markers | 9042 | 6694-7556 | 752 | 8.32 | 360 | 3.98 | 0.02054 | 45 |
| *matK* | 1566 | 1527-1551 | 154 | 9.83 | 74 | 4.73 | 0.01310 | 30 |
| *rbcL* | 1484 | 1464-1484 | 43 | 2.90 | 22 | 1.48 | 0.00544 | 27 |
| *trnH-psbA* | 1266 | 224-970 | 15 | 1.18 | 6 | 0.47 | 0.02407 | 8 |
| Combination three universal markers | 4316 | 3224-3979 | 212 | 4.91 | 102 | 2.36 | 0.00973 | 34 |
| LSC | 101,309 | 86,881-89,995 | 4345 | 4.29 | 2174 | 2.15 | 0.00820 | 46 |
| IRb | 38,098 | 33,985-35,469 | 703 | 1.85 | 435 | 1.14 | 0.00389 | 41 |
| SSC | 14,195 | 10,413-11,767 | 1000 | 7.04 | 517 | 3.64 | 0.01671 | 44 |
| Whole plastome | 190,750 | 166,782-172,514 | 6904 | 3.62 | 3,637 | 1.91 | 0.00698 | 48 |
