# Supplementary material for: Comparative plastome analysis of Musaceae and new insights into phylogenetic relationships
Source: BMC Genomics. 2022 Mar 21;23:223. doi: 10.1186/s12864-022-08454-3 (PMC8939231; doi:10.1186/s12864-022-08454-3)
Supplement: Supplementary file 13 — Additional file 13: Table S13. The indel polymorphism of some pairwise species with minimal interspecific K2P distance based on the combination of four most variable markers. [file 12864_2022_8454_MOESM13_ESM.docx]

| **Table S13** The indel polymorphism of some pairwise species with minimal interspecific K2P distance based on the combination of four most variable markers | | | | |
| --- | --- | --- | --- | --- |
| **Species 1** | **Species 2** | **K2P Distance** | **Indel events** | **Total number of indel sites** |
| *Musa chunii* | *Musa ruiliensis* | 0 | 4 | 72 |
| *Musa maclayi* subsp. *maclayi* | *Musa troglodytarum* | 0.000180 | 2 | 257 |
| *Musa jackeyi* | *Musa troglodytarum* | 0.000180 | 3 | 155 |
| *Musa mannii* | *Musa aurantiaca* | 0.000180 | 2 | 48 |
| *Musa peekelii* subsp. *angustigemma* | *Musa troglodytarum* | 0.000180 | 4 | 286 |
| *Musa sanguinea* | *Musa ornata* | 0.000180 | 5 | 133 |
