# Supplementary material for: Comparative plastome analysis of Musaceae and new insights into phylogenetic relationships
Source: BMC Genomics. 2022 Mar 21;23:223. doi: 10.1186/s12864-022-08454-3 (PMC8939231; doi:10.1186/s12864-022-08454-3)
Supplement: Supplementary file 14 — Additional file 14: Table S14. List of taxa and sources of plant material analyzed, and GenBank accession numbers of plastome of taxa used in the present study. [file 12864_2022_8454_MOESM14_ESM.docx]

| **Table S14** List of taxa and sources of plant material analyzed, and GenBank accession numbers of plastome of taxa used in the present study. Accession number marked with asterisk were retrieved from GenBank. N/A, not available | | | | | |  |
| --- | --- | --- | --- | --- | --- | --- |
| **Taxon** | **GenBank accession** | | **SRA accession** | **Collector** | **Accession** |  |
| Family Musaceae A.L. de Juss  Genus: *Musa* L.  Section *Callimusa* Cheesman |  |  |  |  |  |  |
| *Musa jackeyi* | OK012355 | | SRR8879202 | M. Häkkinen | ITC0588 |  |
| *Musa johnsii* | OK012354 | | SRR8879203 | M. Häkkinen | N/A |  |
| *Musa lolodensis* | OK012350 |  | SRR8879181 | M. Häkkinen | ITC0956 |  |
| *Musa maclayi* subsp. *maclayi* | OK012349 |  | SRR8879179 | M. Häkkinen | ITC0915 |  |
| *Musa peekelii* subsp. *angustigemma* | OK012370 |  | SRR8879178 | M. Häkkinen | ITC0618 |  |
| *Musa troglodytarum* | OK012331 |  | SRR8879204 | M. Häkkinen | M1 |  |
| *Musa barioensis* | OK012367 | | SRR8879195 | M. Häkkinen | TY01 |  |
| *Musa beccarii* | OK012365 | | SRR8879193 | M. Häkkinen | TY02 |  |
| *Musa borneensis* | OK012364 | | SRR8879194 | N.C. Pei | Brunei 01 |  |
| *Musa coccinea* | OK012360 | | SRR8879200 | X.J. Ge | 2001-0387 No: 9. |  |
| *Musa gracilis* | OK012358 | | SRR8879197 | M. Häkkinen | SM254 |  |
| *Musa lokok* | OK012351 | | SRR8879180 | M. Häkkinen | M17 |  |
| *Musa paracoccinea* | OK012344 | | SRR8879183 | X.J. Ge | J52 |  |
| *Musa paracoccinea* | OK012343 | | SRR16267455 | S.Y. Liu | LSY001 |  |
| *Musa salaccensis* | OK012337 | | SRR8879206 | M. Häkkinen | HBG: 2003-0768 |  |
| *Musa ingens* | OK012356 | | SRR8879198 | M. Häkkinen | HBG: 2005-0375 |  |
| Section *Musa* |  |  |  |  |  |  |
| *Musa acuminata* subsp. *banksii* | OK012368 | | SRR8879172 | M. Häkkinen | ITC0623 |  |
| *Musa acuminata* subsp. *burmannica* | OK012363 | | SRR8879173 | M. Häkkinen | A10 |  |
| *Musa acuminata* subsp. *halabanensis* | OK012357 | | SRR8879212 | X.J. Ge | Y45 |  |
| *Musa acuminata* subsp*. malaccensis* | HF677508* | | N/A | N/A | N/A |  |
| *Musa acuminata* subsp. *microcarpa* | OK012347 | | SRR8879213 | M. Häkkinen | Y13 |  |
| *Musa acuminata* subsp. *truncata* | OK012330 | | SRR8879175 | S.P. Ning | N4 |  |
| *Musa acuminata* subsp. *zebrina* | OK012326 | | SRR7013756 | M. Rouard | SRX3946209 |  |
| *Musa balbisiana* | NC_028439* | | N/A | N/A | N/A |  |
| *Musa basjoo* | OK012366 | | SRR8879196 | X.J. Ge | N/A |  |
| *Musa itinerans* | NC_035723* | | N/A | N/A | N/A |  |
| *Musa nagensium* | OK012346 | | SRR8879185 | M. Häkkinen | HBG：2006-0700 |  |
| *Musa schizocarpa* | OK012335 | | SRR8879188 | M. Häkkinen | ITC0846 |  |
| *Musa tonkinensis* | OK012332 | | SRR8879191 | M. Häkkinen | *Musa tonkinensis* |  |
| *Musa yunnanensis* | OK012327 | | SRR8879207 | X.J. Ge | Ge XJ 696 |  |
| *Musa puspanjaliae* | OK012342 | | SRR16097564 | X.J. Ge | Ge-lz-038 |  |
| *Musa cheesmanii* | OK012362 | | SRR16097565 | X.J. Ge | Ge-lz-013 |  |
| *Musa aurantiaca* | OK012369 | | SRR8879176 | M. Häkkinen | HBG: 2007-0001 |  |
| *Musa chunii* | OK012361 | | SRR8879199 | X.J. Ge | G693 |  |
| *Musa laterita* | OK012353 | | SRR8879182 | M. Häkkinen | TY21 |  |
| *Musa mannii* | OK012348 | | SRR8879186 | M. Häkkinen | TY24 |  |
| *Musa ornata* | OK012345 | | SRR8879184 | M. Häkkinen | TY26 |  |
| *Musa rosea* | OK012341 | | SRR8879177 | M. Häkkinen | HBG: 2001-0401 |  |
| *Musa rubinea* | OK012340 | | SRR8879192 | M. Häkkinen | TY33 |  |
| *Musa rubra* | OK012339 | | SRR8879174 | M. Häkkinen | 2002-0895 No: 18. |  |
| *Musa ruiliensis* | OK012338 | | SRR8879201 | X.J. Ge | J22 |  |
| *Musa sanguinea* | OK012336 | | SRR8879187 | X.J. Ge | J20 |  |
| *Musa siamensis* | OK012334 | | SRR8879189 | M. Häkkinen | 2002-0844 No:1. |  |
| *Musa velutina* | OK012329 | | SRR8879205 | M. Häkkinen | HBG: 1998-0017 |  |
| Genus: *Ensete* Horan. |  |  |  |  |  |  |
| *Ensete livingstonianum* | OK012352 | | SRR8879210 | M. Häkkinen | ITC1389 |  |
| *Ensete glaucum* | OK012359 | | SRR8879211 | M. Häkkinen | ITC0775 |  |
| *Ensete superbum* | OK012333 | | SRR8879208 | M. Häkkinen | M9 |  |
| *Ensete ventricosum* | OK012328 | | SRR8879209 | M. Häkkinen | HBG: 2006-0703 |  |
| Genus: *Musella* (Franch.) Li |  |  |  |  |  |  |
| *Musella lasiocarpa* | NC_035637* | | N/A | N/A | N/A |  |
| Family Heliconiaceae Vines |  | |  |  |  |  |
| Genus: *Heliconia* L. |  | |  |  |  |  |
| *Heliconia collinsiana* | NC_020362* | | N/A | N/A | N/A |  |

**Table S14** Continued

| **Taxon** | **GenBank accession** |  | **SRA accession** | **Collector** | **Accession** |
| --- | --- | --- | --- | --- | --- |
| Family Lowiaceae Ridl. |  |  |  |  |  |
| Genus: *Orchidantha* N. E. Br. |  |  |  |  |  |
| *Orchidantha fimbriata* | KF601569* | | N/A | N/A | N/A |
| Family Strelitziaceae Hutch. |  |  |  |  |  |
| Genus: *Ravenala* Adans. |  |  |  |  |  |
| *Ravenala madagascariensis* | KF601568* | | N/A | N/A | N/A |
| Family Zingiberaceae Lindley |  |  |  |  |  |
| Genus: *Alpinia* Roxburgh |  |  |  |  |  |
| *Alpinia chinensis* | NC_050165* | | N/A | N/A | N/A |
| *Alpinia katsumadae* | NC_048461* | | N/A | N/A | N/A |
| *Alpinia pumila* | NC_048462* | | N/A | N/A | N/A |
| Genus: *Amomum* Roxburgh |  | |  |  |  |
| *Amomum compacta* | NC_036992* | | N/A | N/A | N/A |
| Genus: *Curcuma* L. |  |  |  |  |  |
| *Curcuma longa* | MK965541* |  | N/A | N/A | N/A |
| Genus: *Zingiber* Boehm. |  |  |  |  |  |
| *Zingiber officinale* | NC_044775* |  | N/A | N/A | N/A |
| Family Costaceae Nakai |  |  |  |  |  |
| Genus: *Costus* L. |  |  |  |  |  |
| *Costus viridis* | MK262733* |  | N/A | N/A | N/A |
| Genus: *Monocostus* K. Schum. |  |  |  |  |  |
| *Monocostus uniflorus* | KF601572* |  | N/A | N/A | N/A |
| Family Cannaceae Juss. |  |  |  |  |  |
| Genus: *Canna* L. |  |  |  |  |  |
| *Canna indica* | MN832865* | | N/A | N/A | N/A |
| Family Marantaceae R. Br. |  |  |  |  |  |
| Genus: *Maranta* L. |  |  |  |  |  |
| *Maranta leuconeura* | KF601571* | | N/A | N/A | N/A |
| Genus: *Thaumatococcus* Benth. |  |  |  |  |  |
| *Thaumatococcus daniellii* | KF601575* | | N/A | N/A | N/A |
| Family Hanguanaceae Airy Shaw |  |  |  |  |  |
| Genus: *Hanguana* Blume |  |  |  |  |  |
| *Hanguana malayana* | KT312930* | | N/A | N/A | N/A |
