# Supplementary material for: Comparative plastome analysis of Musaceae and new insights into phylogenetic relationships
Source: BMC Genomics. 2022 Mar 21;23:223. doi: 10.1186/s12864-022-08454-3 (PMC8939231; doi:10.1186/s12864-022-08454-3)
Supplement: Supplementary file 15 — Additional file 15: Table S15. The primers used for PCR in current study. [file 12864_2022_8454_MOESM15_ESM.docx]

| **Table S15** The primers used for PCR in current study | | | |
| --- | --- | --- | --- |
| **IR boundary** | **Primer** | | **Universality** |
|  | Reverse | Forward |  |
| JSB | ACAAATGGGGTTGGCATTGC | TCTGCAGCGATACAACAACG | A-B |
|  | GGGTCTGCTTATTCGTTTTTCCG | GGTGGTCTACGAGCTGCTG | B |
| JSA | TTCAGGTGGTCTACGAGCTG | CCTCAGCTTCATACGGCTCC | A-B |
|  | TTCAGGTGGTCTACGAGCTG | TGTTCGGTAATAACTCAGCCCT | B |
| JLB | ACCCTGTAGACCATCCCCAT | AACGTCCGCGAATCTGATCA | A |
| JLA | GTTGACAGTCAGGGTCGTGT | GCCGGATCTAAGTGTTGGCT | A |

A: *E. superbum*, *M. yunnanensis*, *M. cheesmanii*, *M. puspanjaliae*, *M. ingens*, *M. coccinea*, *M. rubra*, *M. gracilis*, *M. barioensis*, *M. beccarii*, *M. acuminata* subsp. *burmannica*, *M. maclayi* subsp. *maclayi*, *M. paracoccinea*, *M. basjoo*, *M. schizocarpa*, *M. mannii*; B: *M. velutina*; JLA: LSC/IRa junction; JLB: LSC/IRb junction; JSA: SSC/IRa junction; JSB: SSC/IRb junction.
