# Supplementary material for: Comparative plastome analysis of Musaceae and new insights into phylogenetic relationships
Source: BMC Genomics. 2022 Mar 21;23:223. doi: 10.1186/s12864-022-08454-3 (PMC8939231; doi:10.1186/s12864-022-08454-3)
Supplement: Supplementary file 16 — Additional file 16: Table S16. The sequences of PCR products generated in this study with their accession names in Science DB (available at https://www.doi.org/10.11922/sciencedb.01436). [file 12864_2022_8454_MOESM16_ESM.docx]

| **Table S16** The PCR products generated in this study with their accession names in Science DB (available at https://www.doi.org/10.11922/sciencedb.01436) | | | | |
| --- | --- | --- | --- | --- |
| **Species** | **PCR product** **accession name** | | | |
|  | **JLA** | **JLB** | **JSA** | **JSB** |
| *Ensete superbum* | JLA_E. superbum_F(R) | JLB_E. superbum_F(R) | JSA_E. superbum_F(R) | JSB_E. superbum_F(R) |
| *Musa acuminata* subsp. *burmannica* | JLA_M. a. ssp. burmannica_F(R) | JLB_M. a. ssp. burmannica_F(R) | JSA_M. a. ssp. burmannica_F(R) | JSB_M. a. ssp. burmannica_F(R) |
| *Musa barioensis* | JLA_M. barioensis_F(R) | JLB_M. barioensis_F(R) | JSA_M. barioensis_F(R) | JSB_M. barioensis_F(R) |
| *Musa basjoo* | JLA_M. basjoo_F(R) | JLB_M. basjoo_F(R) | JSA_M. basjoo_F(R) | JSB_M. basjoo_F(R) |
| *Musa beccarii* | JLA_M. beccarii_F(R) | JLB_M. beccarii_F(R) | JSA_M. beccarii_F(R) | JSB_M. beccarii_F(R) |
| *Musa cheesmanii* | JLA_M. cheesmanii_F(R) | JLB_M. cheesmanii_F(R) | JSA_M. cheesmanii_F(R) | JSB_M. cheesmanii_F(R) |
| *Musa coccinea* | JLA_M. coccinea_F(R) | JLB_M. coccinea_F(R) | JSA_M. coccinea_F(R) | JSB_M. coccinea_F(R) |
| *Musa gracilis* | JLA_M. gracilis_F(R) | JLB_M. gracilis_F(R) | JSA_M. gracilis_F(R) | JSB_M. gracilis_F(R) |
| *Musa ingens* | JLA_M. ingens_F(R) | JLB_M. ingens_F(R) | JSA_M. ingens_F(R) | JSB_M. ingens_F(R) |
| *Musa maclayi* subsp. *maclayi* | JLA_M. m. ssp. maclayi_F(R) | JLB_M. m. ssp. maclayi_F(R) | JSA_M. m. ssp. maclayi_F(R) | JSB_M. m. ssp. maclayi_F(R) |
| *Musa mannii* | JLA_M. mannii_F(R) | JLB_M. mannii_F(R) | JSA_M. mannii_F(R) | JSB_M. mannii_F(R) |
| *Musa paracoccinea* | JLA_M. paracoccinea_F(R) | JLB_M. paracoccinea_F(R) | JSA_M. paracoccinea_F(R) | JSB_M. paracoccinea_F(R) |
| *Musa puspanjaliae* | JLA_M. puspanjaliae_F(R) | JLB_M. puspanjaliae_F(R) | JSA_M. puspanjaliae_F(R) | JSB_M. puspanjaliae_F(R) |
| *Musa rubra* | JLA_M. rubra_F(R) | JLB_M. rubra_F(R) | JSA_M. rubra_F(R) | JSB_M. rubra_F(R) |
| *Musa schizocarpa* | JLA_M. schizocarpa_F(R) | JLB_M. schizocarpa_F(R) | JSA_M. schizocarpa_F(R) | JSB_M. schizocarpa_F(R) |
| *Musa velutina* | JLA_M. velutina_F(R) | JLB_M. velutina_F(R) | JSA_M. velutina_F(R) | JSB_M. velutina_F(R) |
| *Musa yunnanensis* | JLA_M. yunnanensis_F(R) | JLB_M. yunnanensis_F(R) | JSA_M. yunnanensis_F(R) | JSB_M. yunnanensis_F(R) |

JLA: LSC/IRa junction; JLB: LSC/IRb junction; JSA: SSC/IRa junction; JSB: SSC/IRb junction.
