# Supplementary material for: Comparative plastome analysis of Musaceae and new insights into phylogenetic relationships
Source: BMC Genomics. 2022 Mar 21;23:223. doi: 10.1186/s12864-022-08454-3 (PMC8939231; doi:10.1186/s12864-022-08454-3)
Supplement: Supplementary file 17 — Additional file 17: Table S17. Partition scheme of 79 coding sequences (CDSs) used in this study. [file 12864_2022_8454_MOESM17_ESM.docx]

| **Table S17** Partition scheme of 79 coding sequences (CDSs) used in this study | | |
| --- | --- | --- |
| Subset | Best Model | Partition scheme |
| 1 | GTR+I+G | *accD* |
| 2 | GTR+I | *infA*, *atpA*, *rpl16*, *psaC* |
| 3 | TVM+I | *atpB*, *ycf3*, *atpE*, *rps12* |
| 4 | TVM+I+G | *rps14*, *atpF*, *rps2*, *rps4*, *ndhH*, *rpoB*, *rpl14*, *rpoC1*, *petA* |
| 5 | K81UF+I | *atpH*, *psbB*, *psbC* |
| 6 | GTR+G | *psbF*, *petD*, *atpI* |
| 7 | GTR+G | *ccsA* |
| 8 | K81UF+G | *ndhI*, *cemA* |
| 9 | HKY+G | *clpP* |
| 10 | TIM+G | *matK* |
| 11 | GTR+I+G | *ndhA*, *ycf4*, *ndhC* |
| 12 | K81UF | *ndhB* |
| 13 | TVM+I+G | *ndhE*, *ndhD* |
| 14 | TVM+I+G | *ndhF* |
| 15 | HKY+G | *petG*, *ndhG* |
| 16 | TVM+I+G | *ndhJ*, *ndhK*, *ycf2* |
| 17 | TRN+I+G | *psbA*, *petB*, *psbJ*, *psbD* |
| 18 | HKY | *psaI*, *petL*, *psaJ*, *petN*, *psbH* |
| 19 | TVM+I+G | *psaA*, *psbN*, *psaB*, *psbE* |
| 20 | TVM+I | *psbT*, *psbL*, *psbM*, *psbK*, *psbZ*, *psbI* |
| 21 | K81UF+I+G | *rbcL* |
| 22 | TIM+I+G | *rps18*, *rps19*, *rpl20*, *rps3*, *rpl32* |
| 23 | TVM+I+G | *rpl22*, *ycf1* |
| 24 | HKY | *rpl2*, *rps7*, *rpl23* |
| 25 | TVM+G | *rps15*, *rpoA*, *rps8*, *rpl36*, *rpl33* |
| 26 | TIM+I+G | *rpoC2*, *rps16* |
| 27 | HKY+I+G | *rps11* |
