# Supplementary figures and images for: Comparative plastome analysis of Musaceae and new insights into phylogenetic relationships
Source: BMC Genomics. 2022 Mar 21;23:223. doi: 10.1186/s12864-022-08454-3 (PMC8939231; doi:10.1186/s12864-022-08454-3)

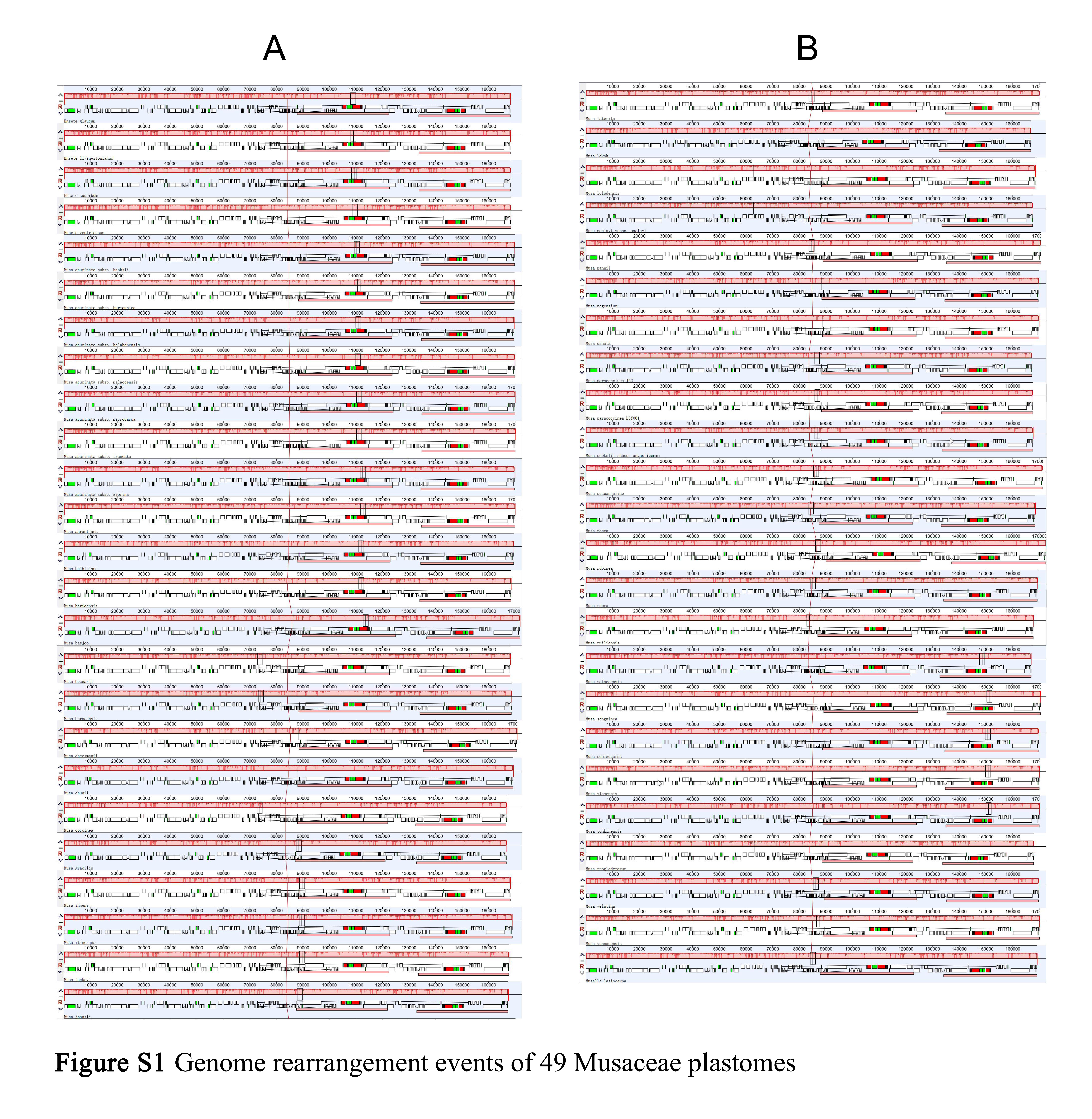

Supplement: Supplementary file 18 — Additional file 18: Figure S1. Genome rearrangement events of 49 Musaceae plastomes. [file 12864_2022_8454_MOESM18_ESM.jpg]

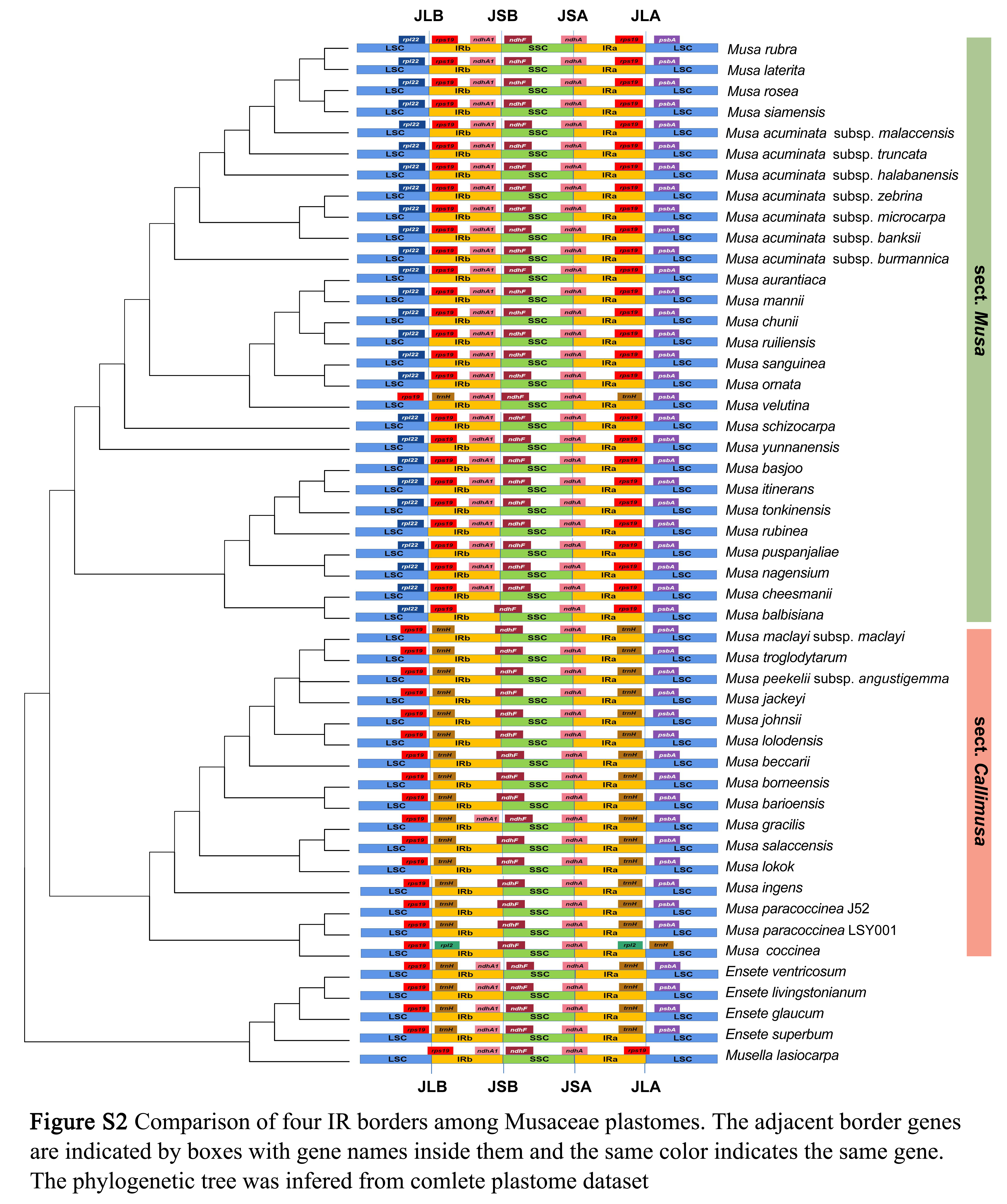

Supplement: Supplementary file 19 — Additional file 19: Figure S2. Comparison of four IR borders among Musaceae plastomes. [file 12864_2022_8454_MOESM19_ESM.png]

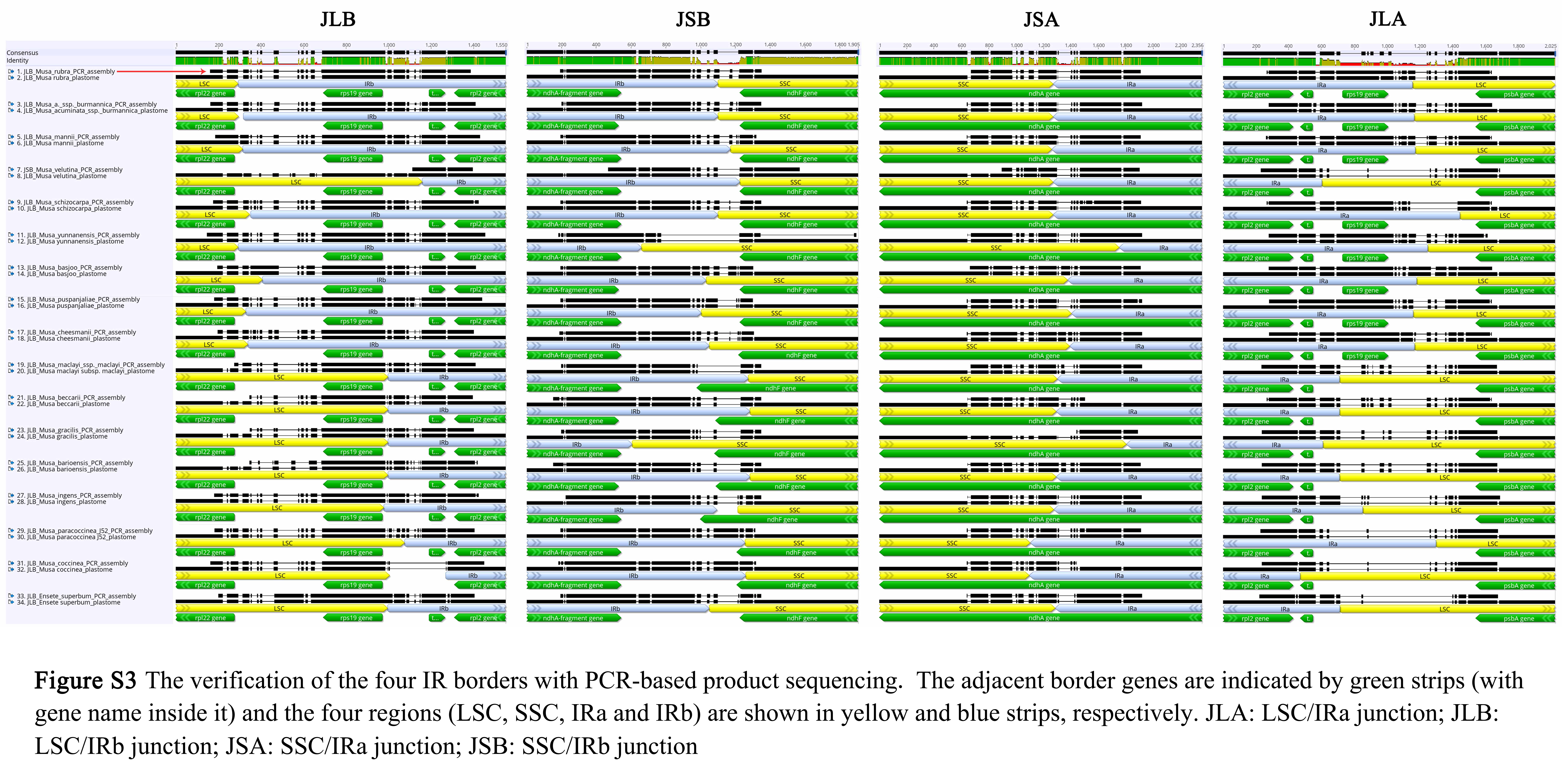

Supplement: Supplementary file 20 — Additional file 20: Figure S3. The verification of the four IR borders with PCR-based product sequencing. [file 12864_2022_8454_MOESM20_ESM.jpg]

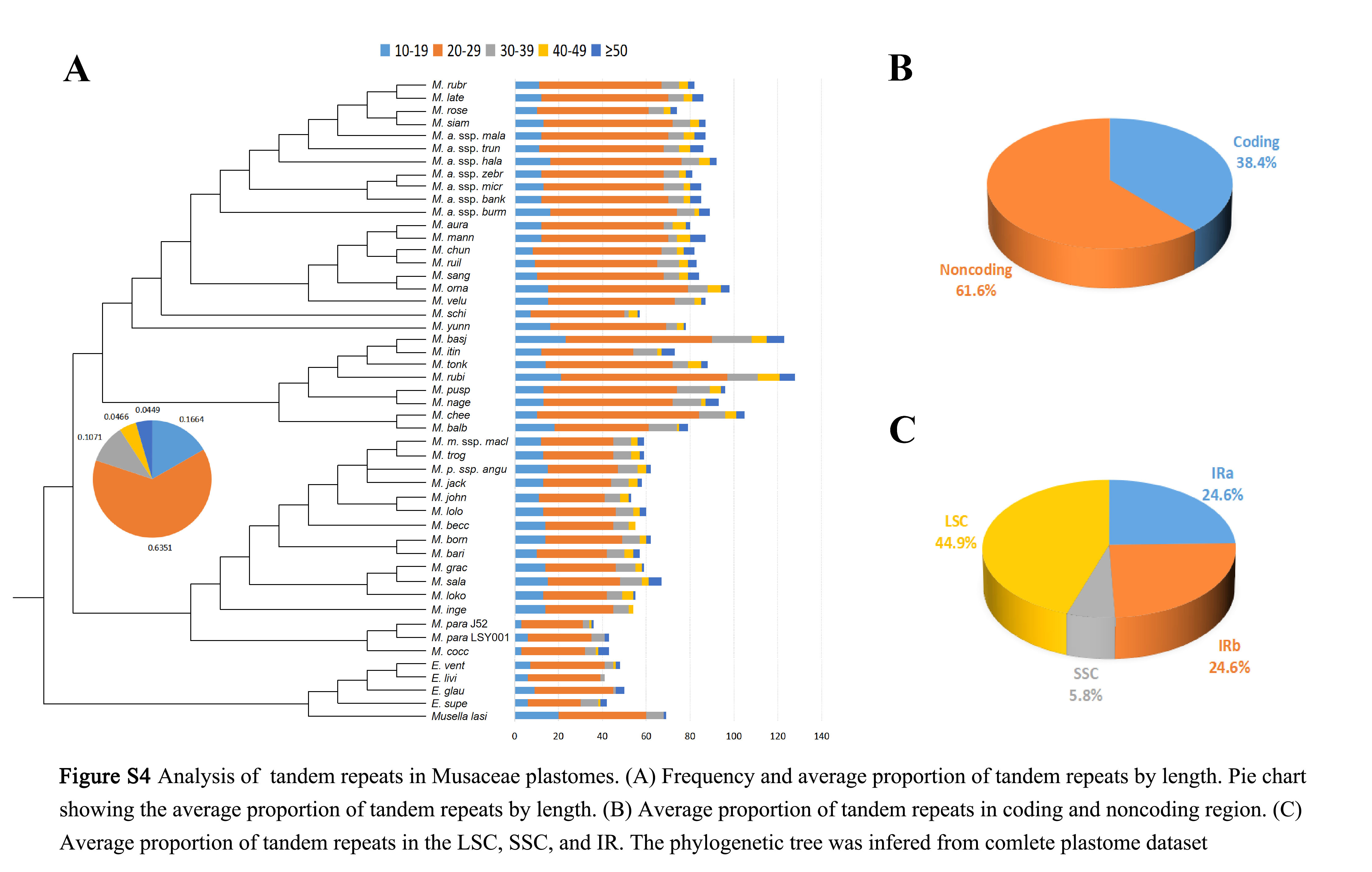

Supplement: Supplementary file 21 — Additional file 21: Figure S4. Analysis of tandem repeats in Musaceae plastomes. [file 12864_2022_8454_MOESM21_ESM.jpg]

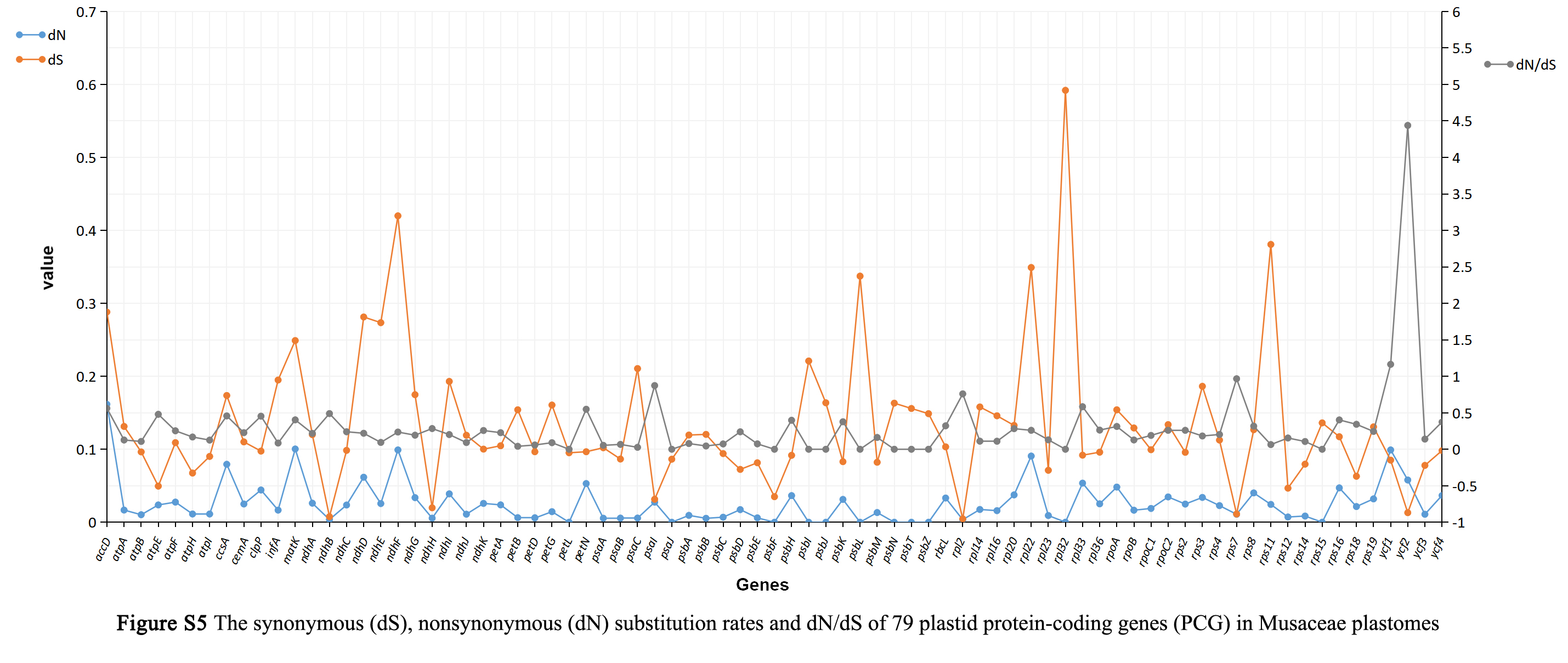

Supplement: Supplementary file 22 — Additional file 22: Figure S5. The synonymous (dS), nonsynonymous (dN) substitution rates and dN/dS of 79 plastid protein-coding genes (PCG) in Musaceae plastomes. [file 12864_2022_8454_MOESM22_ESM.jpg]

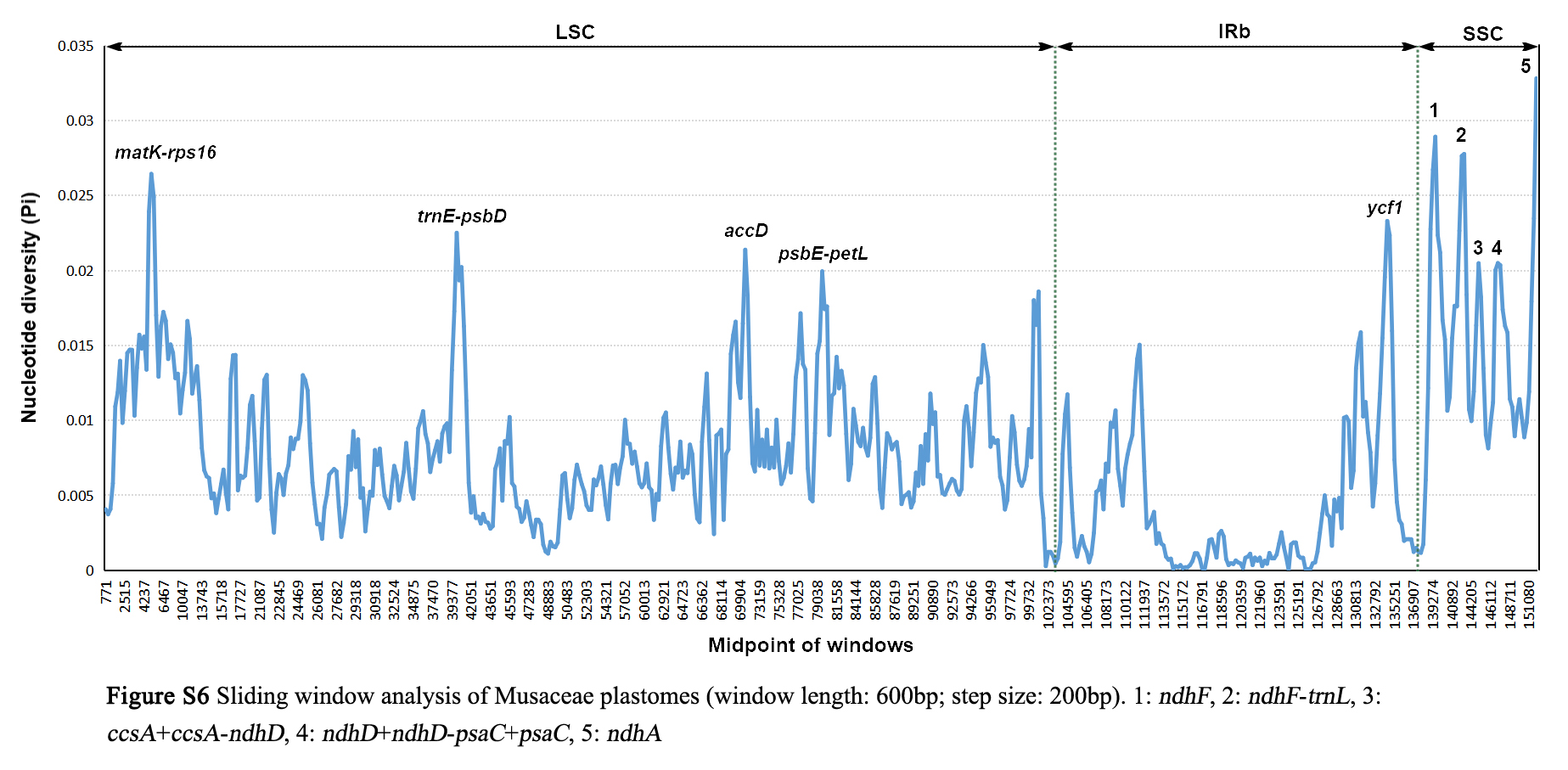

Supplement: Supplementary file 23 — Additional file 23: Figure S6. Sliding window analysis of Musaceae plastomes (window length: 600bp; step size: 200bp). [file 12864_2022_8454_MOESM23_ESM.jpg]

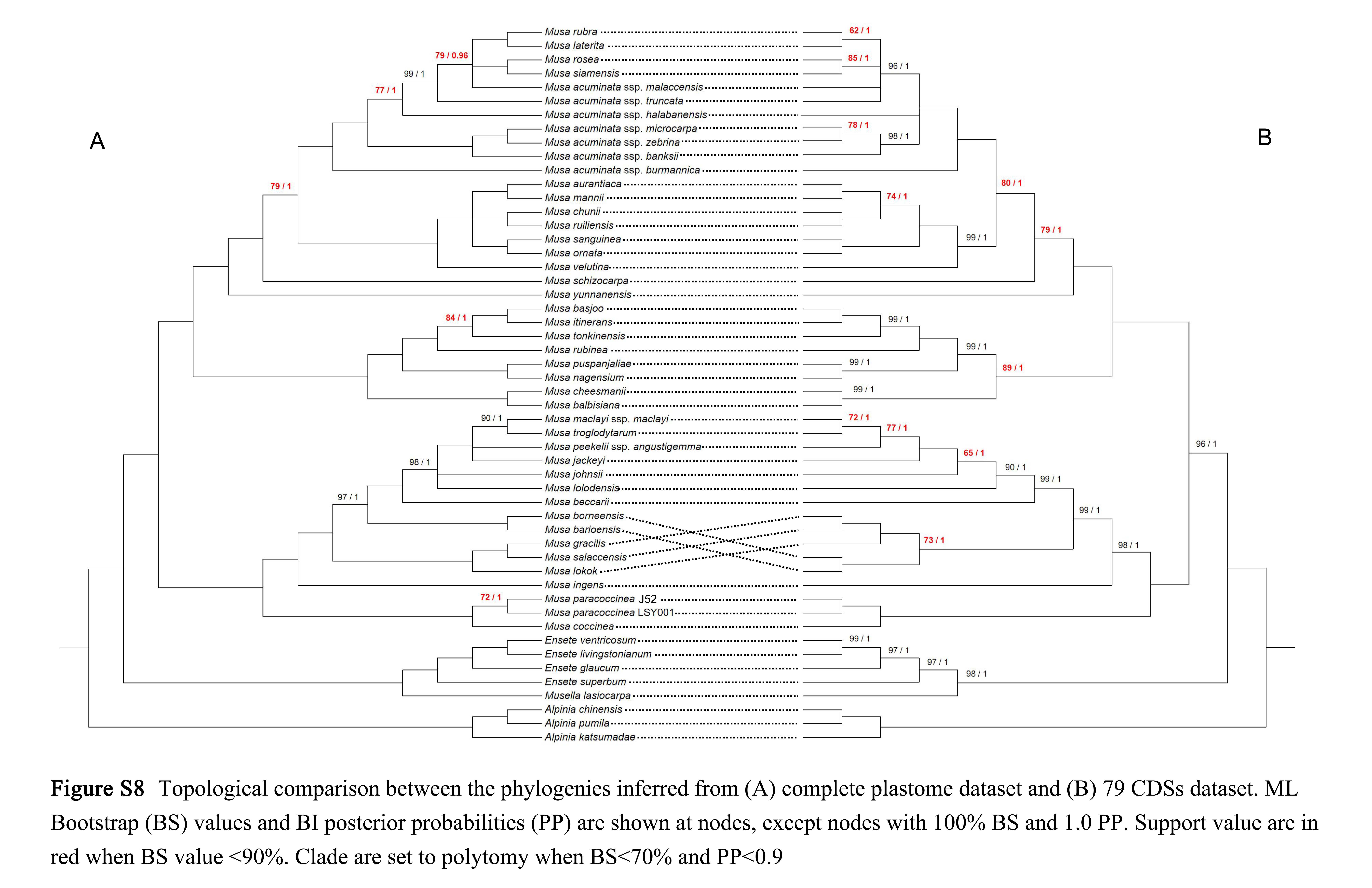

Supplement: Supplementary file 25 — Additional file 25: Figure S8. Topological comparison between the phylogenies infered from (A) complete plastome dataset and (B) 79 CDS dataset. [file 12864_2022_8454_MOESM25_ESM.png]

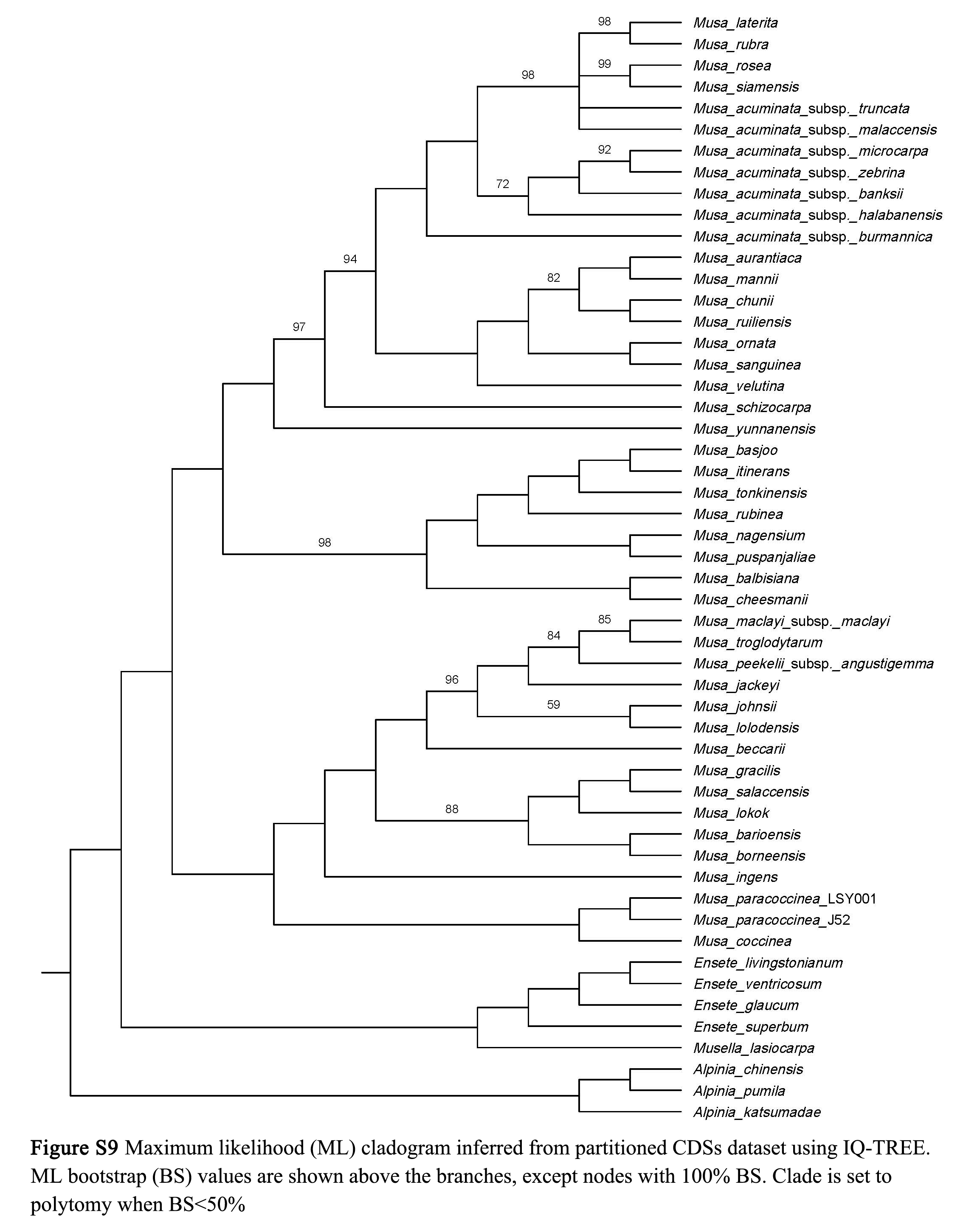

Supplement: Supplementary file 26 — Additional file 26: Figure S9. Maximum likelihood (ML) cladogram inferred from partitioned CDSs dataset using IQ-TREE. [file 12864_2022_8454_MOESM26_ESM.png]
